# Meta-analysis of binary outcomes via generalized linear mixed models: a simulation study

**DOI:** 10.1186/s12874-018-0531-9

**Published:** 2018-07-04

**Authors:** Ilyas Bakbergenuly, Elena Kulinskaya

**Affiliations:** School of Computing Sciences, University of East Anglia, Norwich, NR4 7TJ UK

**Keywords:** Generalized linear mixed-effects models, Random effects, Hypergeometric-normal likelihood, Transformation bias, Meta-analysis

## Abstract

**Background:**

Systematic reviews and meta-analyses of binary outcomes are widespread in all areas of application. The odds ratio, in particular, is by far the most popular effect measure. However, the standard meta-analysis of odds ratios using a random-effects model has a number of potential problems. An attractive alternative approach for the meta-analysis of binary outcomes uses a class of generalized linear mixed models (GLMMs). GLMMs are believed to overcome the problems of the standard random-effects model because they use a correct binomial-normal likelihood. However, this belief is based on theoretical considerations, and no sufficient simulations have assessed the performance of GLMMs in meta-analysis. This gap may be due to the computational complexity of these models and the resulting considerable time requirements.

**Methods:**

The present study is the first to provide extensive simulations on the performance of four GLMM methods (models with fixed and random study effects and two conditional methods) for meta-analysis of odds ratios in comparison to the standard random effects model.

**Results:**

In our simulations, the hypergeometric-normal model provided less biased estimation of the heterogeneity variance than the standard random-effects meta-analysis using the restricted maximum likelihood (REML) estimation when the data were sparse, but the REML method performed similarly for the point estimation of the odds ratio, and better for the interval estimation.

**Conclusions:**

It is difficult to recommend the use of GLMMs in the practice of meta-analysis. The problem of finding uniformly good methods of the meta-analysis for binary outcomes is still open.

**Electronic supplementary material:**

The online version of this article (10.1186/s12874-018-0531-9) contains supplementary material, which is available to authorized users.

## Background

Meta-analysis is a statistical technique for synthesizing outcomes from several studies. Since the individual studies might differ in populations and structure [1, 2], their effects are often assumed to be heterogeneous, and the use of methods based on random-effects models is recommended. When the outcome of interest is a transformation of a binomial outcome such as the logit transformation, the standard random-effects model assumes that within-study variability can be described by an approximate normal likelihood, i.e. the estimates of effects $\hat {\theta }_{i}\sim N\left (\theta _{i},\sigma ^{2}_{i}\right)$ in each study *i*, *i*=1…,*K*. Combining this assumption with a normal distribution of true effects between studies, *θ*_*i*_∼*N*(*θ*,*τ*^2^), the resulting marginal random-effects model is $\hat {\theta }_{i}\sim N\left (\theta,\sigma ^{2}_{i}+\tau ^{2}\right)$. However, the standard REM has several potential problems. It makes the strong assumption that the estimated within-study variances $\hat {\sigma }^{2}_{i}$ can be used in place of the unknown true variances $\sigma _{i}^{2}$ (without accounting for their variability), and it does not account for the correlation between the estimated within-study variances $\hat {\sigma }_{i}^{2}$ and the effect measures $\hat {\theta }_{i}$ [3–5]. Additionally, the standard REM suffers from transformation bias ([6]) and bias in the estimation of the random-effect variance *τ*^2^.

An attractive alternative approach for the meta-analysis of binary outcomes uses a class of generalized linear mixed models (GLMMs). These models can be fitted in SAS [3] and in R using the *metafor* package by Viechtbauer [7]. Generalized linear mixed models are believed to overcome the problems of the standard random-effects model [3] because they use a binomial-normal likelihood. However, this belief is based on theoretical considerations, and no sufficient simulations have assessed the performance of methods based on GLMMs in meta-analysis. This gap may be due to the computational complexity of these models and the resulting considerable time requirements for simulations.

We concentrate on the meta-analysis of odds ratios (OR), by far the most popular effect measure, with normally-distributed true effects *θ*_*i*_ between studies. Other mixing distributions for random effects are possible [8]. A natural alternative is a beta-binomial model, which assumes a beta mixing distribution for the event probabilities. This model was recommended for use with sparse data by Kuss [9] and studied in much detail in [10].

The relative risk (RR) is often a more appropriate measure of effect than the odds ratio, and it has a direct interpretation. Reasons for choosing RR instead of OR and the ease with which OR can be misinterpreted are discussed in [11–15]. However, perhaps due to the mathematical convenience and to the widely available software implementations, the odds ratio is by far the most popular effect measure. Our simulations have used all four GLMM methods available in *metafor*: GLMM with fixed or random study effects [16]; the noncentral-hypergeometric-normal model (NCHGN) discussed by Van Houwelingen et al. [17], Liu and Pierce [18], Sidik and Jonkman [19] and Stijnen et al. [3]; and an approximation of noncentral-hypergeometric-normal model by a binomial-normal model, method CM.AL in *metafor*. For comparison, we also included two standard inverse-variance weights based methods, DerSimonian-Laird (DL) [20] and restricted maximum likelihood (REML), routinely used in random-effects meta-analysis.

Among the GLMMs available for the meta-analysis of binary outcomes, we are particularly interested in the NCHGN. The exact distribution for the number of events conditional on marginal totals is the noncentral hypergeometric distribution. The NCHGN model also includes a normally distributed random effect (log odds ratio) for studies. However, the performance of this model is not well known. The simulation study on GLMMs in meta-analysis by Kuss [9] compared several methods for analysing sparse 2×2 data but excluded the NCHGN model and its approximation by the binomial-normal distribution as they exclude double-zero studies, i.e. studies with zero events in both arms. The recent simulation study by Jackson et al. [21] examined the use of seven GLMMs for summary odds ratio, including the NCHGN model and the other models considered in our study. However, Jackson et al. [21] considered only 15 configurations of the parameters, limited almost exclusively to *K*=10 studies, the baseline probability of 0.2 and the small value of *τ*^2^=0.024. We provide extensive simulations for 880 configurations of the parameters, including *K*=3, 5, 10 and 30 studies, the baseline probabilities from 0.1 to 0.4, and the heterogeneity variance *τ*^2^ from 0 to 1. The span of our simulations is instrumental in detecting important trends in performance of GLMMs for the meta-analysis of odds ratios.

Our simulation results demonstrate that the GLMM models including the NCHGN do not outperform the standard DL and REML methods in point and interval estimation of overall effect measure. Possible reasons to the unexpected inferior performance of GLMM methods are pointed out in the discussion. The structure of the rest of this paper is as follows.

“Methods” section reviews the GLMMs for binary outcomes and discusses likelihood-based models for log odds ratio. It also describes the simulation study. “Results” section presents the results of simulations and provides an illustrative example. “Discussion” section summarizes our results. “Conclusions” section provides further recommendations.

## Methods

### General formulation of generalized linear mixed models for meta-analysis of binary outcomes

The generalized linear mixed effects model (GLMM) extends the generalized linear model by including random effects in addition to fixed effects (hence mixed-effects model). The inference in GLMMs is based on the likelihood.

For the general case, let the univariate observation in the *i*^*th*^ study be *y*_*i*_, and the vectors of covariates *x*_*i*_ and *z*_*i*_ of dimensions *p* and *q* stand for fixed and random effects, respectively, for *i*=1,…,*K*. The responses *y*_*i*_ are assumed to be independent with conditional means E(*y*_*i*_|*b*_*i*_)=*μ*_*i*_(*b*_*i*_) and variances Var(*y*_*i*_|*b*_*i*_)=*Δa*_*i*_*υ*(*μ*_*i*_(*b*_*i*_)), where *Δ* is the dispersion parameter, *a*_*i*_ is a known constant, *b*_*i*_ is a random effect and *υ*(·) is a variance function [22]. The conditional mean and variance have a mean-variance relation, and both of them depend on a random effect *b*_*i*_. Given the q-dimensional vector of random effects *b*, the generalized linear mixed model has the form

1 $$ \eta_{i}^{b}(b)=x_{i}^{t}\beta+z_{i}^{t}b,  $$

where *β* is the vector of regression parameters and *t* is the matrix transpose. Similarly to the generalized linear model, the conditional mean is associated with a linear predictor through a link function *g*(*μ*_*i*_(*b*_*i*_))=*η*_*i*_(*b*_*i*_). Inverting the link function, *H*=*g*^−1^, and denoting the design matrices with rows $x_{i}^{t}$ and $z_{i}^{t}$ by *X* and *Z*, the conditional mean satisfies

$$\text{E}(y|b)=H(X\beta+Zb),$$

where *y*=(*y*_1_,…,*y*_*K*_). The random effect *b* follows a (usually multivariate normal) distribution with zero mean and with variance-covariance matrix *D*=*D*(*ζ*), for an unknown vector of variance components *ζ*. Breslow and Clayton [22] consider models with binomial, Poisson, and hypergeometric specifications for the conditional distribution of *y*_*i*_ and the dispersion parameter *Δ*=1 in the conditional variance. The value of *Δ*>1 is often used to model overdispersion, and *Δ* is estimated jointly with the parameters *ζ* in *D*=*D*(*ζ*).

In generalized linear mixed models, the parameters are estimated by maximum likelihood. However, because of nonlinearity of the model and the presence of random effects, the marginal distribution for the maximum-likelihood approach includes a cumbersome integration with respect to unobservable random effects. Usually, the integration does not have a closed form, and therefore no analytic solution is possible. Numerical methods such as adaptive Hermite quadrature (GHQ) and Laplace’s method have to be applied to evaluate the integral, approximation of the log-likelihood function, score equations, and information matrix [22]. Alternative estimation techniques include penalized quasi-likelihood method (PQL) [22], equivalent pseudo-likelihood method, and higher order Laplace approximations, see [23] for review. Alternatively, a Bayesian approach uses stochastic integration by Markov chain Monte Carlo (MCMC) or Gibbs sampling to fit GLMMs. Hybrid methods are also available [24]. The moment-based generalized estimation equation (GEE) method can also be used for population-average parameter estimation in the marginal models.

### GLMMs for the meta-analysis of odds ratios

For binary outcomes *y*_*i*_ and the logit link function *g*(·), the model (1) is a logistic regression model with random effects. In a meta-analysis, the study effects correspond to the intercept, and the treatment effect to the slope of treatment/control indicator in the logistic regression; the log odds ratio (LOR) is the difference between the log odds of the treatment and control groups. Platt et al. [25] and Gao [26] considered a generalized linear mixed model with a fixed treatment effect and a random intercept term for each study and provided some simulations on the use of a PQL, GHQ and a linear model fitted by weighted least squares. The use of this model for sparse data was further studied in the extensive simulation study by Kuss [9], who compared a large number of available fitting methods including a PQL, GHQ, MCMC, beta-binomial model, GEE, and conditional logistic regression. However, GLMMs with random treatment effect are more traditional in meta-analysis. These models may include fixed intercepts (study effects) and random treatment effect, or both intercept and treatment effect are assumed to be random [16].

In the meta-analysis of binary outcomes, the distributions of the fixed effects are based on a binomial or noncentral hypergeometric distribution, and the random effects are assumed to follow normal distribution, resulting in a binomial-normal or hypergeometric-normal likelihood, respectively. The standard REM is based on the normal approximation to the distribution of log-odds, this is the normal-normal model. For incidence rates, an example of a GLMM is the Poisson-normal model.

Turner et al. [16] introduced a mixed effects logistic regression model with random treatment effect as a multilevel model for meta-analysis of binary outcomes in a frequentist setting. Stijnen et al. [3] proposed to use a conditional logistic model with an exact noncentral hypergeometric distribution and its approximation by a binomial distribution. The difference between the standard random effects model and a mixed effects logistic regression is that the standard random effects model directly models an effect measure that reflects the contrast between the two groups (e.g., log odds ratio). The conditional logistic (hypergeometric) model deals with the OR directly as the study effects are conditioned out. The parameters in these models can be estimated by maximum likelihood or restricted maximum likelihood methods using iterative generalized least squares.

#### Standard inverse-variance random effects model for the meta-analysis of binary outcomes (REM)

Consider *K* comparative studies reporting summary binary outcomes. The data from each study *i*=1,⋯,*K* constitutes a pair of independent binomial variables *y*_*i*1_ and *y*_*i*2_, numbers of events out of *n*_*i*1_ and *n*_*i*2_ subjects for the treatment and control arms. The risks in the treatment and the control arms are denoted by *π*_*ij*_ for *j*=1,2, respectively. The log odds ratio for individual study *i* is *θ*_*i*_= log(*π*_*i*1_(1−*π*_*i*2_)/(*π*_*i*2_(1−*π*_*i*1_))).

The standard REM is a two-level model. At the first level, conditionally on the study effects *θ*_*i*_, empirical LORs $\hat {\theta }_{i}$ are assumed to be normally distributed with unknown means *θ*_*i*_ and within-study variances $\sigma _{i}^{2}$, $\hat {\theta }_{i}\sim N\left (\theta _{i},\sigma _{i}^{2}\right)$. The variances $\sigma _{i}^{2}=[n_{i1}\pi _{i1}(1-\pi _{i1})]^{-1}+[n_{i2}\pi _{i2}(1-\pi _{i2})]^{-1}$ are estimated from the data, but their estimates $\hat \sigma _{i}^{2}$ are assumed to be known. At the second level, the true within-study effects *θ*_*i*_ are assumed to have a normal distribution with mean *θ* and unknown between study variance *τ*^2^, i.e. *θ*_*i*_∼*N*(*θ*,*τ*^2^), where *θ* is the overall log odds ratio. Marginally, $\hat {\theta }_{i}\sim N\left (\theta,\sigma _{i}^{2}+\tau ^{2},\right)$ so that $\hat {\theta }_{i}=\theta +\nu _{i}+\epsilon _{i}$ with *ν*_*i*_∼*N*(0,*τ*^2^), $\epsilon _{i}\sim N\left (0,\sigma _{i}^{2}\right)$ and Cov(*ν*_*i*_,*ε*_*i*_)=0. The between-study variance *τ*^2^ is usually estimated by DL [20] or REML, and the overall LOR *θ* is estimated using the inverse variance weights $w_{i}=\left (\hat \sigma _{i}^{2}+\hat \tau ^{2}\right)^{-1}$ as $\hat {\theta }=\sum w_{i}\hat \theta _{i}/\sum w_{i}$.

#### GLMMs with fixed intercept (FIM)

GLMM with fixed intercept is a special case of mixed effects logistic regression model [16]. The model also accounts for heterogeneity between studies on the log odds scale. The model is written as:

$$y_{ij}|\pi_{ij}\sim Binomial(n_{ij},\pi_{ij})\quad j=1,2;\quad i=1,\ldots,K,$$

2 $$ \log\left(\frac{\pi_{ij}}{1-\pi_{ij}}\right)=\phi_{i}+(\theta+v_{i})x_{ij},  $$

where *π*_*ij*_ are the probabilities of an event in each arm, *θ* is the overall effect (log odds ratio), and the random effects *v*_*i*_∼*N*(0,*τ*^2^) are the deviations of the *i*^*th*^ study treatment effect (log odds ratio) from the overall effect *θ*, with *τ*^2^ being the between-study variance. The fixed intercepts *ϕ*_*i*_ are the log-odds in the control arms. The *x*_*ij*_ is the group dummy variable. When *x*_*ij*_=0/1, then model (2) can be written as:

$$\log\left(\frac{\pi_{i1}}{1-\pi_{i1}}\right)=\phi_{i}+\theta+v_{i}\quad\text{and}\quad\log\left(\frac{\pi_{i2}}{1-\pi_{i2}}\right)=\phi_{i}, $$

for the treatment and control groups, respectively, so that

3 $$ \left(\begin{array}{ccc} \log\left(\frac{\pi_{i2}}{1-\pi_{i2}}\right) \\ \log\left(\frac{\pi_{i1}}{1-\pi_{i1}}\right) \\ \end{array}\right) \sim N \left(\left(\begin{array}{ccc} \phi_{i} \\ \phi_{i}+\theta \\ \end{array}\right), \left(\begin{array}{ccc} 0 & 0 \\ 0 & \tau^{2}\\ \end{array}\right)\right).  $$

We will refer to this model as FIM1.

This model assumes higher variability in the treatment groups. In order to avoid this asymmetry, a coding of +1/2 and −1/2 was suggested for the group dummy *x*_*ij*_ in [16]. When *x*_*ij*_=±1/2 and after reparametrization $\phi ^{*}_{i}=\phi _{i}-\theta /2$, the model (2) can be written as:

$$\begin{aligned} \log\left(\frac{\pi_{i1}}{1-\pi_{i1}}\right)&=\phi_{i}^{*}+\theta+0.5v_{i}\quad\text{and}\\[-4pt]&\quad\log\left(\frac{\pi_{i2}}{1-\pi_{i2}}\right)=\phi_{i}^{*}-0.5v_{i}, \end{aligned} $$

for the treatment and control groups, so that

4 $$ \left(\begin{array}{ccc} \log\left(\frac{\pi_{i2}}{1-\pi_{i2}}\right) \\ \log\left(\frac{\pi_{i1}}{1-\pi_{i1}}\right) \\ \end{array}\right) \sim N \left(\left(\begin{array}{ccc} \phi_{i}^{*} \\ \phi_{i}^{*}+\theta \\ \end{array}\right), \left(\begin{array}{ccc} \tau^{2}/4 & -\tau^{2}/4 \\ -\tau^{2}/4 & \tau^{2}/4\\ \end{array}\right)\right).  $$

We will refer to this model as FIM2. In [21], the models FIM1 and FIM2 are referred to as models 2 and 4, respectively. They are logistic regression models with *ϕ*_*i*_= log(*π*_*i*2_/(1−*π*_*i*2_)) as the study-specific fixed intercepts that have to be estimated. The unknown parameters *ϕ*_*i*_, *θ* and *τ*^2^ are estimated iteratively using marginal quasi-likelihood, penalized quasi-likelihood, or first- and second-order Taylor-expansion approximation. In order to remove the bias of the between-study variance estimates from penalized quasi-likelihood methods, a two-step bootstrap procedure can be used [16]. Jackson et al. [21] demonstrated in simulations and provided a theoretical explanation for the inferiority of FIM1 in comparison to FIM2 in respect to considerable underestimation of the heterogeneity variance *τ*^2^. We further study FIM2 but not FIM1 in our simulations.

#### GLMMs with random intercept (RIM)

A GLMM with a random intercept is a mixed effects logistic regression model with a random intercept and random treatment effect [16]. The model can be written as:

$$y_{ij}\sim Binomial(n_{ij},\pi_{ij});\quad j=1,2,\quad i=1,\ldots,K,$$

5 $$ \log\left(\frac{\pi_{ij}}{1-\pi_{ij}}\right)=\phi+u_{i}+(\theta+v_{i})x_{ij},  $$

where *ϕ* is the baseline log-odds, *θ* is the overall effect (log-odds-ratio), the random effects are random variables from a bivariate normal distribution *v*_*i*_∼*N*(0,*τ*^2^), *u*_*i*_∼*N*(0,*σ*^2^) and Cov(*u*_*i*_,*v*_*i*_)=*ωστ*. This general bivariate normal random effects model was introduced in [17] and further discussed in [3]. When *x*_*ij*_=0/1, and assuming Cov(*u*_*i*_,*v*_*i*_)=0, the model (5) can be written as:

$$\log\left(\frac{\pi_{i1}}{1-\pi_{i1}}\right)\,=\,\phi+u_{i}+\theta+v_{i}\,\,\text{and}\,\log\left(\frac{\pi_{i2}}{1-\pi_{i2}}\right)\,=\,\phi+u_{i}, $$

so that

6 $$ \left(\begin{array}{ccc} \log\left(\frac{\pi_{i2}}{1-\pi_{i2}}\right) \\ \log\left(\frac{\pi_{i1}}{1-\pi_{i1}}\right) \\ \end{array}\right) \sim N \left(\left(\begin{array}{ccc} \phi \\ \phi+\theta \\ \end{array}\right), \left(\begin{array}{ccc} \sigma^{2} & \sigma^{2} \\ \sigma^{2} & \sigma^{2}+\tau^{2}\\ \end{array}\right)\right).  $$

We will refer to this model as RIM1.

Similarly to FIM2, when *x*_*ij*_=±1/2 and assuming Cov(*u*_*i*_,*v*_*i*_)=0, model (5) can be reparametrized as:

$$\begin{aligned} \log\left(\frac{\pi_{i1}}{1-\pi_{i1}}\right)&=\phi^{*}+u_{i}+\theta+0.5v_{i}\quad\text{and}\\[-4pt]&\quad\log\left(\frac{\pi_{i2}}{1-\pi_{i2}}\right)=\phi^{*}+u_{i}-0.5v_{i}, \end{aligned} $$

for the treatment and control groups, so that

7 $$ \begin{aligned} \left(\!\!\begin{array}{ccc} \log\left(\!\frac{\pi_{i2}}{1-\pi_{i2}}\right) \\ \log\left(\!\frac{\pi_{i1}}{1-\pi_{i1}}\right) \\ \end{array} \!\!\right)& \!\sim N\! \left(\!\! \left(\! \begin{array}{ccc} \phi^{*} \\ \phi^{*}+\theta \\ \end{array}\!\right)\right.\!, \left(\! \begin{array}{ccc} \sigma^{2}+\tau^{2}/4 & \sigma^{2}-\tau^{2}/4 \\ \sigma^{2}-\tau^{2}/4 & \sigma^{2}+\tau^{2}/4\\ \end{array}\!\right)\left.{\vphantom{\left(.\right)\! \left(\begin{array}{ccc} \phi^{*} \\ \phi^{*}+\theta \\ \end{array}\!\right)}}\!\!\! \right). \end{aligned}  $$

We will refer to this model as RIM2.

The RIM models include two or three (when *ω*=Cov(*u*_*i*_,*v*_*i*_)≠0) heterogeneity parameters (*σ*^2^, *τ*^2^, *ω*) in contrast to the standard random effects model with a single between-study variance *τ*^2^. The unknown parameters *ϕ*, *θ*, *σ*^2^, *τ*^2^ and *ω* can be estimated similarly to estimation in a GLMM with fixed study effects [16]. In [21], the models RIM1 and RIM2 are referred to as models 3 and 5, respectively, and appear to have very similar properties, whereas our general model (5) is their Model 6. The properties of a logistic regression model with a random intercept for the meta-analysis of proportions were also studied by Hamza et al. [27], and for the case of scarce data by Kuss [9]. We further study RIM2 in our simulations.

#### A GLMM with exact noncentral hypergeometric-normal likelihood (NCHGN)

The hypergeometric-normal model was initially proposed for meta-analysis by Van Houwelingen et al. [17] and Liu and Pierce [18]. Later, Stijnen et al. [3] and Sidik and Jonkman [19] implemented the model. Some simulation results are given in [21], their model 7.

The data may be generated from either FIM or RIM. Conditioning on the total number of events for study *i*, only the number of events in the treatment group *y*_*i*1_ is random. NCHGN is a two-level model. Given the study-specific log odds ratio *θ*_*i*_, the distribution of *y*_*i*1_ is the noncentral hypergeometric distribution. Next, the LORs *θ*_*i*_ are normally distributed *θ*_*i*_∼*N*(*θ*,*τ*^2^). The exact likelihood function of the hypergeometric-normal model for study *i* can be written as:

8 $$ h\left(y_{i1};\theta,\tau^{2}\right)=\int_{-\infty}^{\infty}f(y_{i1}|\theta_{i})\phi\left(\theta_{i}|\theta,\tau^{2}\right)d\theta_{i}=  $$

$$\int_{-\infty}^{\infty}{n_{i1}\choose y_{i1}}{n_{i2}\choose y_{i2}}\frac{\exp(y_{i1}\theta_{i})}{P(\theta_{i})}\frac{1}{\sqrt{2\pi\tau^{2}}}\exp\left(\!-\frac{(\theta_{i}\,-\,\theta)^{2}}{2\tau^{2}}\right)d\theta_{i},$$

*f*(*y*_*i*1_|*θ*_*i*_) is the noncentral hypergeometric probability function for the number of events in the treatment arm *Y*_*i*1_ given *Y*_*i*1_+*Y*_*i*2_=*Y*_*i*_, and the normalizing constant is defined as:

$$P(\theta_{i})=\sum_{i=\max(0,n_{i}-n_{i2})}^{\min(n_{i1},n_{i2})}{n_{i1}\choose i}{n_{i2}\choose Y_{i}-i}\exp(Y_{i}\theta_{i}).$$

The density of the distribution of log odds ratios between the studies, denoted by *ϕ*(*θ*_*i*_|*θ*,*τ*^2^), is normal with mean *θ* and variance *τ*^2^. The density *h*(*y*_*i*1_|*θ*,*τ*^2^) is the density of the marginal distribution after integrating out unobserved study-specific effects. When *f*(·) is a noncentral hypergeometric and *ϕ*(·) is a normal density, the model is referred to as a hypergeometric-normal model [3]. According to Stijnen et al. [3], this approach should solve issues related to the adjustments to zero cells and the existence of correlation between $\hat {\sigma }_{i}^{2}$ and $\hat {\theta }_{i}$ in the standard random effects model. This model is a mixed effects logistic model. Liang [28] have shown that inferences based on the noncentral hypergeometric likelihood are sensitive to misspecification of the dependence structure, see also [18] for approximations to *h*(*y*_*i*1_;*θ*,*τ*^2^) and [22] for the full likelihood analysis for generalized linear mixed models such as the penalized quasi-likelihood and marginal quasi-likelihood methods.

The unknown parameters *θ* and *τ*^2^ can be estimated by using the EM algorithm [17] or the numerical Newton-Raphson iterative algorithm [19], or by maximizing log-likelihood of NCHGN [3, 29]. Liu and Pierce [18] approximated the integrand by a mixture of noncentral hypergeometric and normal densities based on Laplace’s method. However, the most recent approximations for the marginal likelihood of noncentral hypergeometric-normal distribution are based on adaptive Gauss-Hermite quadrature. The noncentral hypergeometric distribution is based on the binomial distributions in the treatment and control arms. When that assumption is invalid, *y*_*i*1_ no longer follows a noncentral hypergeometric distribution [30].

#### A GLMM with an approximate binomial-normal likelihood (ABNM)

For small total numbers of events relative to the total group sizes, the noncentral hypergeometric distribution can be approximated by a binomial distribution [3]:

$$y_{i1}|(y_{i1}+y_{i2})\sim Binomial\left(y_{i1}+y_{i2},P_{y_{i1}|(y_{i1}+y_{i2})}\right)$$

with

9 $$ \begin{aligned} \log\left(\frac{P_{y_{i1}|(y_{i1}+y_{i2})}}{1-P_{y_{i1}|(y_{i1}+y_{i2})}}\right)&=\log\left(\frac{n_{i1}}{n_{i2}}\right)+\theta_{i}\qquad\text{and}\\& \theta_{i}\sim N\left(\theta,\tau^{2}\right), \end{aligned}  $$

where $P_{y_{i1}|(y_{i1}+y_{i2})}$ is the probability of events *y*_*i*1_ conditioned on assumption of binomial distribution with the total sample sizes *y*_*i*1_+*y*_*i*2_. This approximation holds because the sample odds ratio can be rewritten via

$$\exp(\hat{\theta}_{i})=\frac{y_{i1}(n_{i2}-y_{i2})}{y_{i2}(n_{i1}-y_{i1})}=\frac{\hat{P}_{y_{i1}|(y_{i1}+y_{i2})}}{1-\hat{P}_{y_{i1}|(y_{i1}+y_{i2})}}\frac{(n_{i2}-y_{i2})}{(n_{i1}-y_{i1})}.$$

If *y*_*i*1_ and *y*_*i*2_ are small relative to *n*_*i*1_ and *n*_*i*2_, then

$$\frac{(n_{i2}-y_{i2})}{(n_{i1}-y_{i1})}\approx\frac{n_{i2}}{n_{i1}}.$$

Thus,

$$\exp(\hat{\theta}_{i})\,=\,\frac{\hat{P}_{y_{i1}|(y_{i1}+y_{i2})}}{1-\hat{P}_{y_{i1}|(y_{i1}+y_{i2})}}\frac{(n_{i2}-y_{i2})}{(n_{i1}-y_{i1})}\!\approx\!\frac{\hat{P}_{y_{i1}|(y_{i1}+y_{i2})}}{1-\hat{P}_{y_{i1}|(y_{i1}+y_{i2})}}\frac{n_{i2}}{n_{i1}}$$

and

$$\begin{aligned} \hat{\theta}_{i}&=\log\left(\frac{\hat{P}_{y_{i1}|(y_{i1}+y_{i2})}}{1-\hat{P}_{y_{i1}|(y_{i1}+y_{i2})}}\right)+\log\left(\frac{n_{i2}}{n_{i1}}\right)\\&\quad= \log\left(\frac{\hat{P}_{y_{i1}|(y_{i1}+y_{i2})}}{1-\hat{P}_{y_{i1}|(y_{i1}+y_{i2})}}\right)-\log\left(\frac{n_{i1}}{n_{i2}}\right). \end{aligned} $$

The parameters of this model can be estimated by maximizing a logistic regression model with a random intercept and offset log(*n*_*i*1_/*n*_*i*2_).

### Fitting the GLMMs for log odds in metafor

Procedure rma.glmm in the R package *metafor* can be used to fit four of the models discussed in this section: FIM2, RIM2, NCHGN and ABNM (R code is given in Additional file 1). To avoid the problem of having lower variance in the control group than in the treatment group, *metafor* uses the coding +1/2 and −1/2 for the group indicator. Viechtbauer [7] and Turner et al. [16] provide more details. GLMMs with fixed and random intercepts are fitted by specifying the options model =“UM.FS” and model =“UM.RS”, respectively.

The noncentral hypergeometric-normal model proposed by Stijnen et al. [3] is fitted by specifying the option model =“CM.EL”. R provides two methods for obtaining the probability mass function of the noncentral hypergeometric distribution: “dFNCHypergeo” in the *BiasedUrn* package [31] and “dnoncenhypergeom” in the *MCMCpack* package [32]. Both methods can be used with the rma.glmm function of *metafor*. The “dFNCHypergeo” is the default distribution in rma.glmm for fitting the NCHGN model, but “dnoncenhypergeom” can also be specified. The two methods should perform similarly, however, switching to “dnoncenhypergeom” may help to resolve the convergence problems which might occur when trying to fit a saturated model.

rma.glmm also allows a choice of an optimization method for fitting a fixed effects or a saturated model when the option model =“CM.EL” is specified. The general-purpose optimization algorithms include the default quasi-Newton method (option “BFGS”) implemented in the “optim” function, or the choice of “nlminb” function using the PORT library, [33], both in *stats* package. Alternatively, derivative-free optimization algorithms using quadratic approximation routines due to Powell [34] are available in the functions “bobyqa”, “newuoa”, or “uobyqa” from *minqua2* package. We studied both specifications of noncentral hypergeometric probability mass function and all five optimizers in our simulations.

We also studied the performance of the ABNM which uses the binomial-normal approximation to the hypergeometric distribution and therefore is less computer-intensive. This model is specified as the option model =“CM.AL” in rma.glmm. More details are given in [7, 16].

### Simulation study

We carried out a simulation study to assess the performance of the point and interval estimators of the overall log odds ratio *θ* and the between-study variance *τ*^2^ for binary outcomes generated from a REM. The estimators of *θ* and *τ*^2^ are obtained from the four generalized linear mixed models FIM2, RIM2, NCHGN and ABNM. We also included the estimates from the REM using the DL [20] and the restricted maximum likelihood methods for comparison.

We generated the data as follows:

$$y_{i1}\sim Binom(n_{i1},f(p_{i2},\theta_{i}))\quad\text{and}\quad y_{i2}\sim Binom(n_{i2},p_{i2}),$$

where *θ*_*i*_∼*N*(*θ*,*τ*^2^) and *f*(*p*_*i*2_,*θ*_*i*_)=*p*_*i*2_ exp(*θ*_*i*_)/(1−*p*_*i*2_+ exp(*θ*_*i*_)*p*_*i*2_)). This scenario is similar to the approach in [35]. No continuity corrections are added to the numbers of events. The studies with *y*_*i*1_=0 and *y*_*i*2_=0 or *y*_*i*1_=*n*_*i*1_ and *y*_*i*2_=*n*_*i*2_ were omitted from the modelling.

The sample sizes are assumed to be the same within the two arms and across all *K* studies. Procedure rma.glmm from *metafor* version 1.9-2 with the default control parameters was used to fit the GLMM models, unless stated otherwise.

For the simulations where the convergence was achieved, we assessed the bias of the maximum likelihood estimators of *τ*^2^ and *θ* and the coverage of the 95% confidence intervals for *θ*. The default normal critical values were used for the confidence intervals.

We used the University of East Anglia 334 node High Performance Computing (HPC) Cluster, providing a total of 4784 cores, including parallel processing and large memory resources. For each configuration, our simulations were subdivided into 100 parallel parts with 100 replications in each part, resulting in 10,000 replications in total. The total time per combination of a value of the baseline risk *p*_*i*2_ and a value of *θ*, was approximately 120 hours.

### Configurations

The simulations used the following configurations of the parameters. The number of studies was *K*=(3,5,10,30); the sample sizes in each arm across *K* studies were *n*=(50,100,250,1000); the between-study variance was *τ*^2^=(0,0.1,0.2,0.3,0.4,0.5,0.6,0.7,0.8,0.9,1). The values of the LOR *θ* were either 0 or 1. The probability in the control group was *p*_*i*2_=(0.1, 0.2,0.4) (only *p*_*i*2_=0.1 value was studied for *K*=3.). The resulting probabilities in the treatment group are given in Table 1. A total of 10,000 repetitions were produced for each configuration. However, not all the simulations converged due to problems of fitting the saturated model, and the actual number of repetitions may be much smaller, “Computational issues” section. The denominators were then adjusted accordingly. The probability in control group *p*_*i*2_=0.1 was of primary interest, since we were mostly interested in sparse data. The results for *p*_*i*2_=0.2 and 0.4 are given in the Additional file 2.

**Table 1:** Probabilities in the Control (*p*_*i*2_) and the Treatment arm (*p*_*i*1_) used in simulations

C\T	*θ*=0	*θ*=1
*p*_*i*2_	*p*_*i*1_	*p*_*i*1_
0.1	0.1	0.232
0.2	0.2	0.405
0.4	0.4	0.644

## Results

We generated 10,000 repetitions at each configuration of the parameters using the default optimizer “optim” to fit the GLMMs. The results of the bias and coverage of the parameters when using this optimizer are reported in “Simulation results for default settings” section for *K*≥5. Additional results for *K*=3 are reported and discussed in Additional file 3. The convergence of this and alternative optimizers, “nlminb”, “bobyqa”, “newuoa”, and “uobyqa” are reported in “Computational issues” section. The results for the bias and coverage of the parameters when using these alternative optimizers are considered in “Simulation results for alternative optimizers” section. An example is given in “Example: effects of diuretics on pre-eclampsia” section.

### Simulation results for default settings

**Fig. 1:**
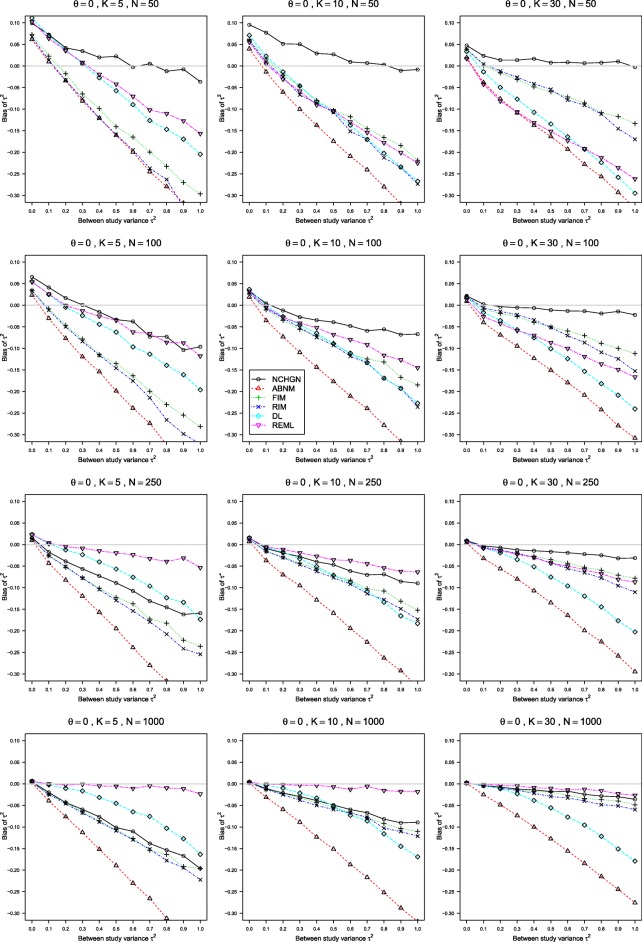
Bias of *τ*^2^ in the REM when *p*_*i*2_=0.1, *θ*=0, 0≤*τ*^2^≤1 and *n*=50,100,250,1000. Estimation methods are: pluses - unconditional generalized linear mixed-effects model with fixed study effects (FIM), crosses - unconditional generalized linear mixed-effects model with random study effects (RIM), circles - a conditional generalized linear mixed-effects model with exact likelihood (NCHGN), triangles - a conditional generalized linear mixed-effects model with approximate likelihood (ABNM), rhombs - DerSimonian and Laird method (DL) and reverse triangles - restricted maximum likelihood method (REML). Light grey line at 0

**Fig. 2:**
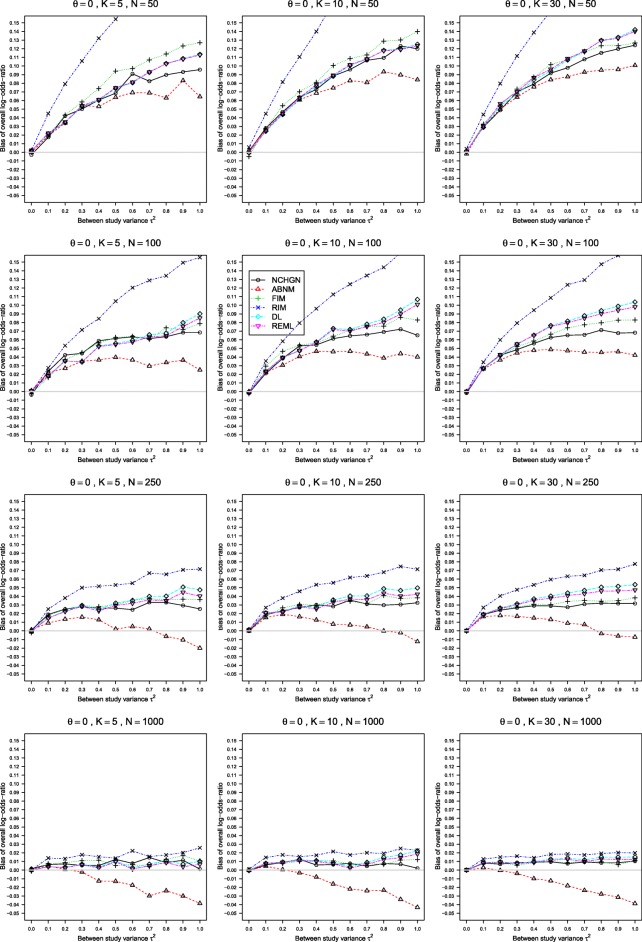
Bias of *θ* in the REM when *p*_*i*2_=0.1, *θ*=0, 0≤*τ*^2^≤1 and *n*=50,100,250,1000. Estimation methods are: pluses - unconditional generalized linear mixed-effects model with fixed study effects (FIM), crosses - unconditional generalized linear mixed-effects model with random study effects (RIM), circles - a conditional generalized linear mixed-effects model with exact likelihood (NCHGN), triangles - a conditional generalized linear mixed-effects model with approximate likelihood (ABNM), rhombs - DerSimonian and Laird method (DL) and reverse triangles - restricted maximum likelihood method (REML). Light grey line at 0

**Fig. 3:**
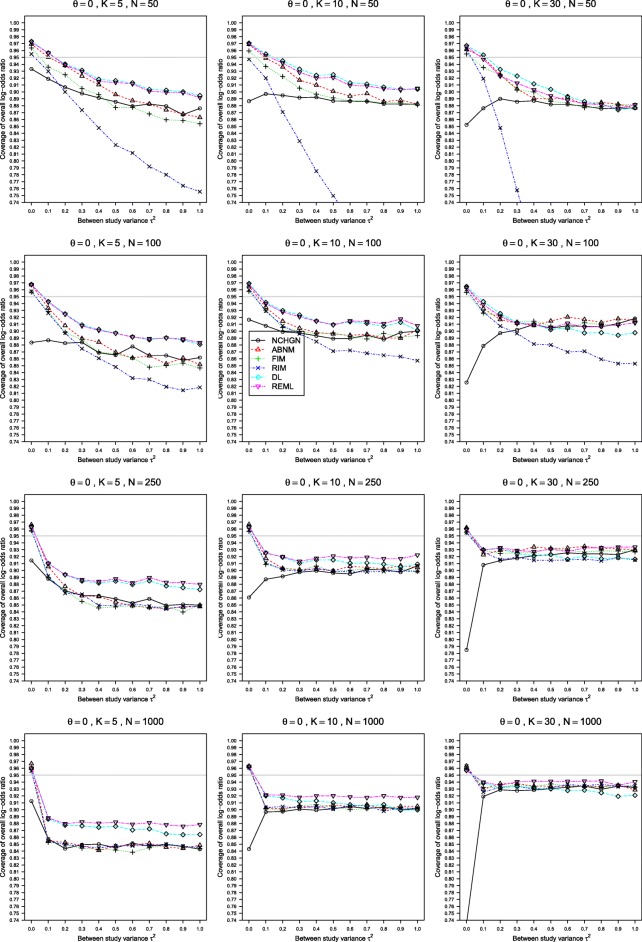
Estimated coverage of *θ* in the REM when *p*_*i*2_=0.1, *θ*=0, 0≤*τ*^2^≤1 and *n*=50,100,250,1000. The coverages are given at the nominal 95% level. Estimation methods are: pluses - unconditional generalized linear mixed-effects model with fixed study effects (FIM), crosses - unconditional generalized linear mixed-effects model with random study effects (RIM), circles - a conditional generalized linear mixed-effects model with exact likelihood (NCHGN), triangles - a conditional generalized linear mixed-effects model with approximate likelihood (ABNM), rhombs - DerSimonian and Laird method (DL) and reverse triangles - restricted maximum likelihood method (REML). Light grey line at 0.95

The results of our simulations for various values of *K*≥5 and *n* are given in Figs. 1, 2 and 3 for the true LOR *θ*=0, *p*_*i*2_=0.1, and 0≤*τ*^2^≤1. This scenario produces sparse data in the treatment and control arms. The results for *θ*=1, *p*_*i*2_=0.1 and for higher probabilities *p*_*i*2_=0.2 and *p*_*i*2_=0.4 are shown in Figure A4 - Figure A18 in the Additional file 2. The results were very similar for the GLMM with exact noncentral hypergeometric-normal likelihood (NCHGN method) regardless of the used programme for hypergeometric distribution, see Figure A1 - Figure A3 in the Additional file 4. Only the results with the default dFNCHypergeo option are shown in Figs. 1, 2 and 3 for the NCHGN method. The default optimizer “optim” was used throughout this Section unless stated otherwise.

**Fig. 4:**
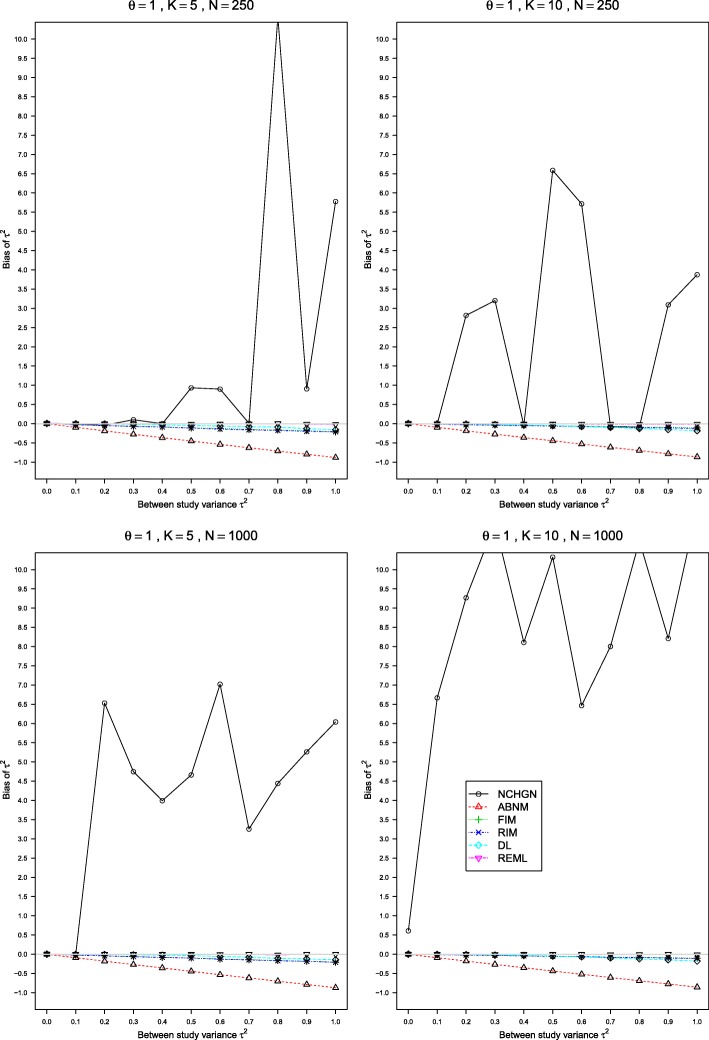
Bias of *τ*^2^ in the REM when *p*_*i*2_=0.4, *θ*=1, 0≤*τ*^2^≤1 and *n*=50,100,250,1000. Estimation methods are: pluses - unconditional generalized linear mixed-effects model with fixed study effects (FIM), crosses - unconditional generalized linear mixed-effects model with random study effects (RIM), circles - a conditional generalized linear mixed-effects model with exact likelihood (NCHGN), triangles - a conditional generalized linear mixed-effects model with approximate likelihood (ABNM), rhombs - DerSimonian and Laird method (DL) and reverse triangles - restricted maximum likelihood method (REML). Light grey line at 0.95

For all methods, the bias in the estimation of *τ*^2^ (Fig. 1 and Figure A4, Figure A7, Figure A8, Figure A13, Figure A14 in the Additional file 2), is almost linear over the range of *τ*^2^, *K* and *n*. The bias is positive for smaller values of *τ*^2^, where the GLMM with exact noncentral hypergeometric-normal likelihood (NCHGN method) provides the highest values when *n*≤100, but otherwise is negative. The results for smaller sample sizes (*n*≤100) differ from those for larger values of *n*≥250, where the REML performs the best across the board, and always better than the DL [20] method. The bias of the DL method is especially pronounced when *K*≥10. For smaller sample sizes, the two main contenders for the best estimation of *τ*^2^ are the exact NCHGN method and the REML. The REML is always the best choice when *K*=5 but for the case of *p*_*i*2_=0.1, *θ*=0, *n*=50, where the NCHGN is better for large *τ*^2^. Similarly, when *K*=10, the NCHGN method is better than the REML for larger *τ*^2^ and smaller *n* values when both probabilities are small. The NCHGN method is always a good choice when *K*=30, and is the best for sparse data. However, the REML is better for larger probabilities, see Additional file 2: Figure A13 and Figure A14, and the NCHGN behaves erratically for large sample sizes, as can be seen in Fig. 4 and is discussed in more detail in “Computational issues” section. Bias of all the other methods generally decreases with larger *n* and with larger *K*, but for the GLMM with approximate binomial-normal likelihood (ABNM), which performs the worst and appears to be asymptotically biased.

In respect to the estimation of the overall LOR $\hat \theta $, all methods perform well for larger probabilities (from 0.4) in at least one arm, Additional file 2: Figure A10 - Figure A16, although the NCHGN behaves erratically for *n*=1000, Additional file 2: Figure A16. The distinctions are clear only for relatively small probabilities in both arms, Fig. 2, Additional file 2: Figure A5 and Figure A9. The estimates of the overall LOR $\hat \theta $ are mostly considerably positively biased. The only exceptions are the DL and the REML based inverse variance methods for small *τ*^2^, and the conditional GLMM with approximate binomial-normal likelihood (ABNM) which often has large negative bias. Overall, the ABNM has the lowest values of $\hat \theta $, which is an unexpected advantage for sample sizes up to 250 when *p*_*i*2_≤0.2, where the conditional GLMM with exact likelihood, NCHGN, provides the second lowest but still positively biased, values of $\hat \theta $. The GLMM model with random intercept, RIM, has the largest positive bias. Bias increases with larger *τ*^2^, and may be considerable for large values of *τ*^2^ and moderate *n* when *p*_*i*2_≤0.2. For relatively sparse data and large values of *τ*^2^, the NCHGN performs somewhat better than the standard methods DL and REML, which are very similar to each other. Overall, the biases of the LOR $\hat {\theta }$ are smaller when *p*_*iC*_>0.1 in comparison to the case of sparse data in both arms.

The coverage of *θ*, Fig. 3 and Figure A6, Figure A11, Figure A12, Figure A17, Figure A18 in the Additional file 2, is closely related to the bias of its estimation. The coverage is typically lower than nominal, always for the NCHGN, and for all but the smallest values of *τ*^2^, below 0.1 or even lower when *n* is large, for all the other methods. The RIM has exceptionally low coverage for sparse data. The coverage is strikingly better when *θ*=1, where it is above 90% for all methods except the NCHGN, but it is unacceptably low when *θ*=0 where it deteriorates for all methods but the NCHGN with increasing *τ*^2^. The NCHGN demonstrated the worst coverage at low values of *τ*^2^, and a relatively stable, but still too low, coverage under large heterogeneity. The coverage is very low, even for large sample sizes, when the number of trials *K*=5 and improves for larger values of *K*, where increase in sample sizes also improves coverage. The standard REML and DL perform equally or somewhat better than all the GLMM methods in all possible scenarios.

### Computational issues

**Fig. 5:**
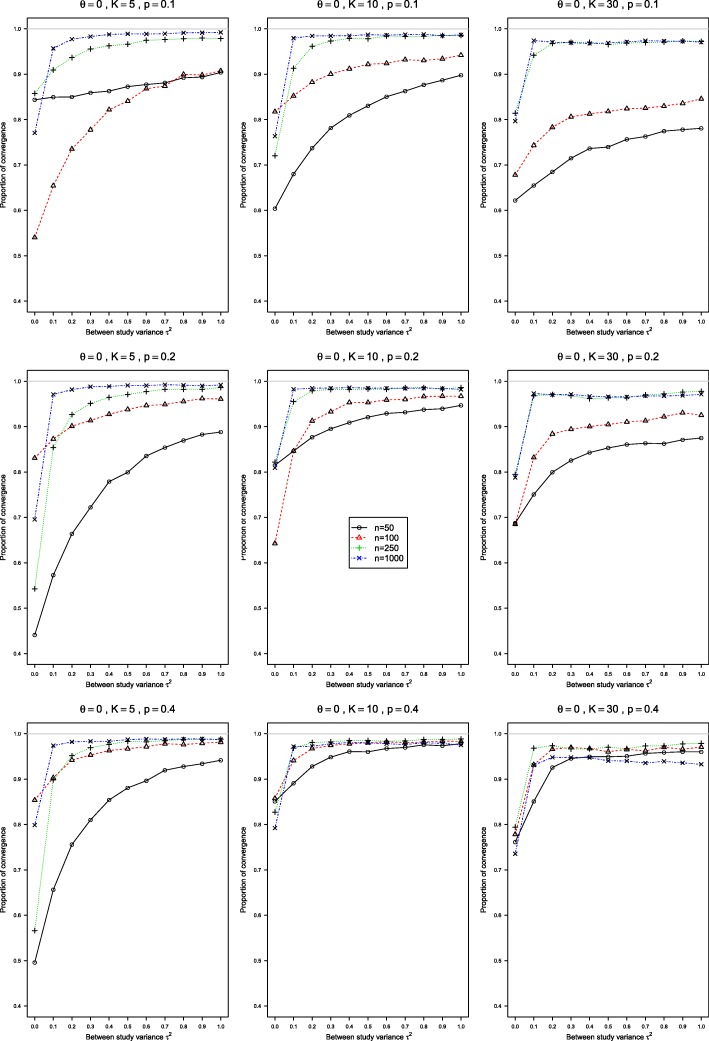
Proportion of convergence in the conditional generalized linear mixed-effects model with exact likelihood. These proportions of convergence are for *p*_*i*2_=0.1, *p*_*i*2_=0.2, *p*_*i*2_=0.4, *θ*=0, and 0≤*τ*^2^≤1 for sample sizes *n*=50,100,250,1000 in each arm

**Fig. 6:**
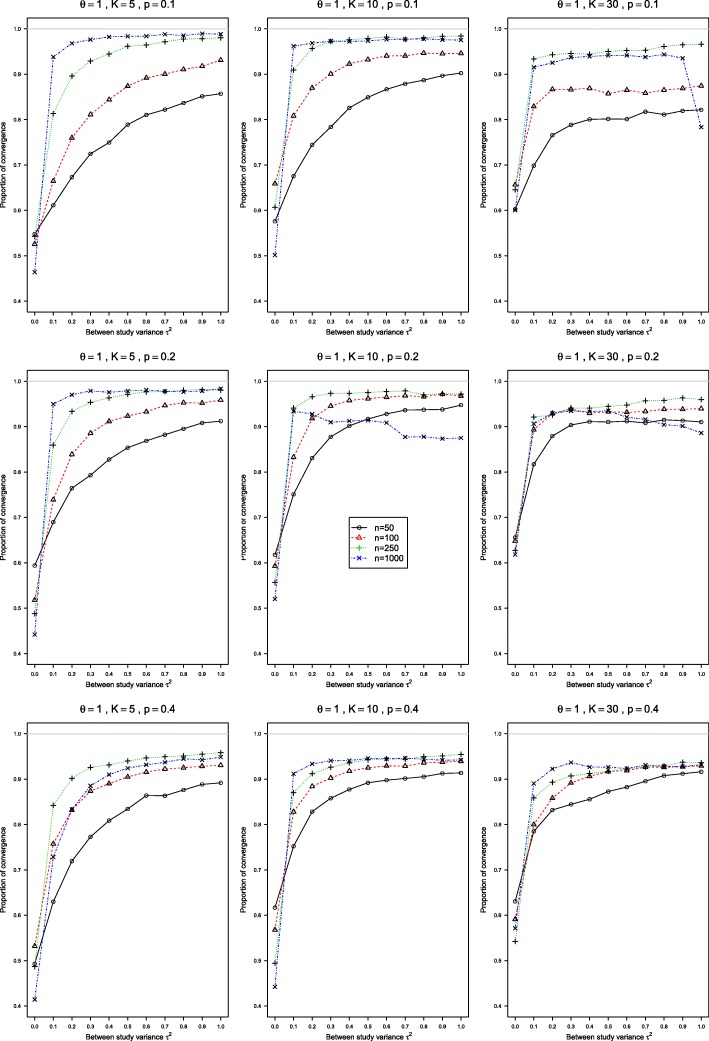
Proportion of convergence in the conditional generalized linear mixed-effects model with exact likelihood. These proportions of convergence are for *p*_*i*2_=0.1, *p*_*i*2_=0.2, *p*_*i*2_=0.4, *θ*=1, and 0≤*τ*^2^≤1 for sample sizes *n*=50,100,250,1000 in each arm

The convergence rates of the conditional GLMM with exact noncentral hypergeometric-normal likelihood (NCHGN) and the random intercept GLMM (RIM) methods implemented in the procedure rma.glmm in *metafor* were rather low, see Figs. 5 and 6 for the NCHGN, and Figure A19 and Figure A20 in the Additional file 5 for the RIM method. For the NCHGN method, the convergence is the lowest at *τ*^2^=0, where it can be as low as 40%, whereas for the RIM it is the lowest at *τ*^2^=1. For both methods, the convergence is the worst for small probabilities, and improves for large sample sizes.

Another important computational issue is the nonstable performance of the NCHGN for large sample sizes when the default “optim” optimizer is used. Some datasets result in anomalously large estimated values of *τ*^2^ and, consequently, *θ*. This behavior is illustrated by Fig. 4 (this is a blow-out of Figure A14 in the Additional file 2).

We provide an example of a simulated dataset causing this problematic behaviour in Table 2. The results of the NCHGN with all the available optimizers in rma.glmm and also of the standard REM methods are provided in Table 3 and R code is given in Additional file 6. All the GLMMs except the ABNM and NCHGN with “optim” result in very similar estimates of $\hat \tau ^{2}=0.31$, and the LOR $\hat \theta \approx 1.55$. The standard REM methods provide similar values. However, the NCHGN used with “optim” results in $\hat \tau ^{2}=1169.06$ and $\hat \theta = 35.68$. Such high values of biases even for one observation would considerably increase the mean biases as can be seen in Fig. 4. The reason for this has to do with a bug in “optim” as the other optimizers provide consistent results. We also tried to reduce sample sizes in this simulated data by considering datasets with all the values of *y*_*ji*_ and *n*_*ji*_ reduced by a factor of *a* (and taking an integer part if needed), for *a*=1.1,1.2,2,3,4,5,6,9,10. For all these smaller datasets, the NCHGN method with the “optim” optimizer either has not converged (for *a*=2, 6, 8 and 10), or resulted in consistent estimates. We also tested other available in rma.glmm optimizers on these smaller datasets. They all converge every time, although the optimizer “uobyqa” provides very different estimates of *τ*^2^ and *θ* when *a*=6.

**Table 2:** Simulated data from REM with *p*_*iC*_=0.4, *θ*=1, *τ*^2^=0.6, *K*=5 and *n*_1*i*_=*n*_2*i*_=1000

	*y*_1 *i*_	*n*_1*i*_−*y*_1*i*_	*n*_1 *i*_	*p*_1 *i*_	*y*_2 *i*_	*n*_2*i*_−*y*_2*i*_	*n*_2 *i*_	*p*_2 *i*_	*θ*	*OR*
1	726	274	1000	0.726	401	599	1000	0.401	1.376	3.958
2	741	259	1000	0.741	406	594	1000	0.406	1.432	4.186
3	892	108	1000	0.892	378	622	1000	0.378	2.609	13.591
4	630	370	1000	0.63	415	585	1000	0.415	0.876	2.400
5	745	255	1000	0.745	404	596	1000	0.404	1.461	4.310

**Table 3:** Meta-analysis of simulated data

Model	Method	Optimizer	Hetero	LOR	*L*	*U*	Length	OR
			geneity				of CI	
GLMM	FIM		0.3106	1.5477	1.0513	2.0442	0.9929	4.700646
GLMM	RIM		0.3021	1.5446	1.0548	2.0344	0.9796	4.686097
GLMM	NCHGN	“optim”	1169.0647	35.6833	26.9652	44.4014	17.4362	3.14∗10^15^
GLMM	NCHGN	“nlminb”	0.3113	1.5472	1.0502	2.0442	0.994	4.698297
GLMM	NCHGN	“bobyqa”	0.3113	1.5472	1.0502	2.0442	0.994	4.698297
GLMM	NCHGN	“newuoa”	0.3113	1.5472	1.0502	2.0442	0.994	4.698297
GLMM	NCHGN	“uobyqa”	0.3113	1.5472	1.0502	2.0442	0.994	4.698297
GLMM	ABNM		0.0160	0.6177	0.4943	0.7411	0.2468	1.854657
FEM				1.4540	1.3671	1.5409	0.1738	4.280201
REM	DL		0.3159	1.5469	1.0463	2.0474	1.0011	4.696887
REM	REML		0.3921	1.5476	0.9916	2.1035	1.1119	4.700176

Estimates and confidence intervals (CIs) for the heterogeneity parameter *τ*^2^, for the overall log-odds-ratio (LOR) and for the overall odds ratios (OR); GLMM is the generalized linear mixed model, REM is the random-effects model and FEM is the fixed-effect model. *L* and *U* are the lower and upper limits of the respective 95% confidence intervals

To check whether the results of our simulations are affected by the use of the default optimizer “optim”, we performed additional simulations (1000 repetitions per configuration) for the problematic combination of *p*_*i*2_=0.4, *θ*=1, and also for *p*_*i*2_=0.1, *θ*=1 for *K*=5 and 10 and *τ*^2^∈[0,1] using all the other available optimizers. However, we discovered that the optimizer “uobyqa” just hangs when the other optimizers report non-convergence, and we did not obtain further results from it. See the Additional file 7 for an example.

For the first combination of parameters, *p*_*i*2_=0.4, *θ*=1, the results on the convergence are summarised in Additional file 5: Figure A21, and for *p*_*i*2_=0.1, *θ*=1 in Additional file 5: Figure A25. Results on the convergence are similar for both configurations. The convergence is always the worst at *τ*^2^=0 and slowly improves for higher *τ*^2^ and for larger sample sizes. The convergence rates of the “nlminb” are similar to that of the “optim”, about 40% at zero, but the “bobyqa” and “newbyqa” converge considerably more often, with 60 to 70% rates at zero. We report the results on the bias of *τ*^2^, and the bias and coverage of *θ* for these two configurations when the alternative optimizers are used in the next Section.

### Simulation results for alternative optimizers

This Section summarises the results for the alternative optimizers when *K*=5 and *K*=10. For *p*_*i*2_=0.4, *θ*=1 the results on the bias of *τ*^2^ and *θ*, and on the coverage of *θ* are summarised in Figure A22 - Figure A24 in the Additional file 5. When the sample sizes are 50 or 100, the “optim” behaves similarly to all the other optimizers in respect to the bias of the estimation of *τ*^2^, but only this optimizer is unstable for larger sample sizes. For all the other optimizers, the bias of the estimation of *τ*^2^ is very similar, and does not much depend on the sample size *n*. The same is mostly true for the estimation of *θ*, although the “nlminb” is not stable at *τ*^2^=0 for *n*=1000.

However, the results of the coverage of *θ*, Additional file 5: Figure A24, are strikingly different from those obtained when using the “optim” (Additional file 2: Figure A18). The coverage is approximately 85% with the “optim”, but is considerably lower, especially for *τ*^2^ near zero, for all the other optimizers. Examining the individual simulated datasets, we discovered that often, even when the NCHGN converges, the output includes reasonable estimates of *τ*^2^ and *θ*, but anomalously provides low values of the standard error of *θ*, and therefore extremely narrow confidence intervals. This finding is also discussed by [21].

The results for *p*_*i*2_=0.1, *θ*=1 are provided in Additional file 5: Figure A26 - Figure A28. The bias in the estimation of *τ*^2^ is somewhat improved for large sample sizes by the “newbyqa”, but both the “bobyqa” and “nlminb” are worse at small *n* and small *τ*^2^ values. The estimation of *θ* using all the optimizers results in somewhat higher biases for small *n*. Once more, the confidence intervals for *θ* have very low coverage for small values of *τ*^2^.

We, therefore, believe that the results of the NCHGN in respect to the bias of the estimation of *τ*^2^ and *θ* for *n*≤100 are not considerably affected by the choice of the optimizer. The same is true for the results for larger sample sizes whenever the “optim” behaves consistently. The “optim” also appears to be the best optimizer when *τ*^2^ is low. The coverage of *θ* is the best with the “optim”. Overall, we agree with the choice of the “optim” as the default optimizer.

### Example: effects of diuretics on pre-eclampsia

Data from nine trials that reported the effect of diuretics on pre-eclampsia [36] were studied by Hardy and Thompson [37], Biggerstaff and Tweedie [38], Turner et al. [16], Viechtbauer [35], Kulinskaya and Olkin [39], and Bakbergenuly and Kulinskaya [10].

The data are shown in Table 4 and were re-analysed here in order to compare the results from the four GLMM models and additionally, the standard fixed effect and random effects models with inverse-variance weights. Except for the studies 3, 4 and 9, the incidence of pre-eclampsia in both arms is below 0.15. The results are shown in Table 5. The first two models are the GLMMs with fixed and random study effects given by (2) and (5), respectively. The second two models are the conditional GLMMs with exact and approximate likelihood given by (8) and (9). Both the DL [20] and REML estimation results are provided for the REM.

**Table 4:** Data for meta-analysis on effects of diuretics on pre-eclampsia, [36]

study	*y*_*i*1_	*y*_*i*2_	*n*_*i*1_	*n*_*i*2_	*p*_*i*1_	*p*_*i*2_
1	14	14	131	136	0.1068	0.1029
2	21	17	385	134	0.0545	0.1268
3	14	24	57	48	0.2456	0.5000
4	6	18	38	40	0.1579	0.4500
5	12	35	1011	760	0.0118	0.0460
6	138	175	1370	1336	0.1007	0.1310
7	15	20	506	524	0.0296	0.0382
8	6	2	108	103	0.0555	0.0194
9	65	40	153	102	0.4248	0.3921

**Table 5:** Meta-analysis of diuretics in pre-eclampsia

Model	Method	Hetero	*L*	*U*	LOR	*L*	*U*	Length	OR	*L*	*U*
		geneity						of CI			
GLMM	FIM	0.254			-0.513	-0.923	-0.104	0.819	0.599	0.398	0.901
GLMM	RIM	0.264			-0.516	-0.930	-0.102	0.828	0.597	0.395	0.903
GLMM	NCHGN	0.260	−0.147(0)	0.667	-0.513	-0.927	-0.100	0.827	0.599	0.396	0.905
GLMM	ABNM	0.165			-0.434	-0.777	-0.091	0.686	0.648	0.460	0.913
FEM					-0.398	-0.573	-0.223	0.530	0.672	0.564	0.800
REM	DL	0.230	0.072	2.202	-0.517	-0.916	-0.117	0.799	0.596	0.400	0.889
REM	REML	0.300	0.043	1.475	-0.518	-0.956	-0.080	0.876	0.596	0.384	0.923

Estimates and confidence intervals (CIs) for the heterogeneity parameter *τ*^2^, for the overall log-odds-ratio (LOR) and for the overall odds ratios (OR); GLMM is the generalized linear mixed model, REM is the random-effects model and FEM is the fixed-effect model. *L* and *U* are the lower and upper limits of the respective 95% confidence intervals

The first three GLMMs give very similar estimates of the between-study variance *τ*^2^, varying from 0.254 to 0.264. The GLMM with approximate likelihood (ABNM) resulted in a noticeably lower value, 0.165. The standard REM results in 0.230 for the DerSimonian-Laird (DL), and 0.300 for the REML estimate of *τ*^2^, respectively. The use of the REML in the REM was recommended by Viechtbauer [40] as the least biased and the most efficient estimate of *τ*^2^. However, Turner et al. [16] analysed the current example and showed that $\hat {\tau }_{REML}^{2}$ is biased downward. We agree with their view and believe that all these estimates of *τ*^2^ are too low, on the basis of the results of our simulations.

For the estimation of the LOR, the first three GLMMs give very similar estimates, −0.513 and −0.516, and these estimates are very close to those from the REM, −0.517 and −0.518. Once more, the estimate from the conditional GLMM with approximate likelihood is very different, −0.434. However, this estimate may well be very close to the true value. In our simulations, this model provided a consistently lower estimate of the LOR than the three other GLMMs, and for the similar sample sizes (an average arm size 386) and heterogeneity of approximately 0.25 in this example, the ABNM was almost unbiased in the estimation of the LOR. The widths of the confidence intervals for the LOR correspond to the estimated *τ*^2^ values; the REM with the REML has the widest confidence interval, followed by the GLMM with random study effects (RIM) and the conditional GLMM with exact likelihood (NCHGN). The approximate ABNM model gives the narrowest confidence interval, however, our simulations suggest that it may well have the best coverage when *θ*=0 and the worst coverage when *θ*≠0.

## Discussion

We examined by simulation the performance of generalized linear mixed models with exact and approximate likelihood, when applied to the meta-analysis of log odds ratios. The models were applied to data simulated from a binomial-normal model; that is, from a pair of binomial distributions within each study, with the logarithm of odds ratio normally distributed across studies.

When the sample sizes are small and binary outcomes are sparse, it is well known that the standard methods of meta-analysis have considerable bias in the estimation of both *τ*^2^ and *θ*. This is also demonstrated in our simulations. According to Stijnen et al. [3], the generalized linear mixed models were supposed to resolve this issue. In particular, a conditional generalized linear mixed model with an exact noncentral hypergeometric-normal likelihood was suggested as an alternative to the standard random effects model. Our simulations showed that the standard REML-based estimation works well for large studies (from *n*=250) and/or large event probabilities, but the NCHGN method provides considerably less biased estimation of the heterogeneity variance *τ*^2^ than all the other methods, including the DL and the REML methods, when the data are sparse, the sample sizes are small, and especially so for the large number of studies or for moderate to large values of *τ*^2^. However, our simulations demonstrated that the estimates of the LOR *θ* are considerably positively biased for all the studied methods, including the conditional GLMM with an exact noncentral hypergeometric likelihood, when *θ*=0. These biases, combined with the underestimation of the standard error of *θ* by the NCHGN and ABNM, resulted in coverage lower than the nominal confidence level of 0.95 for *θ*. We did not study the coverage of wider confidence intervals based on *t* critical values, as these intervals would still provide lower than nominal coverage due to aforementioned biases. One of the limitation of the conditional GLMM with approximate likelihood is that the assumption of small total numbers of events relative to the total group sizes is too strong and rare in real data meta-analysis of binary outcomes. In our simulations, this method performed considerably worse than the exact method for the estimation of *τ*^2^, and we do not recommend it. The two other models, with the fixed and the random study effects, were somewhere between the two conditional methods, although the random intercept model resulted in the largest positive bias for *θ*, and therefore cannot be recommended. The REML method performed the best in respect to the coverage of the log odds ratio *θ*.

The R package *metafor* can use either of two methods for fitting the conditional generalized linear mixed model with exact likelihood. The default method uses the density function dFNCHypergeo from the *BiasedUrn* package. The second method uses the density function dnoncenhypergeom from the *MCMCpack* package. The stability and performance of the two methods are similar. There are computational issues to do with the default optimizer “optim” used in the NCHGN method when the sample sizes are large, especially when the between-studies variance *τ*^2^ is considerable. However, the other optimizers are also dogged by computational issues, and overall perform worse.It would be certainly of interest to repeat our simulations using SAS.

## Conclusions

To summarise, even though there is no uniformly best method for estimating the between-study variance and overall effect measure, we recommend using the REML for the point and interval estimation of *θ*, whereas the NCHGN may be used for the estimation of *τ*^2^ when the sample sizes are small and the data are sparse. When the sample sizes are large, we recommend using the REML instead of the NCHGN for the estimation of *τ*^2^. Finally, no methods perform well when the number of studies is very small (*K*=3), especially for sparse data, but the REML is somewhat better overall.

The design of our simulations, which used equal sample sizes and equal probabilities in all studies may be considered a limitation. However, we would not expect better performance of the GLMMs in a more realistic scenario. At the moment, it is difficult to recommend the use of GLMMs in the practice of meta-analysis.

We believe that the bias in the estimation of *θ* in the NCHGN model is the result of the exponential transformation of the random effect in the noncentral hypergeometric-normal model (8). Similarly, the biases in the FIM and the RIM may be due to the expit transformation of the random effect necessary to obtain the probability of an event in the treatment group. The biases of order 1/*N* are well known in fixed effect and mixed effects models. Nemes et al. [41] show that logistic regression overestimates the odds ratio because of bias of order 1/*N* in studies with small and moderate sample sizes. Kosmidis et al. [42] studied bias of order 1/*N* in the maximum-likelihood estimates of the overall effect measure and the between-study variance under the normal random-effects model. However, the transformation biases in the mixed effects models are of order 1, as discussed in [6]. The problem of finding reasonably good methods of the meta-analysis for binary outcomes is still open.

## Additional files

Additional file 1 - metafor syntax for GLMM in R. This file provides information on implementation of GLMM models in R metafor package. (PDF 77 kb)

Additional file 2 - Simulation results for *p*_*i*2_=0.1, *p*_*i*2_=0.2 and *p*_*i*2_=0.4 under default settings. This file provides additional simulation results under default settings of rma.glmm function in R metafor package. (PDF 586 kb)

Additional file 3 - Simulation results for K=3, *p*_*i*2_=0.1,*θ*=0 and *θ*=1 under default settings. (PDF 223 kb)

Additional file 4 - Simulation results for comparison of dFNCHypergeo and dnoncenhypergeom in a conditional GLMM with exact likelihood. This file provides results of simulations with two methods for fitting the non-central hypergeometric distribution in R (PDF 147 kb)

Additional file 5 - Computational issues. This file provides Figures for convergence and estimation quality of alternative optimizers. (PDF 232 kb)

Additional file 6 - R code for GLMM analysis of simulated data in “Computational issues” section. This file provides R code for GLMM analysis of simulated data in “Computational issues” section. (PDF 122 kb)

Additional file 7 - R code for an example where the optimizer “uobyqa” hangs. This file provides R code and output for an example where the optimizer “uobyqa” hangs. (PDF 107 kb)

## Abbreviations

ABNM: binomial-normal model
CM.AL: In *metafor*, a conditional generalized linear mixed-effects model (approximate likelihood)
CM.EL: In *metafor*, a conditional generalized linear mixed-effects model (exact likelihood)
DL: Der-Simonian and Laird
REML: restricted maximum likelihood
EM: Expectation maximization
FEM: Fixed effect model
FIM: Fixed intercept model
GEE: Generalized estimation equation, GHQ: Gauss-Hermite quadrature
GLMM: Generalized linear mixed models
LOR: Log-odds ratio
MCMC: Markov-chain Monte Carlo
NCHGN: Non-central-hypergeometric-normal model
OR: Odds ratio
PQL: Penalized quasi likelihood
REM: Random effects model
RIM: Random intercept model
RR: Relative risk
UM.FS: In *metafor*, an unconditional generalized linear mixed-effects model with fixed study effects
UM.FS: In *metafor*, an unconditional generalized linear mixed-effects model with fixed study effects

## References

[1] Higgins J, Thompson SG, Spiegelhalter DJ (2009). A re-evaluation of random-effects meta-analysis. J R Stat Soc Ser A (Stat Soc).

[2] Mosteller F, Colditz GA (1996). Understanding research synthesis (meta-analysis). Annu Rev Public Health.

[3] Stijnen T, Hamza TH, Özdemir P (2010). Random effects meta-analysis of event outcome in the framework of the generalized linear mixed model with applications in sparse data. Stat Med.

[4] Kulinskaya E, Morgenthaler S, Staudte RG (2014). Combining statistical evidence. Int Stat Rev.

[5] Hoaglin DC (2016). Misunderstandings about *Q* and ’Cochran’s *Q* test’ in meta-analysis. Stat Med.

[6] Bakbergenuly I, Kulinskaya E, Morgenthaler S (2016). Inference for binomial probability based on dependent Bernoulli random variables with applications to meta-analysis and group level studies. Biom J.

[7] Viechtbauer W. Package *metafor*. The Comprehensive R Archive Network. Package ‘metafor’. http://cran.r-project.org/web/packages/metafor/metafor.pdf. 2017. Accessed 19 Feb 2017.

[8] Lee KJ, Thompson SG (2008). Flexible parametric models for random-effects distributions. Stat Med.

[9] Kuss O (2015). Statistical methods for meta-analyses including information from studies without any events—add nothing to nothing and succeed nevertheless. Stat Med.

[10] Bakbergenuly I, Kulinskaya E (2017). Beta-binomial model for meta-analysis of odds ratios. Stat Med.

[11] Sinclair JC, Bracken MB (2014). Clinically useful measures of effect in binary analyses of randomized trials. J Clin Epidemiol.

[12] Sackett DL, Deeks JJ, Altman DG (1996). Down with odds ratios!. Evid-Based Med.

[13] Altman DG, Deeks JJ, Sackett DL (1998). Odds ratios should be avoided when events are common (letter to the editor). BMJ.

[14] Deeks JJ (2002). Issues in the selection of a summary statistic for meta-analysis of clinical trials with binary outcomes. Stat Med.

[15] Newcombe RG (2006). A deficiency of the odds ratio as a measure of effect size. Stat Med.

[16] Turner RM, Omar RZ, Yang M, Goldstein H, Thompson SG (2000). A multilevel model framework for meta-analysis of clinical trials with binary outcomes. Stat Med.

[17] Van Houwelingen HC, Zwinderman KH, Stijnen T (1993). A bivariate approach to meta-analysis. Stat Med.

[18] Liu Q, Pierce DA (1993). Heterogeneity in Mantel-Haenszel-type models. Biometrika.

[19] Sidik K, Jonkman JN (2008). Estimation using non-central hypergeometric distributions in combining 2 × 2 tables. J Stat Plan Infer.

[20] DerSimonian R, Laird N (1986). Meta-analysis in clinical trials. Control Clin Trials.

[21] Jackson D, Law M, Stijnen T, Viechtbauer W, White IR (2018). A comparison of 7 random-effects models for meta-analyses that estimate the summary odds ratio. Stat Med.

[22] Breslow NE, Clayton DG (1993). Approximate inference in generalized linear mixed models. J Am Stat Assoc.

[23] Demidenko E (2004). Mixed Models: Theory and Applications.

[24] Capanu M, Gönen M, Begg CB (2013). An assessment of estimation methods for generalized linear mixed models with binary outcomes. Stat Med.

[25] Platt RW, Leroux BG, Breslow N (1999). Generalized linear mixed models for meta-analysis. Stat Med.

[26] Gao S (2004). Combining binomial data using the logistic normal model. J Stat Comput Simul.

[27] Hamza TH, Van Houwelingen HC, Stijnen T (2008). The binomial distribution of meta-analysis was preferred to model within-study variability. J Clin Epidemiol.

[28] Liang KY, Zeger SL (1986). Longitudinal data analysis using generalized linear models. Biometrika.

[29] Viechtbauer W (2010). Conducting meta-analyses in *R* with the *metafor* package. J Stat Softw.

[30] Liang KY (1985). Odds ratio inference with dependent data. Biometrika.

[31] Fog A, Fog MA. The *BiasedUrn* package in *R*. 2015. http://cran.r-project.org/web/packages/BiasedUrn/BiasedUrn.pdf. Accessed 19 Feb 2017.

[32] Martin AD, Quinn KM, Park JH, Park MJH. The *MCMCpack* package in *R*. 2016. https://cran.r-project.org/web/packages/MCMCpack/MCMCpack.pdf. Accessed 19 Feb 2017.

[33] Gay DM (1990). Usage summary for selected optimization routines. Comput Sci Tech Rep.

[34] Powell MJ (2009). The bobyqa algorithm for bound constrained optimization without derivatives.

[35] Viechtbauer W (2007). Confidence intervals for the amount of heterogeneity in meta-analysis. Stat Med.

[36] Collins R, Yusuf S, Peto R (1985). Overview of randomised trials of diuretics in pregnancy. Br Med J (Clin Res Ed).

[37] Hardy RJ, Thompson SG (1996). A likelihood approach to meta-analysis with random effects. Stat Med.

[38] Biggerstaff BJ, Tweedie RL (1997). Incorporating variability in estimates of heterogeneity in the random effects model in meta-analysis. Stat Med.

[39] Kulinskaya E, Olkin I (2014). An overdispersion model in meta-analysis. Stat Model.

[40] Viechtbauer W (2005). Bias and efficiency of meta-analytic variance estimators in the random-effects model. J Educ Behav Stat.

[41] Nemes S, Jonasson J, Genell A, Steineck G (2009). Bias in odds ratios by logistic regression modelling and sample size. BMC Med Res Methodol.

[42] Kosmidis I, Guolo A, Varin C (2017). Improving the accuracy of likelihood-based inference in meta-analysis and meta-regression. Biometrika.

