# Supplementary material for: Meta-analysis of binary outcomes via generalized linear mixed models: a simulation study
Source: BMC Med Res Methodol. 2018 Jul 4;18:70. doi: 10.1186/s12874-018-0531-9 (PMC6032567; doi:10.1186/s12874-018-0531-9)
Supplement: Supplementary file 1 — - metafor syntax for GLMM in R. This file provides information on implementation of GLMM models in R metafor package. (PDF 77 kb) [file 12874_2018_531_MOESM1_ESM.pdf]

Additional file 1 - metafor syntax for GLMM in R

In *metafor*, the NCHGN model is specified as

$$rma.glmm(measure = \text{``}OR\text{''}, ai =, bi =, ci =, di =, data =, model = \text{``}CM.EL\text{''}).$$

To use the "dnoncenhypergeom" function for noncentral-hypergeometric distribution or a specific optimizer such as "optim","bobyqa", "newuoa", or "uobyqa" , the model should be specified as

$$rma.glmm(measure = \text{``}OR\text{''}, ai =, bi =, ci =, di =,$$

$$model = \text{``}CM.EL\text{''}, control = list(dnchgcalc = \text{``}dnoncenhypergeom\text{''}))$$

and

$$rma.glmm(measure = \text{``}OR\text{''}, ai =, bi =, ci =, di =,$$

$$model = \text{``}CM.EL\text{''}, control = list(optimizer = \text{''}optim\text{''}))$$

where ai, bi ci, di are the binomial data from the table

	Event	No event	Total
Treatment	ai	bi	$ai + bi$
Control	ci	di	$ci + di$
Total	$ai + ci$	$bi + di$	$ai + bi + ci + di$

The two methods ("dFNCHypergeo" and "dnoncenhypergeom") should perform similarly, when fitting the conditional GLMM (exact likelihood). However, convergence problems might occur when trying to fit a saturated model. Switching to an alternative method can help to solve the problem.
With small numbers of events, the binomial-normal approximation may be used. Then the model is a conditional GLMM (approximate likelihood). This model is specified as

$$rma.glmm(measure = \text{``}OR\text{''}, ai =, bi =, ci =, di =, data =, model = \text{``}CM.AL\text{''}).$$
