# Supplementary material for: Meta-analysis of binary outcomes via generalized linear mixed models: a simulation study
Source: BMC Med Res Methodol. 2018 Jul 4;18:70. doi: 10.1186/s12874-018-0531-9 (PMC6032567; doi:10.1186/s12874-018-0531-9)
Supplement: Supplementary file 2 — - Simulation results for pi2=0.1, pi2=0.2 and pi2=0.4 under default settings. This file provides additional simulation results under default settings of rma.glmm function in R metafor package. (PDF 586 kb) [file 12874_2018_531_MOESM2_ESM.pdf]

# Additional file 2 - Simulation results for $p_{i2} = 0.1$, $p_{i2} = 0.2$ and $p_{i2} = 0.4$ under default settings

Figures A4-A6 show the results for bias of estimation of between-study variance $\tau^2$, bias of estimates of overall effect measure $\hat{\theta}$ and estimated coverage of overall effect measure $\hat{\theta}$ in the random-effects model when $p_{i2} = 0.1$, $\theta = 1$. Similarly, Figures A7-A12 and Figures A13-A18 show the results of simulations for $p_{i2} = 0.2$ and $p_{i2} = 0.4$ with $\theta = 0$, $\theta = 1$ respectively.
Some of the Figures; Figure A4, Figure A8, Figure A13, Figure A14, show the erratic behaviour in performance of a conditional GLMM with an exact likelihood (NCHGN) when $N = 250$ and $N = 1000$. The erratic behaviour appears mostly when the true value of log-odds ratio $\theta$ changes its value from 0 to 1 for $p_{1i} = 0.1, 0.2, 0.4$ apart from the case in Figure A13 when $\theta = 0$. The reason for erratic behaviour is the appearance of huge estimates of $\tau^2$ in some of the repetitions across 10000 iterations. Please see Section 3.2 on page 12 of main text for an example of data simulated from standard REM and corresponding Figure 4.
We also do not exclude the possibility that the huge estimate of $\tau^2$ may be due to nonstability of implementations in obtaining the probability mass function of the noncentral hypergeometric distribution by specifications dFNCHypergeo and dnoncenhypergeom when fitting the model in R programming language.

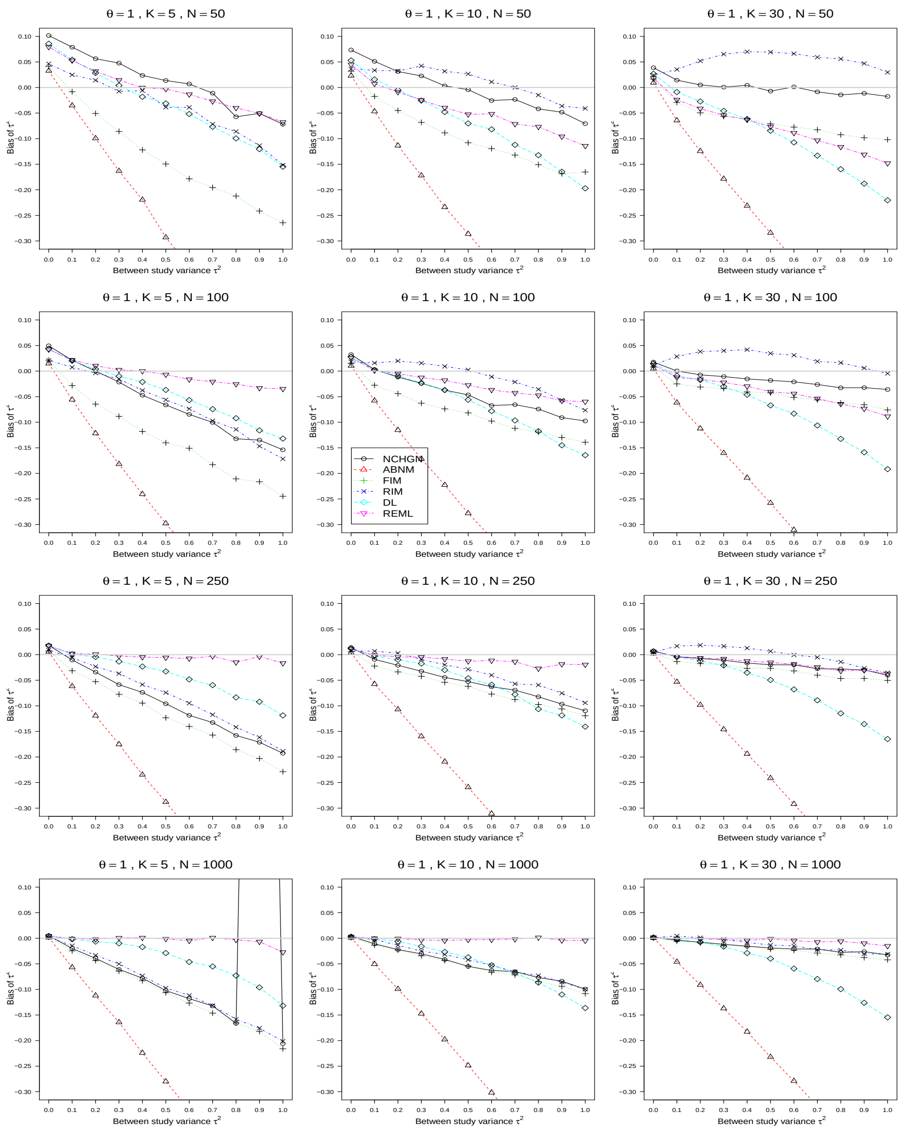


Figure A4: **Bias of $\tau^2$ in the REM when $p_{i2} = 0.1, \theta = 1, 0 \leq \tau^2 \leq 1$ and $n = 50, 100, 250, 1000$.** Estimation methods are: pluses - unconditional generalized linear mixed-effects model with fixed study effects (FIM), crosses - unconditional generalized linear mixed-effects model with random study effects (RIM), circles - a conditional generalized linear mixed-effects model with exact likelihood (NCHGN), triangles - a conditional generalized linear mixed-effects model with approximate likelihood (ABNM), rhombs - DerSimonian and Laird method (DL) and reverse triangles - restricted maximum likelihood method (REML). Light grey line at 0.

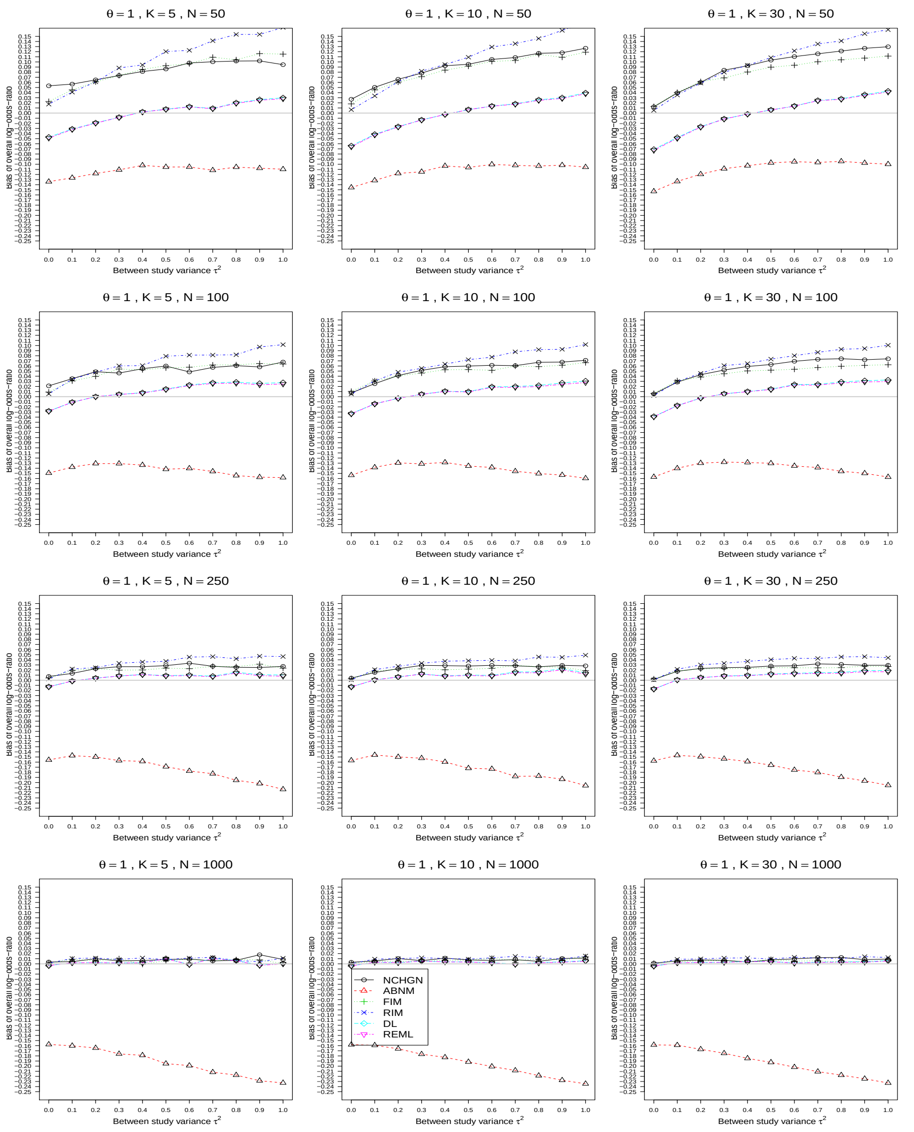


Figure A5: **Bias of $\theta$ in the REM when $p_{i2} = 0.1$, $\theta = 1$, $0 \leq \tau^2 \leq 1$ and $n = 50, 100, 250, 1000$.** Estimation methods are: pluses - unconditional generalized linear mixed-effects model with fixed study effects (FIM), crosses - unconditional generalized linear mixed-effects model with random study effects (RIM), circles - a conditional generalized linear mixed-effects model with exact likelihood (NCHGN), triangles - a conditional generalized linear mixed-effects model with approximate likelihood (ABNM), rhombs - DerSimonian and Laird method (DL) and reverse triangles - restricted maximum likelihood method (REML). Light grey line at 0.

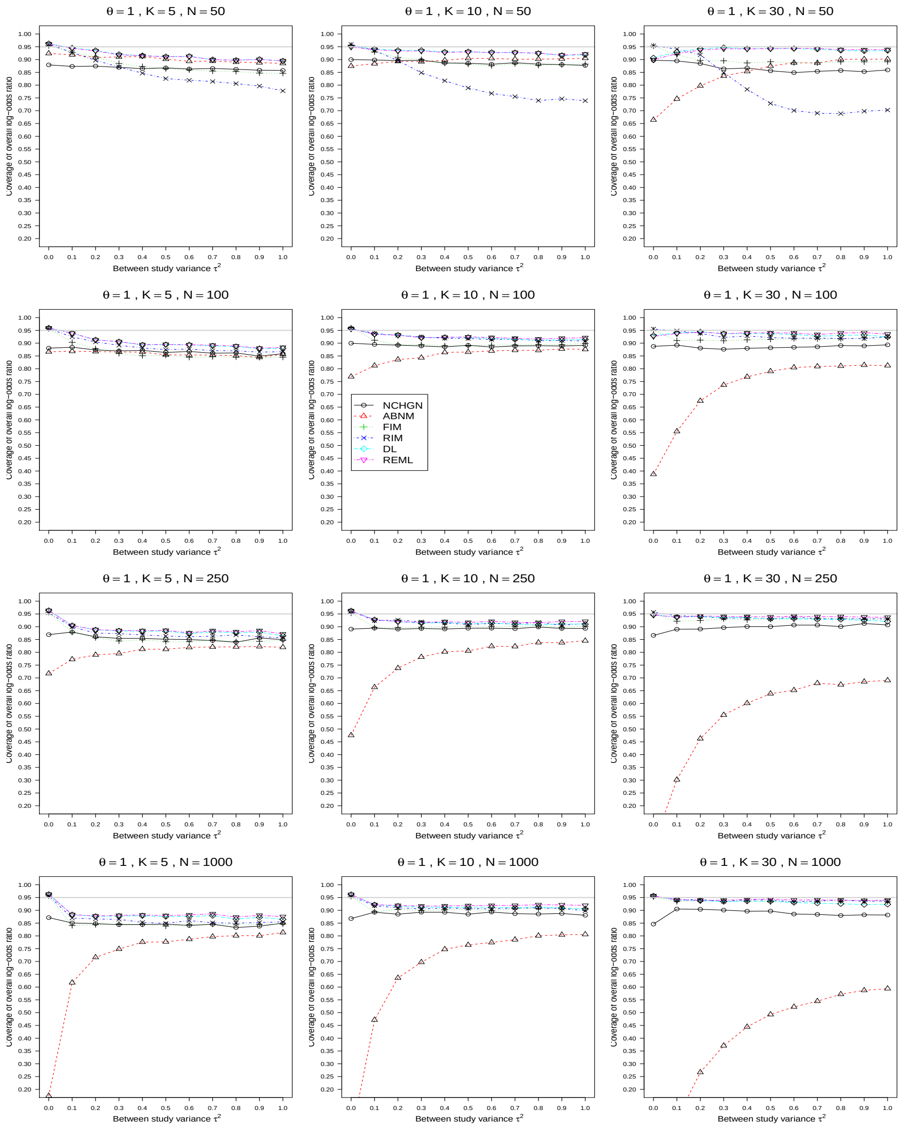


Figure A6: **Estimated coverage of $\theta$ in the REM when $p_{i2} = 0.1$, $\theta = 1$, $0 \leq \tau^2 \leq 1$ and $n = 50, 100, 250, 1000$.** The coverages are given at the nominal 95% level. Estimation methods are: pluses - unconditional generalized linear mixed-effects model with fixed study effects (FIM), crosses - unconditional generalized linear mixed-effects model with random study effects (RIM), circles - a conditional generalized linear mixed-effects model with exact likelihood (NCHGN), triangles - a conditional generalized linear mixed-effects model with approximate likelihood (ABNM), rhombs - DerSimonian and Laird method (DL) and reverse triangles - restricted maximum likelihood method (REML). Light grey line at 0.95.

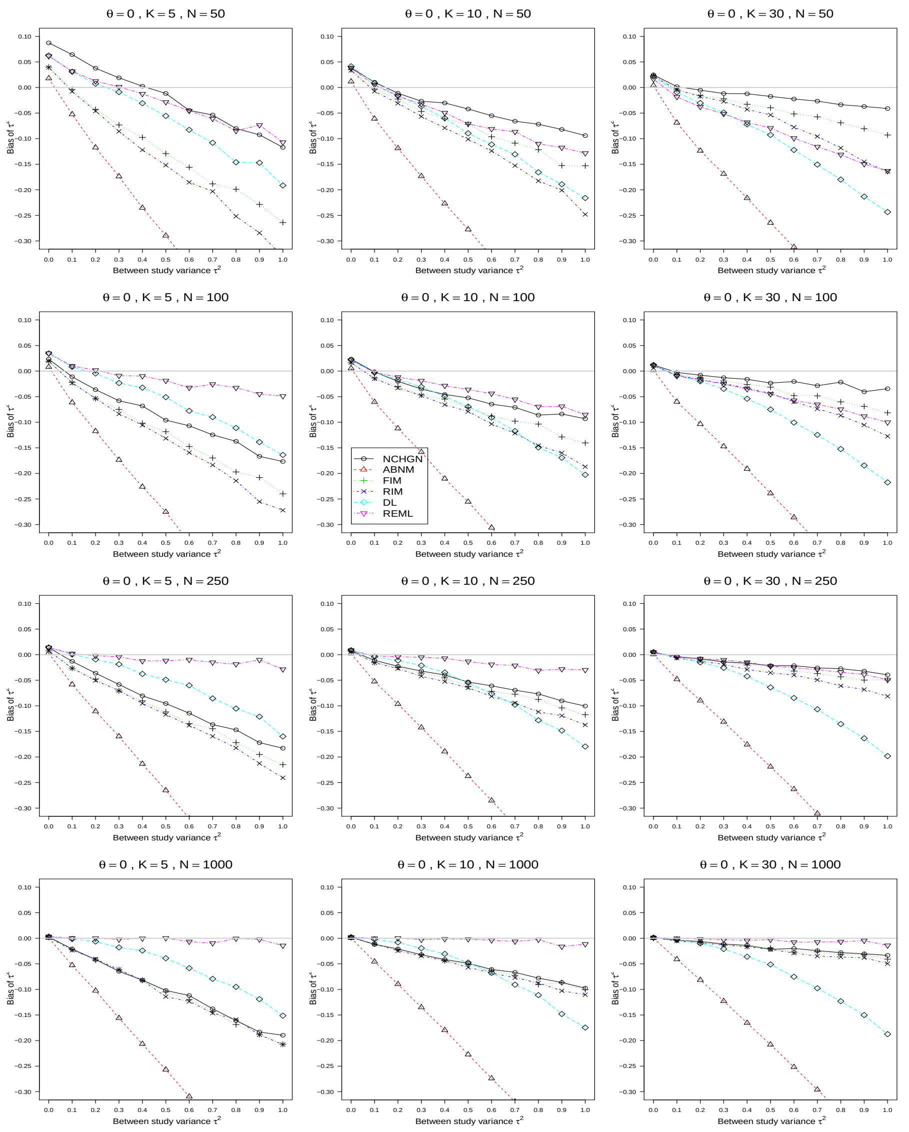


Figure A7: **Bias of $\tau^2$ in the REM when $p_{i2} = 0.2, \theta = 0, 0 \leq \tau^2 \leq 1$ and $n = 50, 100, 250, 1000$.** Estimation methods are: pluses - unconditional generalized linear mixed-effects model with fixed study effects (FIM), crosses - unconditional generalized linear mixed-effects model with random study effects (RIM), circles - a conditional generalized linear mixed-effects model with exact likelihood (NCHGN), triangles - a conditional generalized linear mixed-effects model with approximate likelihood (ABNM), rhombs - DerSimonian and Laird method (DL) and reverse triangles - restricted maximum likelihood method (REML). Light grey line at 0.

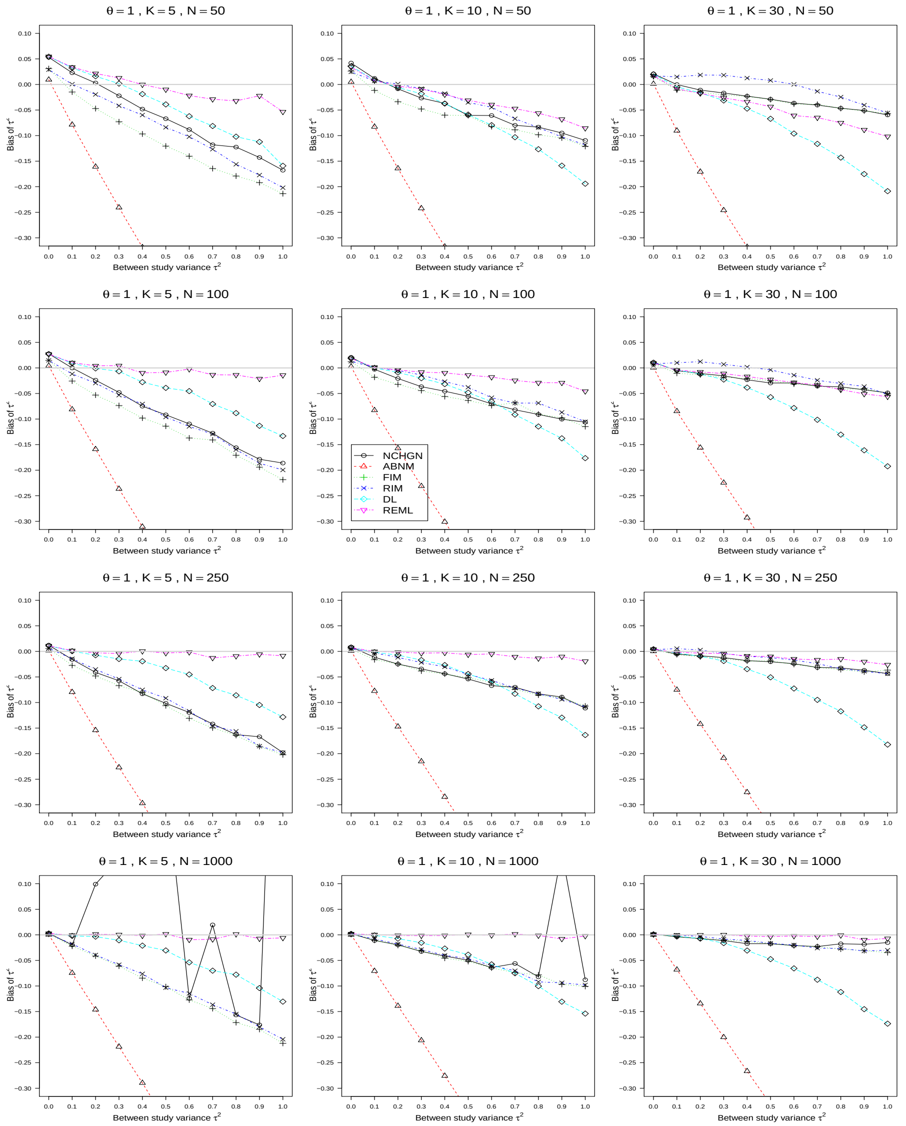


Figure A8: **Bias of $\tau^2$ in the REM when $p_{i2} = 0.2, \theta = 1, 0 \leq \tau^2 \leq 1$ and $n = 50, 100, 250, 1000$.** Estimation methods are: pluses - unconditional generalized linear mixed-effects model with fixed study effects (FIM), crosses - unconditional generalized linear mixed-effects model with random study effects (RIM), circles - a conditional generalized linear mixed-effects model with exact likelihood (NCHGN), triangles - a conditional generalized linear mixed-effects model with approximate likelihood (ABNM), rhombs - DerSimonian and Laird method (DL) and reverse triangles - restricted maximum likelihood method (REML). Light grey line at 0.

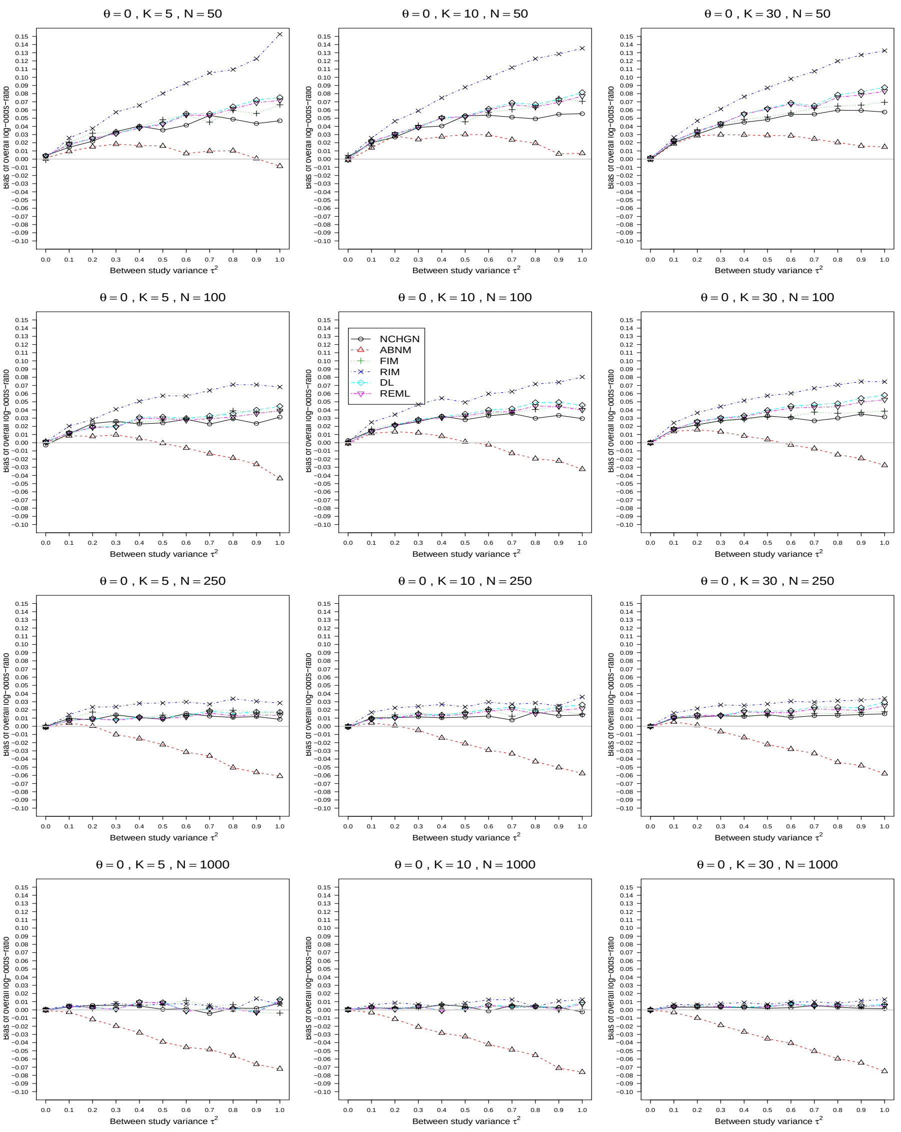


Figure A9: **Bias of $\theta$ in the REM when $p_{i2} = 0.2$, $\theta = 0$, $0 \leq \tau^2 \leq 1$ and $n = 50, 100, 250, 1000$.** Estimation methods are: pluses - unconditional generalized linear mixed-effects model with fixed study effects (FIM), crosses - unconditional generalized linear mixed-effects model with random study effects (RIM), circles - a conditional generalized linear mixed-effects model with exact likelihood (NCHGN), triangles - a conditional generalized linear mixed-effects model with approximate likelihood (ABNM), rhombs - DerSimonian and Laird method (DL) and reverse triangles - restricted maximum likelihood method (REML). Light grey line at 0.

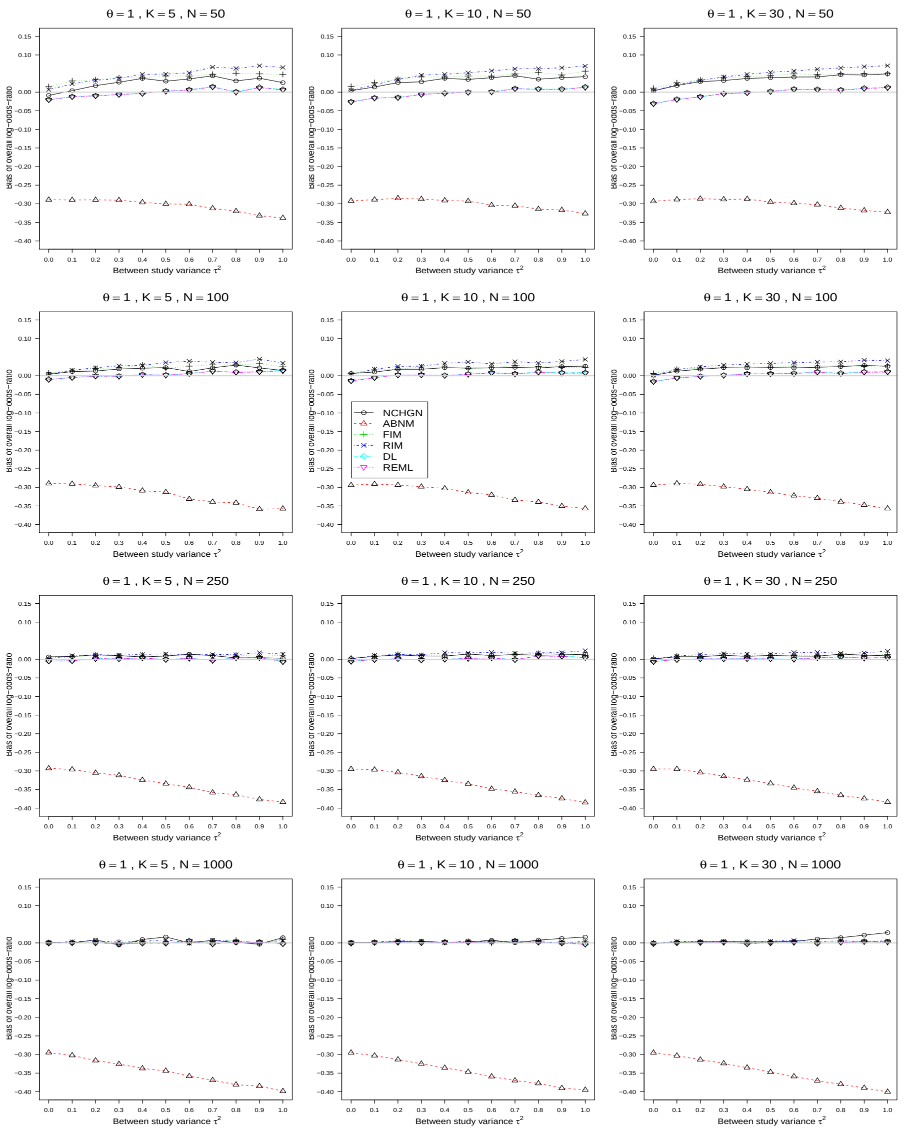


Figure A10: **Bias of $\theta$ in the REM when $p_{i2} = 0.2, \theta = 1, 0 \le \tau^2 \le 1$ and $n = 50, 100, 250, 1000$.** Estimation methods are: pluses - unconditional generalized linear mixed-effects model with fixed study effects (FIM), crosses - unconditional generalized linear mixed-effects model with random study effects (RIM), circles - a conditional generalized linear mixed-effects model with exact likelihood (NCHGN), triangles - a conditional generalized linear mixed-effects model with approximate likelihood (ABNM), rhombs - DerSimonian and Laird method (DL) and reverse triangles - restricted maximum likelihood method (REML). Light grey line at 0.

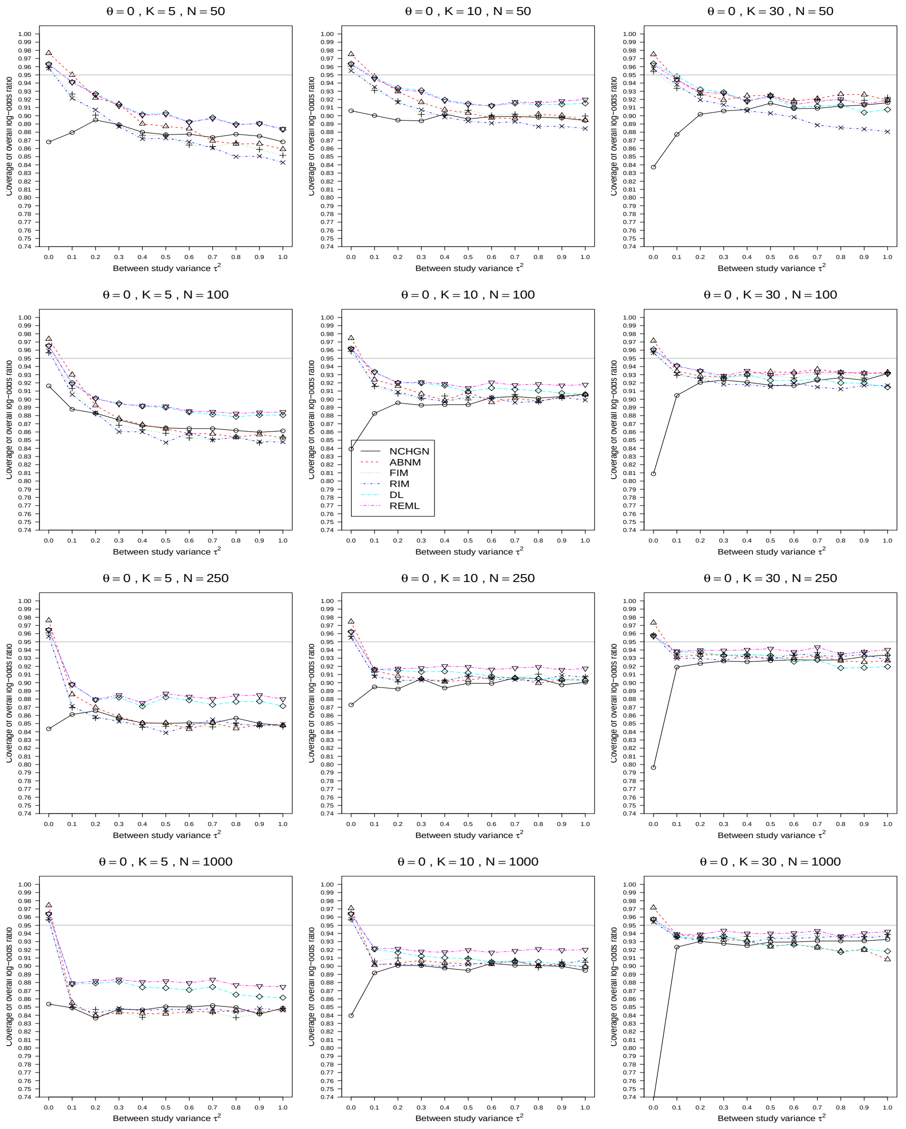


Figure A11: **Estimated coverage of $\theta$ in the REM when $p_{i2} = 0.2$, $\theta = 0$, $0 \leq \tau^2 \leq 1$ and $n = 50, 100, 250, 1000$.** The coverages are given at the nominal 95% level. Estimation methods are: pluses - unconditional generalized linear mixed-effects model with fixed study effects (FIM), crosses - unconditional generalized linear mixed-effects model with random study effects (RIM), circles - a conditional generalized linear mixed-effects model with exact likelihood (NCHGN), triangles - a conditional generalized linear mixed-effects model with approximate likelihood (ABNM), rhombs - DerSimonian and Laird method (DL) and reverse triangles - restricted maximum likelihood method (REML). Light grey line at 0.95.

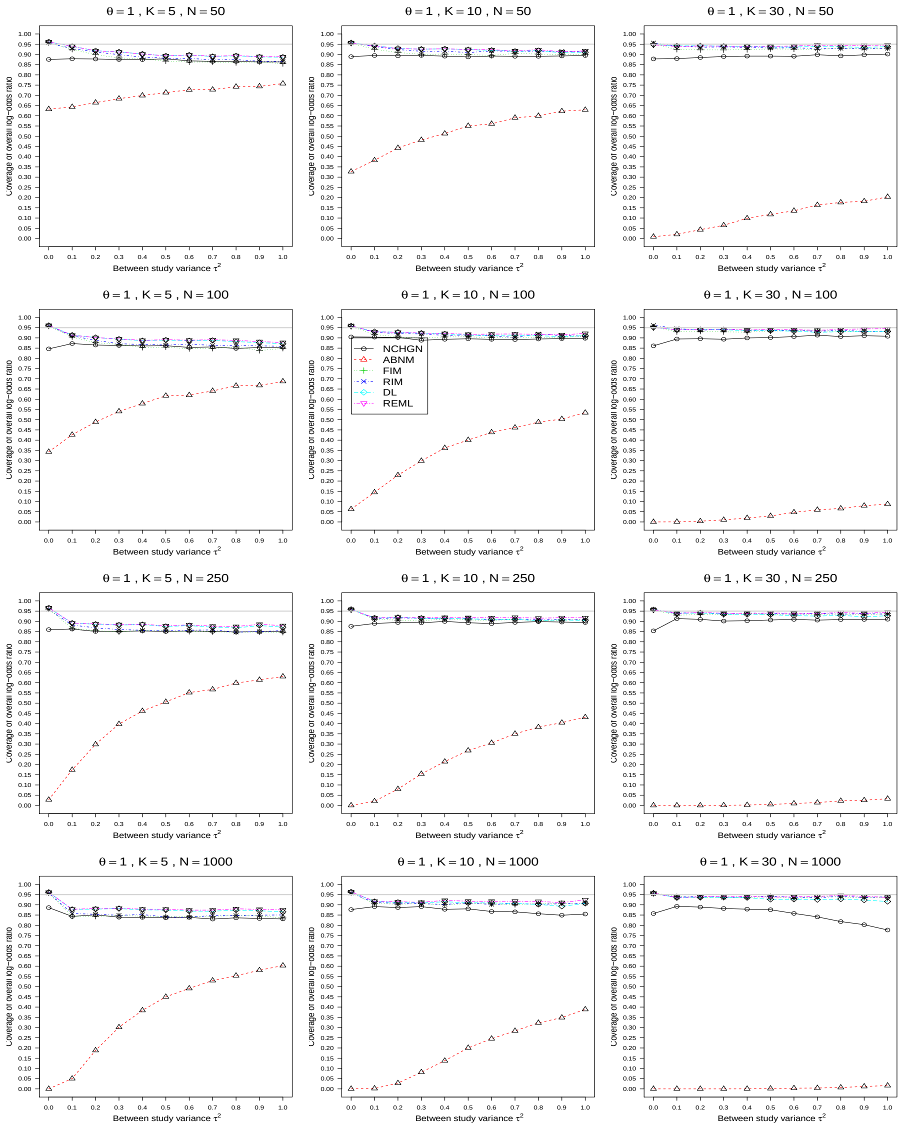


Figure A12: **Estimated coverage of $\theta$ in the REM when $p_{i2} = 0.2$, $\theta = 1$, $0 \leq \tau^2 \leq 1$ and $n = 50, 100, 250, 1000$.** The coverages are given at the nominal 95% level. Estimation methods are: pluses - unconditional generalized linear mixed-effects model with fixed study effects (FIM), crosses - unconditional generalized linear mixed-effects model with random study effects (RIM), circles - a conditional generalized linear mixed-effects model with exact likelihood (NCHGN), triangles - a conditional generalized linear mixed-effects model with approximate likelihood (ABNM), rhombs - DerSimonian and Laird method (DL) and reverse triangles - restricted maximum likelihood method (REML). Light grey line at 0.95.

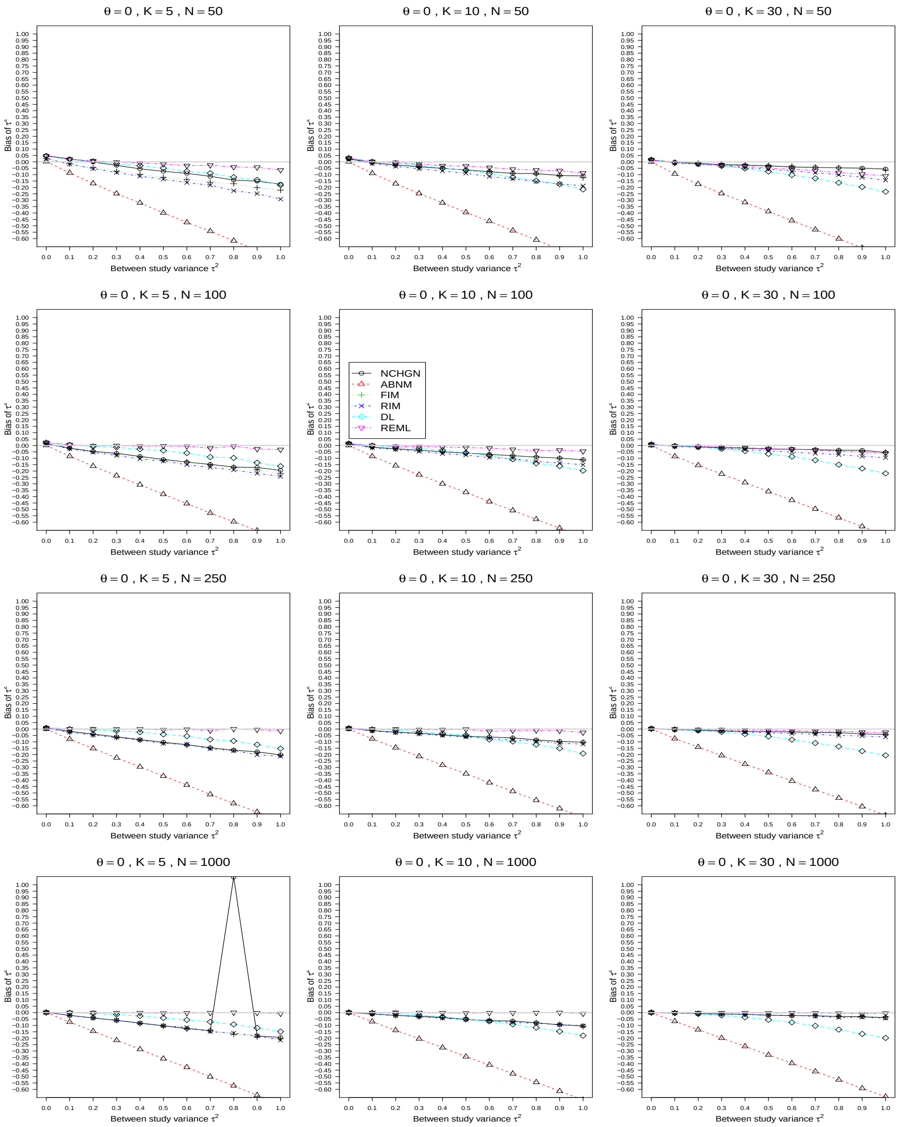


Figure A13: **Bias of $\tau^2$ in the REM when $p_{i2} = 0.4$, $\theta = 0$, $0 \leq \tau^2 \leq 1$ and $n = 50, 100, 250, 1000$.** Estimation methods are: pluses - unconditional generalized linear mixed-effects model with fixed study effects (FIM), crosses - unconditional generalized linear mixed-effects model with random study effects (RIM), circles - a conditional generalized linear mixed-effects model with exact likelihood (NCHGN), triangles - a conditional generalized linear mixed-effects model with approximate likelihood (ABNM), rhombs - DerSimonian and Laird method (DL) and reverse triangles - restricted maximum likelihood method (REML). Light grey line at 0.

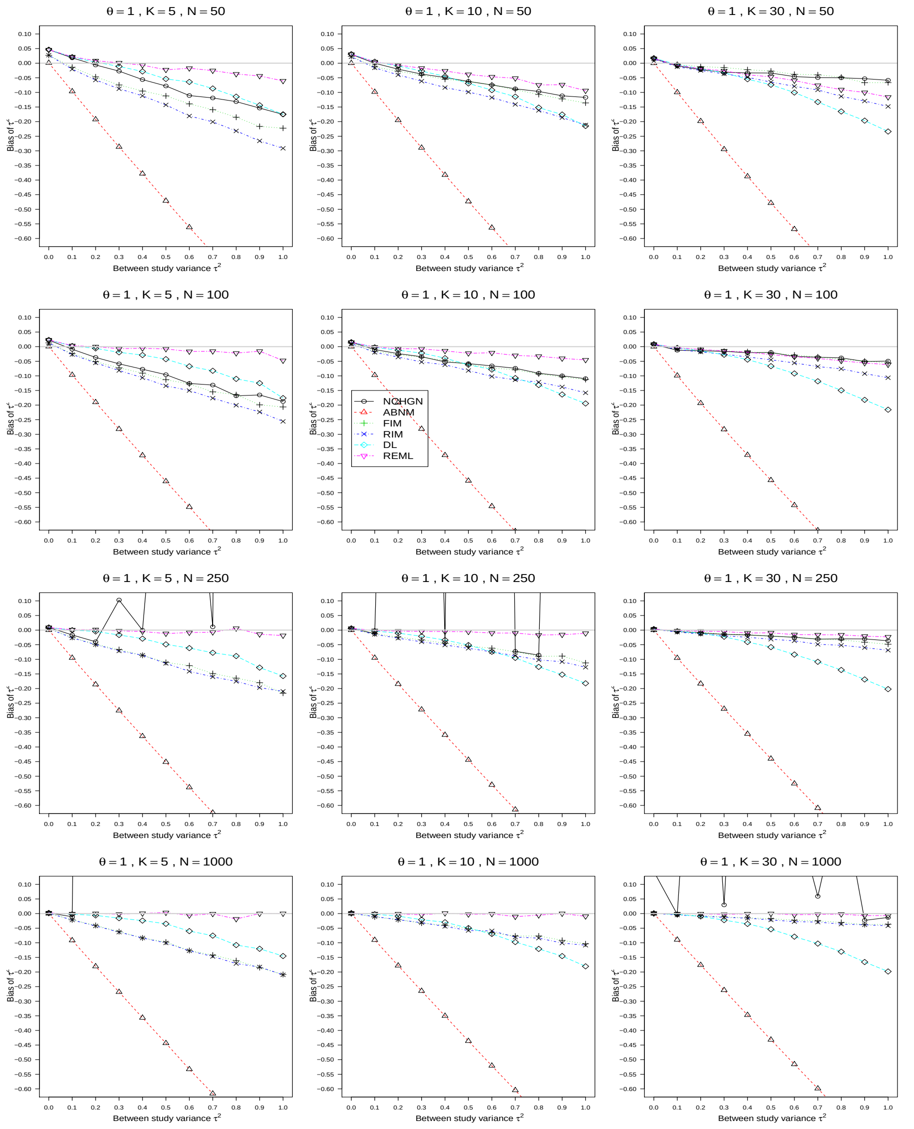


Figure A14: **Bias of $\tau^2$ in the REM when $p_{i2} = 0.4$, $\theta = 1$, $0 \leq \tau^2 \leq 1$ and $n = 50, 100, 250, 1000$.** Estimation methods are: pluses - unconditional generalized linear mixed-effects model with fixed study effects (FIM), crosses - unconditional generalized linear mixed-effects model with random study effects (RIM), circles - a conditional generalized linear mixed-effects model with exact likelihood (NCHGN), triangles - a conditional generalized linear mixed-effects model with approximate likelihood (ABNM), rhombs - DerSimonian and Laird method (DL) and reverse triangles - restricted maximum likelihood method (REML). Light grey line at 0.

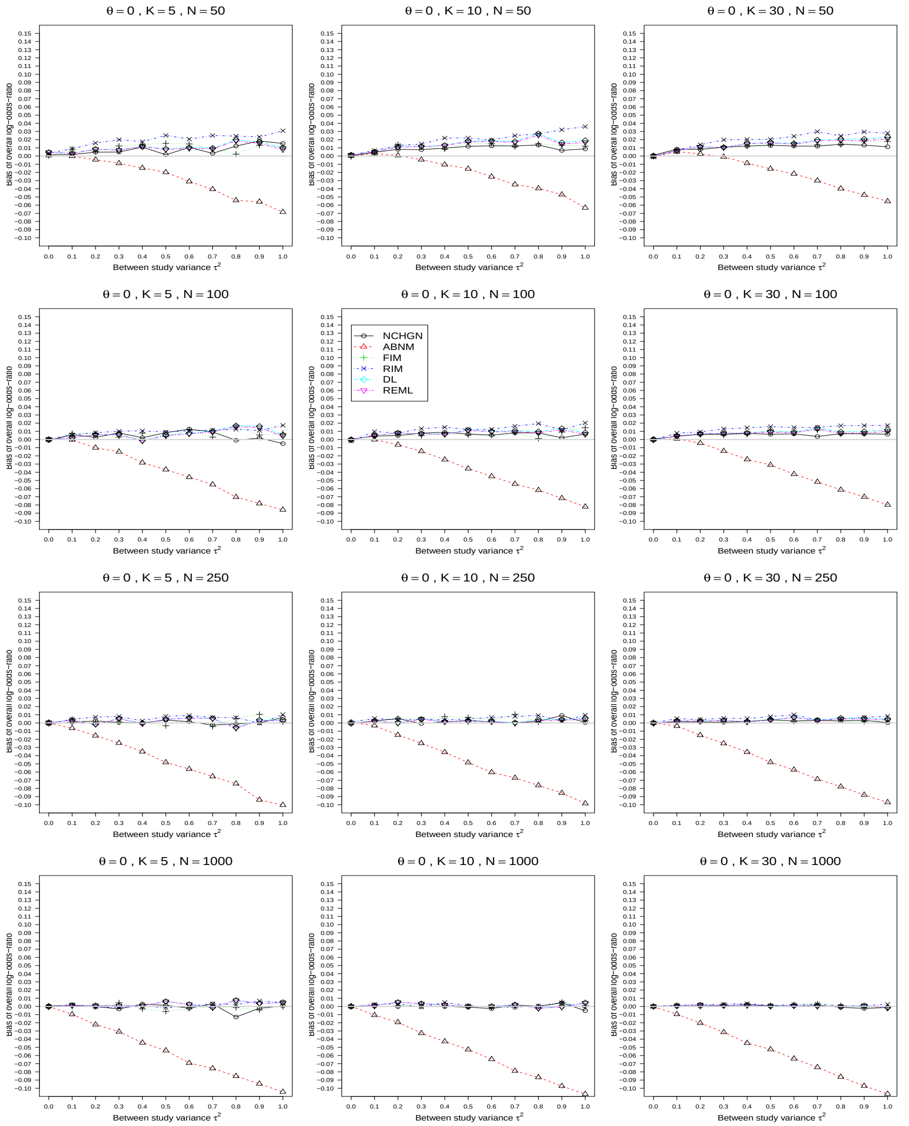


Figure A15: **Bias of $\theta$ in the REM when $p_{i2} = 0.4$, $\theta = 0$, $0 \leq \tau^2 \leq 1$ and $n = 50, 100, 250, 1000$.** Estimation methods are: pluses - unconditional generalized linear mixed-effects model with fixed study effects (FIM), crosses - unconditional generalized linear mixed-effects model with random study effects (RIM), circles - a conditional generalized linear mixed-effects model with exact likelihood (NCHGN), triangles - a conditional generalized linear mixed-effects model with approximate likelihood (ABNM), rhombs - DerSimonian and Laird method (DL) and reverse triangles - restricted maximum likelihood method (REML). Light grey line at 0.

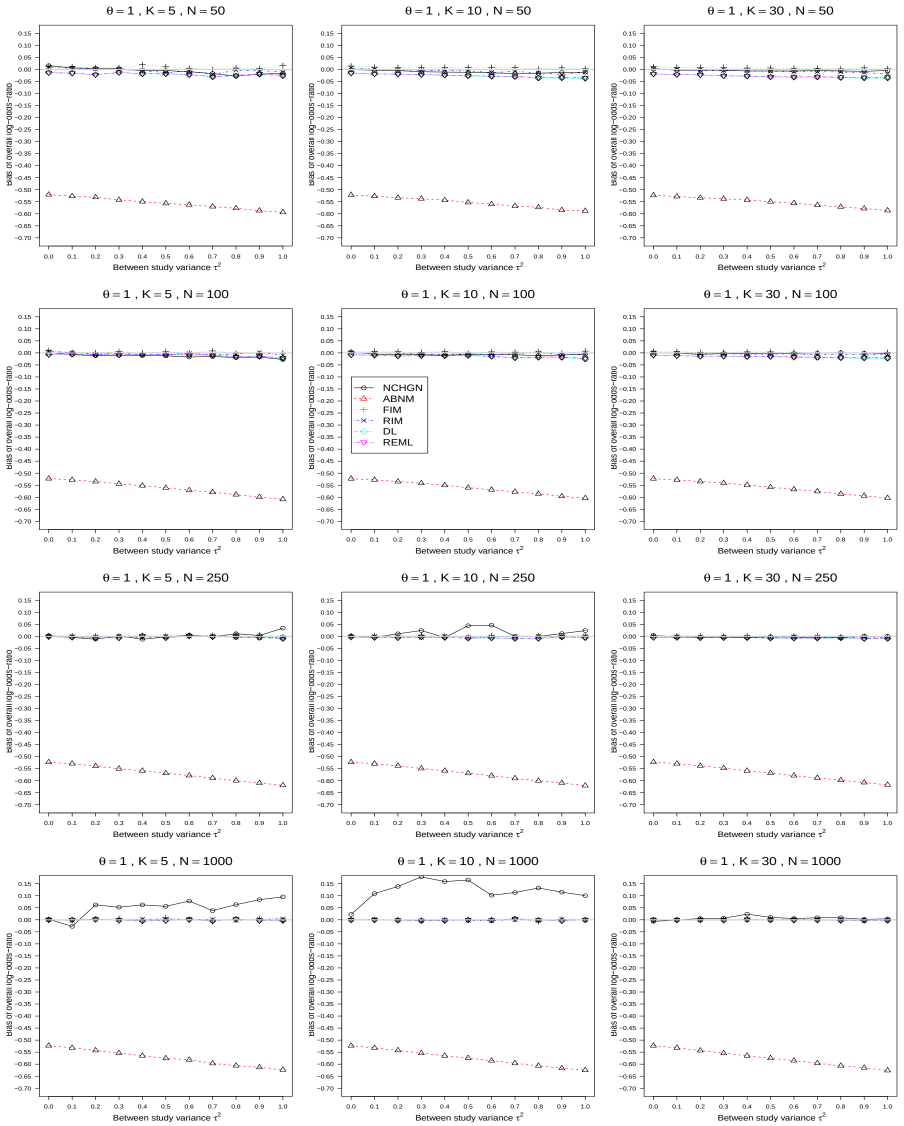


Figure A16: **Bias of $\theta$ in the REM when $p_{i2} = 0.4$, $\theta = 1$, $0 \leq \tau^2 \leq 1$ and $n = 50, 100, 250, 1000$.** Estimation methods are: pluses - unconditional generalized linear mixed-effects model with fixed study effects (FIM), crosses - unconditional generalized linear mixed-effects model with random study effects (RIM), circles - a conditional generalized linear mixed-effects model with exact likelihood (NCHGN), triangles - a conditional generalized linear mixed-effects model with approximate likelihood (ABNM), rhombs - DerSimonian and Laird method (DL) and reverse triangles - restricted maximum likelihood method (REML). Light grey line at 0.

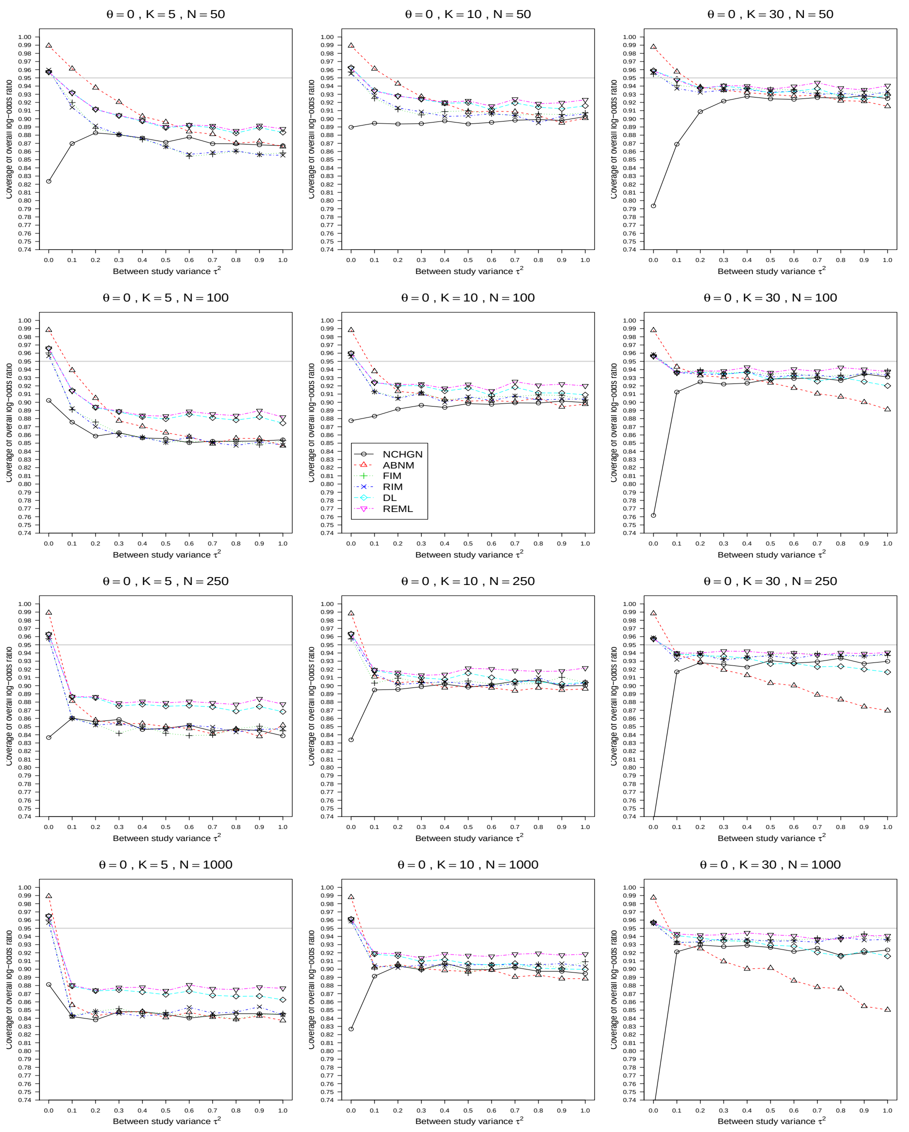


Figure A17: **Estimated coverage of $\theta$ in the REM when $p_{i2} = 0.4$, $\theta = 0$, $0 \leq \tau^2 \leq 1$ and $n = 50, 100, 250, 1000$.** The coverages are given at the nominal 95% level. Estimation methods are: pluses - unconditional generalized linear mixed-effects model with fixed study effects (FIM), crosses - unconditional generalized linear mixed-effects model with random study effects (RIM), circles - a conditional generalized linear mixed-effects model with exact likelihood (NCHGN), triangles - a conditional generalized linear mixed-effects model with approximate likelihood (ABNM), rhombs - DerSimonian and Laird method (DL) and reverse triangles - restricted maximum likelihood method (REML). Light grey line at 0.95.

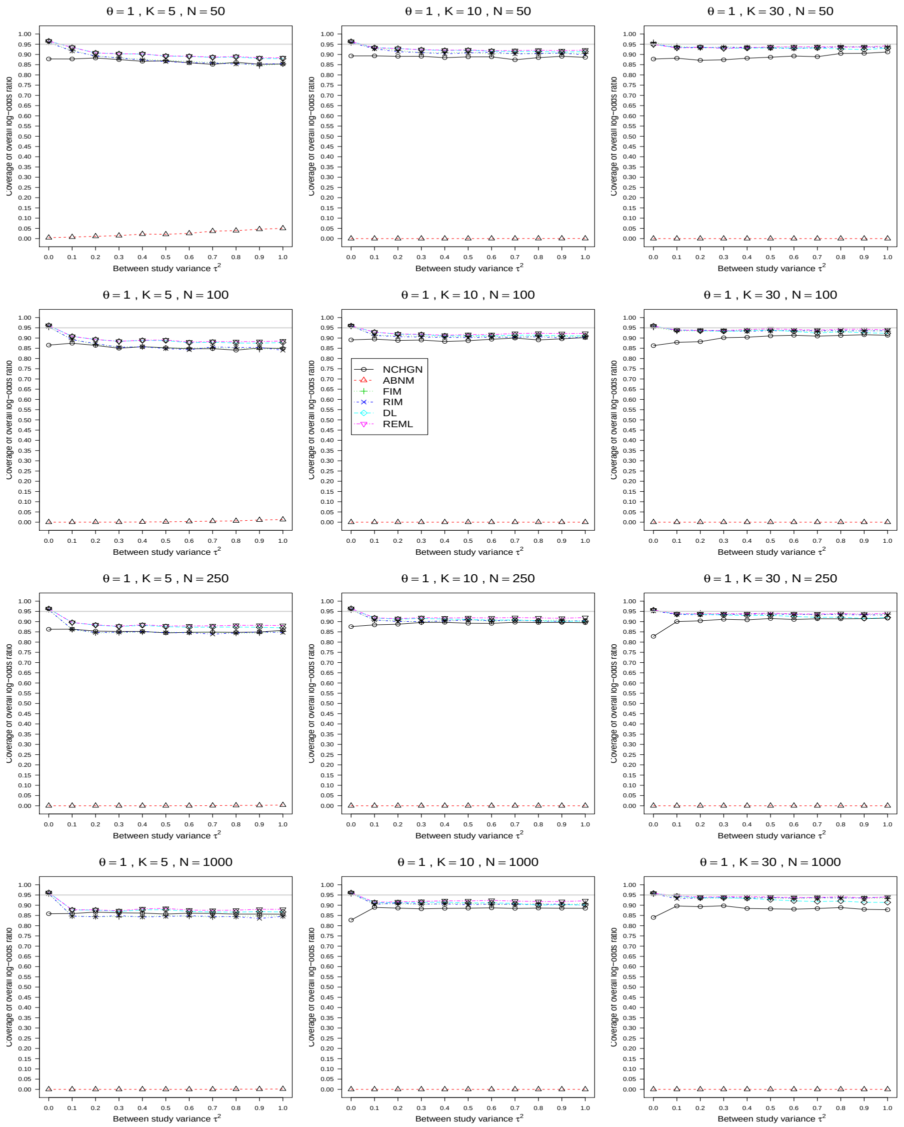


Figure A18: **Estimated coverage of $\theta$ in the REM when $p_{i2} = 0.4$, $\theta = 1$, $0 \leq \tau^2 \leq 1$ and $n = 50, 100, 250, 1000$.** The coverages are given at the nominal 95% level. Estimation methods are: pluses - unconditional generalized linear mixed-effects model with fixed study effects (FIM), crosses - unconditional generalized linear mixed-effects model with random study effects (RIM), circles - a conditional generalized linear mixed-effects model with exact likelihood (NCHGN), triangles - a conditional generalized linear mixed-effects model with approximate likelihood (ABNM), rhombs - DerSimonian and Laird method (DL) and reverse triangles - restricted maximum likelihood method (REML). Light grey line at 0.95.
