# Supplementary material for: Meta-analysis of binary outcomes via generalized linear mixed models: a simulation study
Source: BMC Med Res Methodol. 2018 Jul 4;18:70. doi: 10.1186/s12874-018-0531-9 (PMC6032567; doi:10.1186/s12874-018-0531-9)
Supplement: Supplementary file 3 — - Simulation results for K=3, pi2=0.1,θ=0 and θ=1 under default settings. (PDF 223 kb) [file 12874_2018_531_MOESM3_ESM.pdf]

Additional file 3 - Simulation results for K=3, $p_{i2} = 0.1$, $\theta = 0$ and $\theta = 1$ under default settings

In this section we provide a simulation study to assess the performance of point estimators of random effect parameter τ^2 and the corresponding point/interval estimators of the combined LOR θ from the methods of generalized linear mixed-effects models and standard REM when the number of studies is $K = 3$.

We assess six point estimators of τ^2 in respect to their bias: unconditional generalized linear mixed-effects model with fixed study effects (FIM), unconditional generalized linear mixed-effects model with random study effects (RIM), a conditional generalized linear mixed-effects model with exact likelihood (NCHGN), a conditional generalized linear mixed-effects model with approximate likelihood (ABNM), DerSimonian and Laird method (DL) and restricted maximum likelihood method (REML). We also assess the corresponding bias and coverage of the combined odds ratio from these methods.

Figure A29 and Figure A32 shows the bias of τ^2 when $\theta = 0$ and $\theta = 1$ with $K = 3$ and $p_{i2} = 0.1$, respectively. The bias of τ^2 is not quite similar to the bias in Figure 1 and Figure A5. The bias is still linear over the range of τ^2 and n. When $\theta = 0$ and $N = 50$, the bias of τ^2 from NCHGN is positive and otherwise it is negative in all possible scenarios when $\theta = 0$ or $\theta = 1$. ABNM performs erratically when $\theta = 0$ and $N = 50$, and NCHGN behaves erratically when $\theta = 1$ and $N = 1000$. The standard REML and DL outperform GLMM methods in estimation of τ^2 for $K = 3$, especially for larger values of τ^2.

In respect to estimation of the overall LOR $\hat{\theta}$, Figure A30 and Figure A33 show the bias of overall LOR $\hat{\theta}$. The bias of θ for $K = 3$ is quite similar to the bias of θ when $K = 5$. Among all the methods, the bias of ABNM is still the lowest for $\theta = 0$ with $N \leq 1000$ and the largest for $\theta = 1$. The standard DL and REML methods perform similarly and they are the best for $\theta = 1$. NCHGN performs similarly or better than the standard DL and REML methods for $\theta = 0$.

The coverage of θ, Figure A31 and Figure A34, is still closely related to the bias of its estimation. We can clearly see that standard DL and REML methods perform better than all GLMM methods. The coverage of all GLMM methods is similar in all possible scenarios apart from the case when the coverage of ABNM drops down dramatically for $\theta = 1$ and $N = 1000$. This may be due to the large bias of $\hat{\theta}_{ABNM}$ shown in Figure A33.

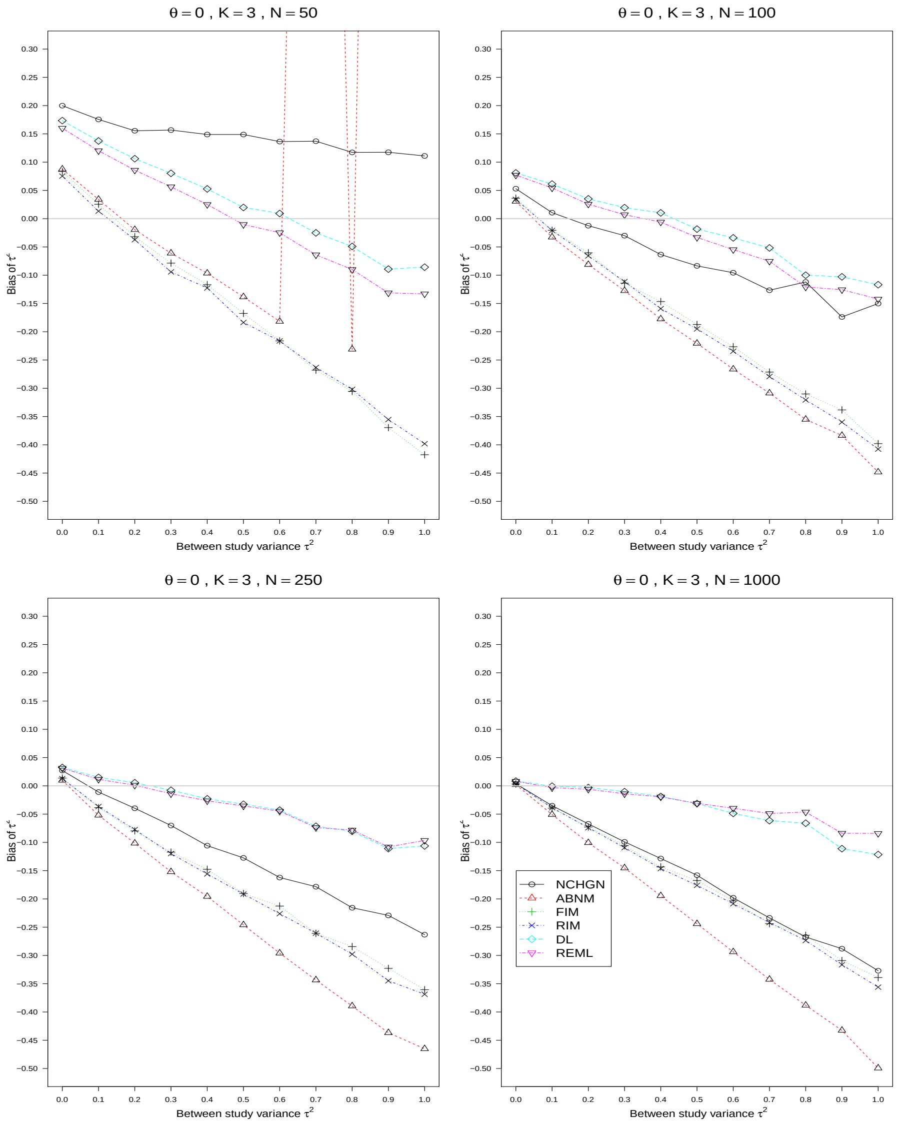

Figure A29: **Bias of τ^2 in the REM when $K = 3$,$p_{i2} = 0.1$, $\theta = 0$, $0 \leq \tau^2 \leq 1$ and $n = 50, 100, 250, 1000$.** Estimation methods are: pluses - unconditional generalized linear mixed-effects model with fixed study effects (FIM), crosses - unconditional generalized linear mixed-effects model with random study effects (RIM), circles - a conditional generalized linear mixed-effects model with exact likelihood (NCHGN), triangles - a conditional generalized linear mixed-effects model with approximate likelihood (ABNM), rhombs - DerSimonian and Laird method (DL) and reverse triangles - restricted maximum likelihood method (REML). Light grey line at 0.

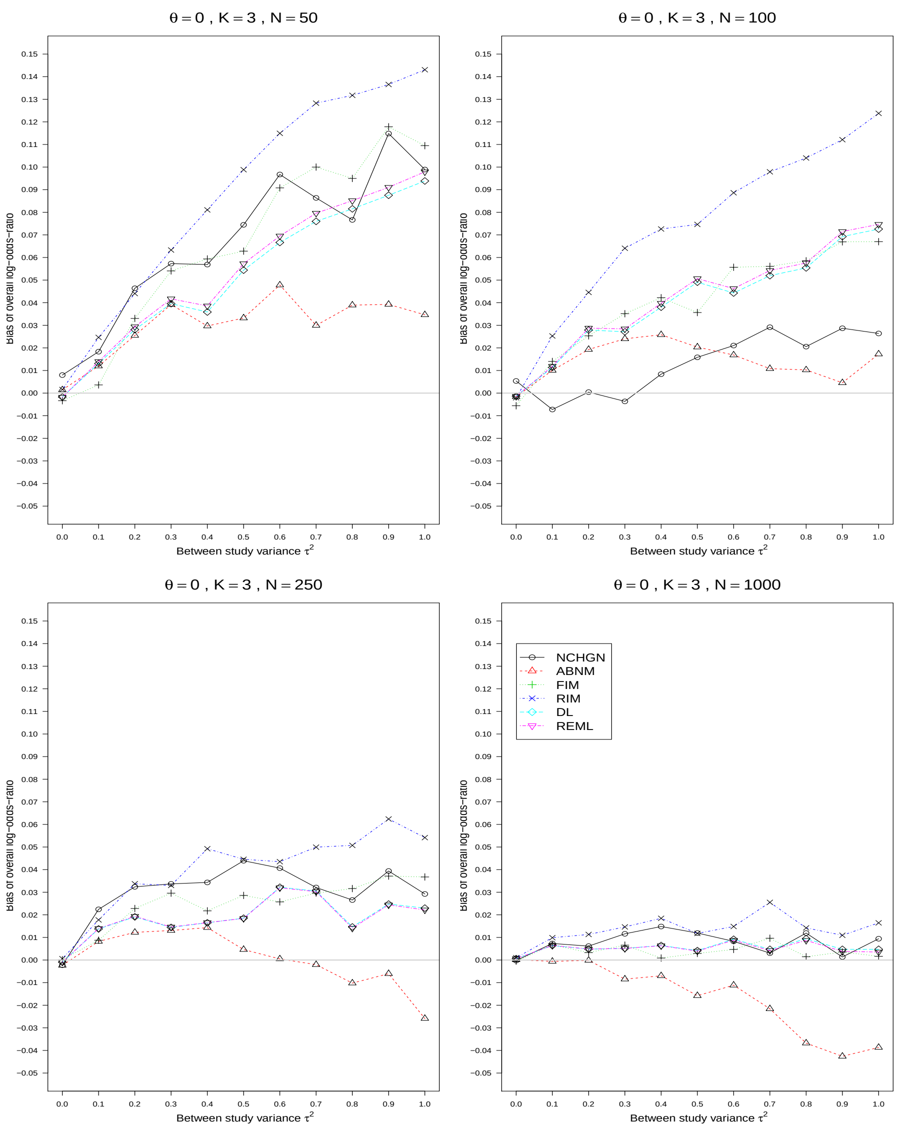

Figure A30: **Bias of θ in the REM when $K = 3$, $p_{i2} = 0.1$, $\theta = 0$, $0 \leq \tau^2 \leq 1$ and $n = 50, 100, 250, 1000$.** Estimation methods are: pluses - unconditional generalized linear mixed-effects model with fixed study effects (FIM), crosses - unconditional generalized linear mixed-effects model with random study effects (RIM), circles - a conditional generalized linear mixed-effects model with exact likelihood (NCHGN), triangles - a conditional generalized linear mixed-effects model with approximate likelihood (ABNM), rhombs - DerSimonian and Laird method (DL) and reverse triangles - restricted maximum likelihood method (REML). Light grey line at 0.

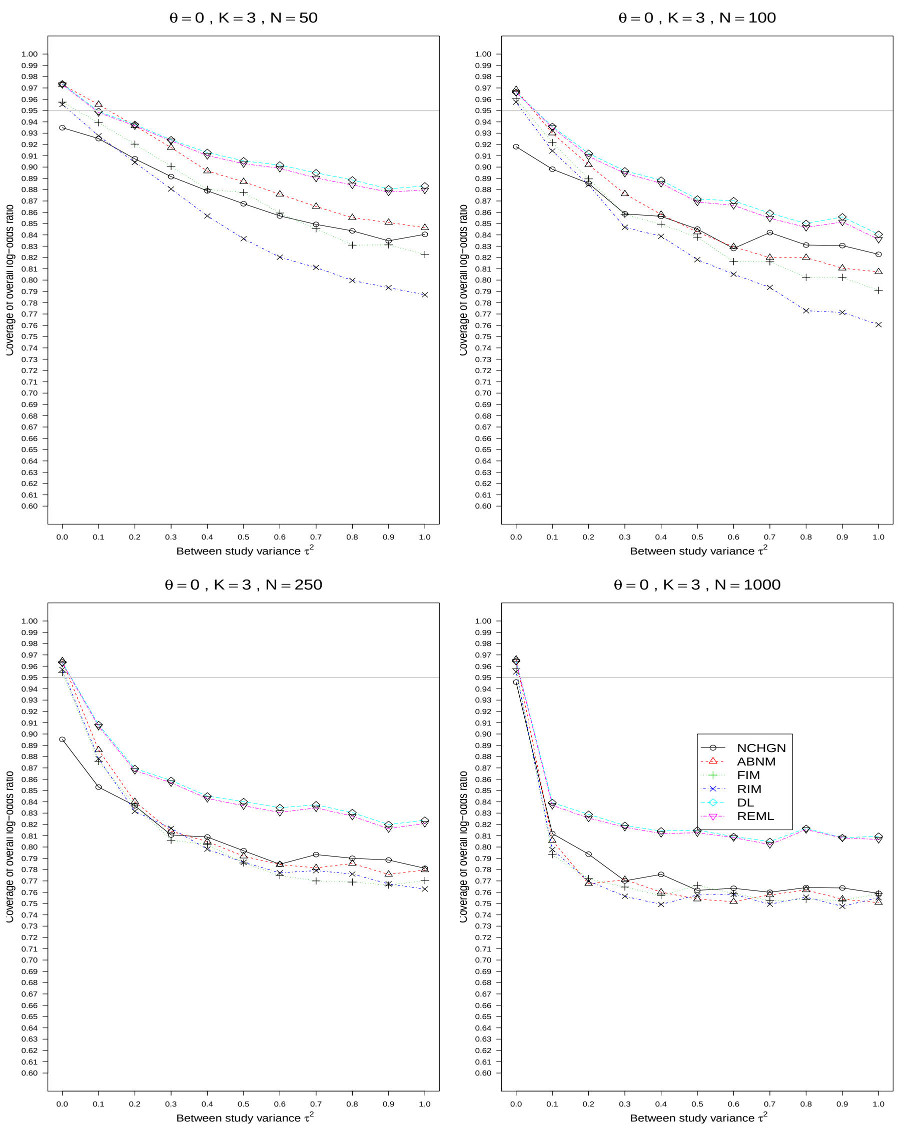

Figure A31: **Estimated coverage of θ in the REM when $K = 3$,$p_{i2} =$ 0.1, $\theta = 0$, $0 \leq \tau^2 \leq 1$ and $n = 50, 100, 250, 1000$.** The coverages are given at the nominal 95% level. Estimation methods are: pluses - unconditional generalized linear mixed-effects model with fixed study effects (FIM), crosses - unconditional generalized linear mixed-effects model with random study effects (RIM), circles - a conditional generalized linear mixed-effects model with exact likelihood (NCHGN), triangles - a conditional generalized linear mixed-effects model with approximate likelihood (ABNM), rhombs - DerSimonian and Laird method (DL) and reverse triangles - restricted maximum likelihood method (REML). Light grey line at 0.95.

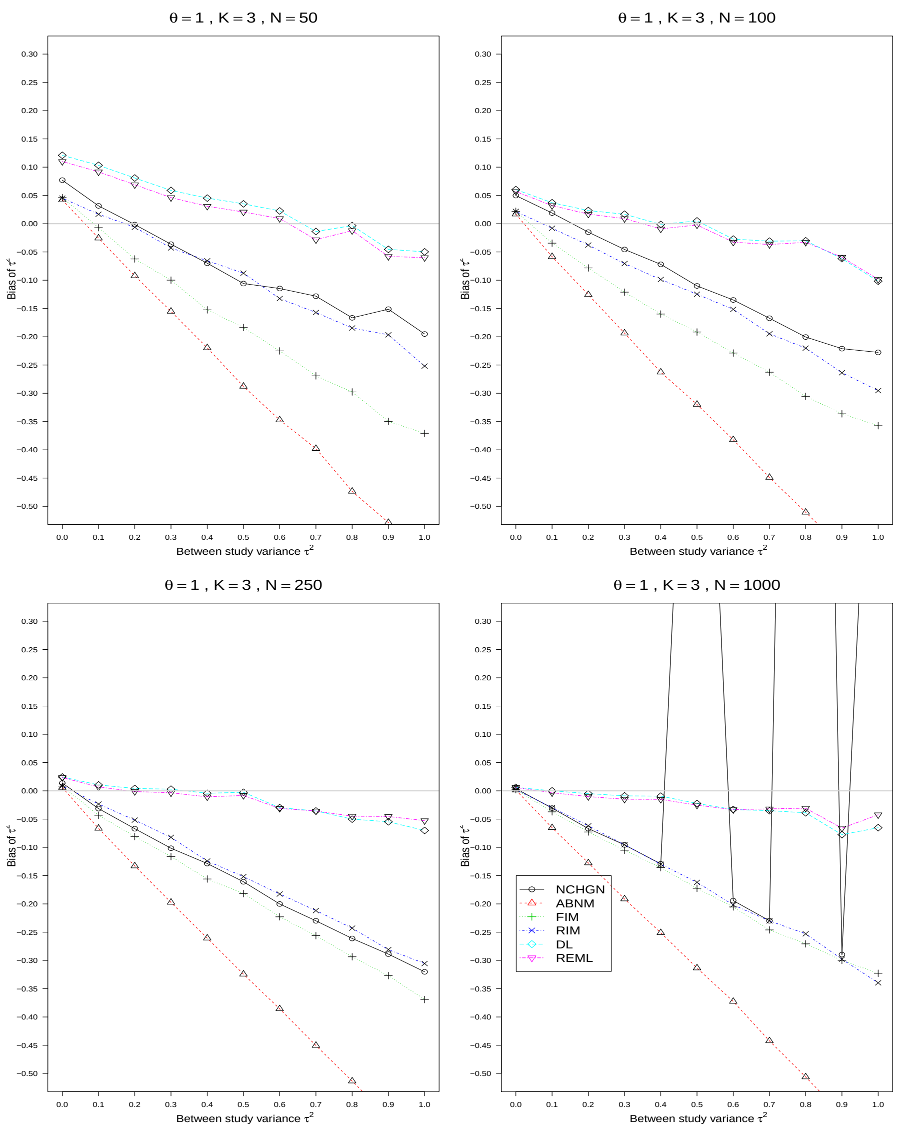

Figure A32: **Bias of τ^2 in the REM when $K = 3, p_{i2} = 0.1$, $\theta = 1$, $0 \leq \tau^2 \leq 1$ and $n = 50, 100, 250, 1000$.** Estimation methods are: pluses - unconditional generalized linear mixed-effects model with fixed study effects (FIM), crosses - unconditional generalized linear mixed-effects model with random study effects (RIM), circles - a conditional generalized linear mixed-effects model with exact likelihood (NCHGN), triangles - a conditional generalized linear mixed-effects model with approximate likelihood (ABNM), rhombs - DerSimonian and Laird method (DL) and reverse triangles - restricted maximum likelihood method (REML). Light grey line at 0.

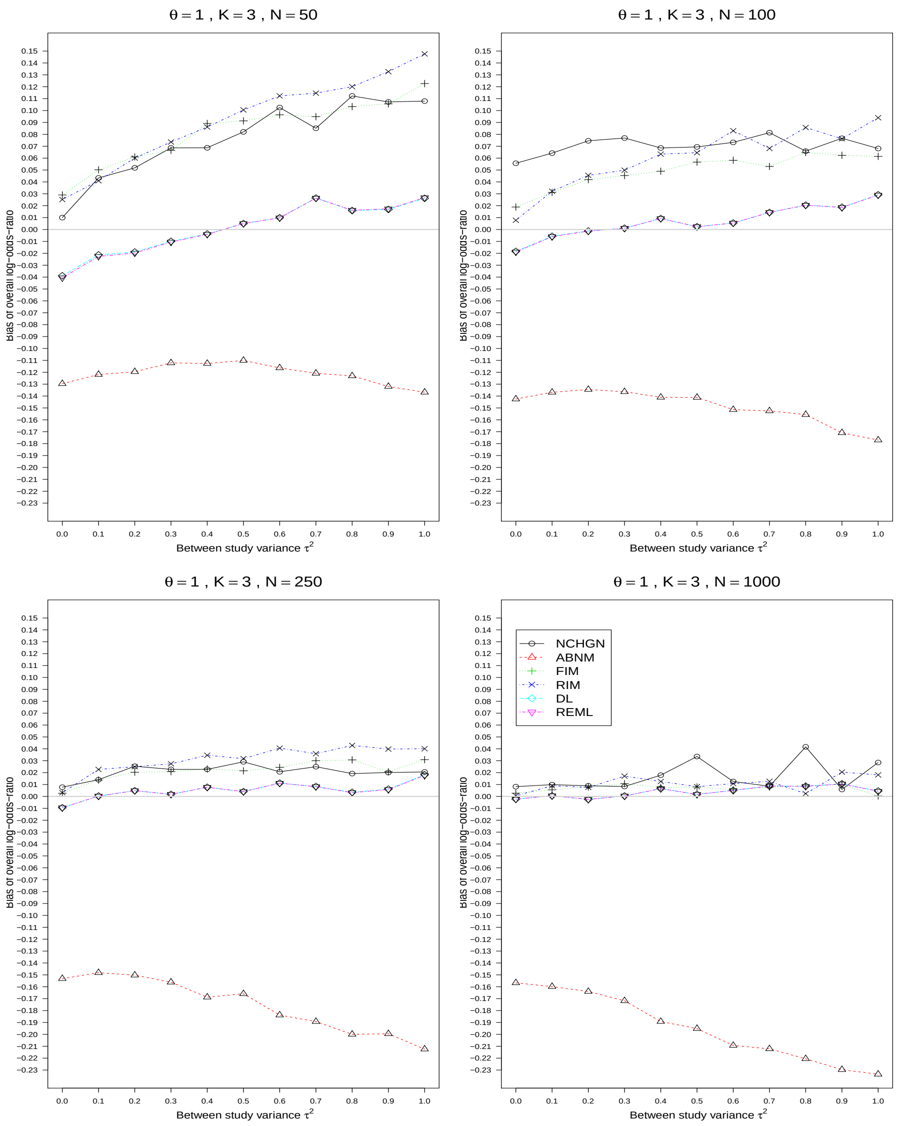

Figure A33: **Bias of θ in the REM when $K = 3$, $p_{i2} = 0.1$, $\theta = 1$, $0 \le \tau^2 \le 1$ and $n = 50, 100, 250, 1000$.** Estimation methods are: pluses - unconditional generalized linear mixed-effects model with fixed study effects (FIM), crosses - unconditional generalized linear mixed-effects model with random study effects (RIM), circles - a conditional generalized linear mixed-effects model with exact likelihood (NCHGN), triangles - a conditional generalized linear mixed-effects model with approximate likelihood (ABNM), rhombs - DerSimonian and Laird method (DL) and reverse triangles - restricted maximum likelihood method (REML). Light grey line at 0.

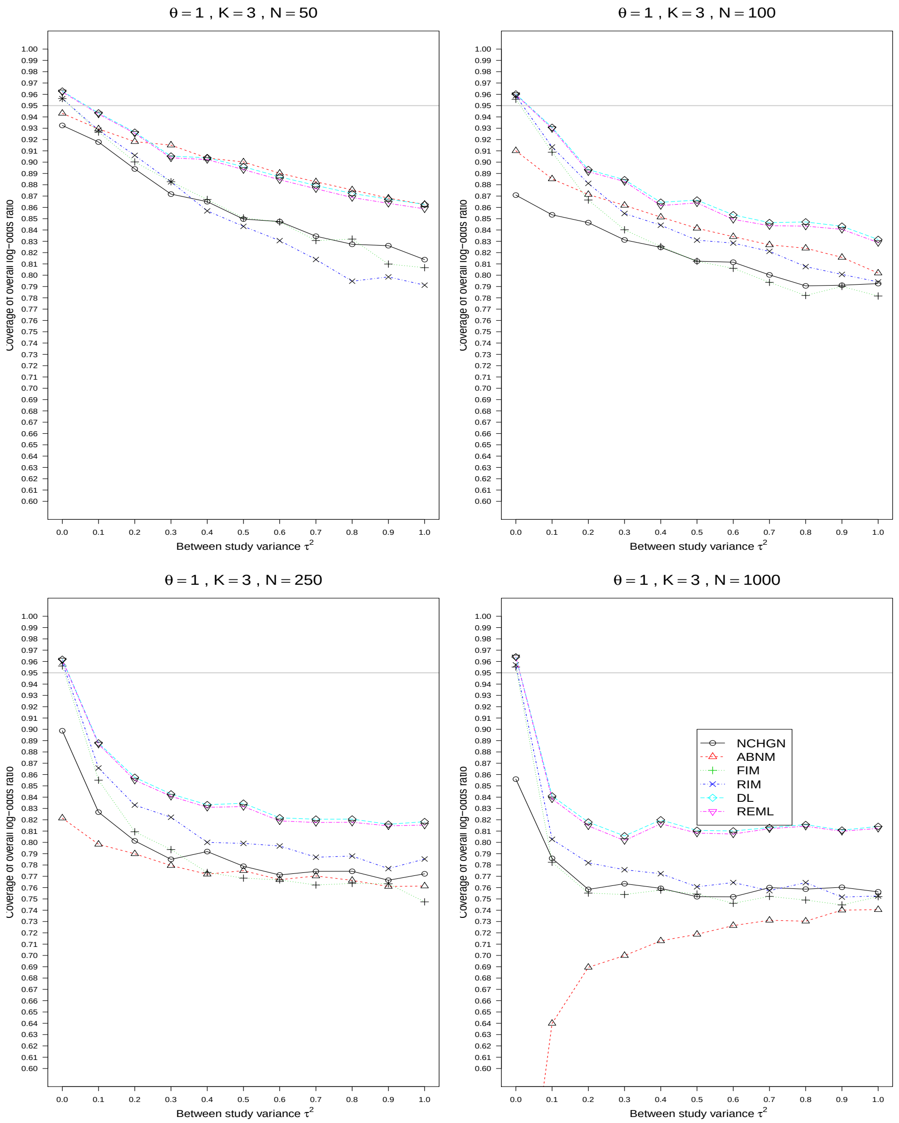

Figure A34: **Estimated coverage of θ in the REM when $K = 3$,$p_{i2} = 0.1$, $\theta = 1$, $0 \leq \tau^2 \leq 1$ and $n = 50, 100, 250, 1000$.** The coverages are given at the nominal 95% level. Estimation methods are: pluses - unconditional generalized linear mixed-effects model with fixed study effects (FIM), crosses - unconditional generalized linear mixed-effects model with random study effects (RIM), circles - a conditional generalized linear mixed-effects model with exact likelihood (NCHGN), triangles - a conditional generalized linear mixed-effects model with approximate likelihood (ABNM), rhombs - DerSimonian and Laird method (DL) and reverse triangles - restricted maximum likelihood method (REML). Light grey line at 0.95.
