# Supplementary material for: Meta-analysis of binary outcomes via generalized linear mixed models: a simulation study
Source: BMC Med Res Methodol. 2018 Jul 4;18:70. doi: 10.1186/s12874-018-0531-9 (PMC6032567; doi:10.1186/s12874-018-0531-9)
Supplement: Supplementary file 4 — - Simulation results for comparison of dFNCHypergeo and dnoncenhypergeom in a conditional GLMM with exact likelihood. This file provides results of simulations with two methods for fitting the non-central hypergeometric distribution in R (PDF 147 kb) [file 12874_2018_531_MOESM4_ESM.pdf]

# Additional file 4 - Simulation results for comparison of dFNCHypergeo and dnoncenhypergeom in a conditional GLMM with exact likelihood

Figures A1-A3 show the results for bias of estimation of between-study variance $\tau^2$, bias of estimates of overall effect measure $\hat{\theta}$ and estimated coverage of overall effect measure $\hat{\theta}$ in the random-effects model.

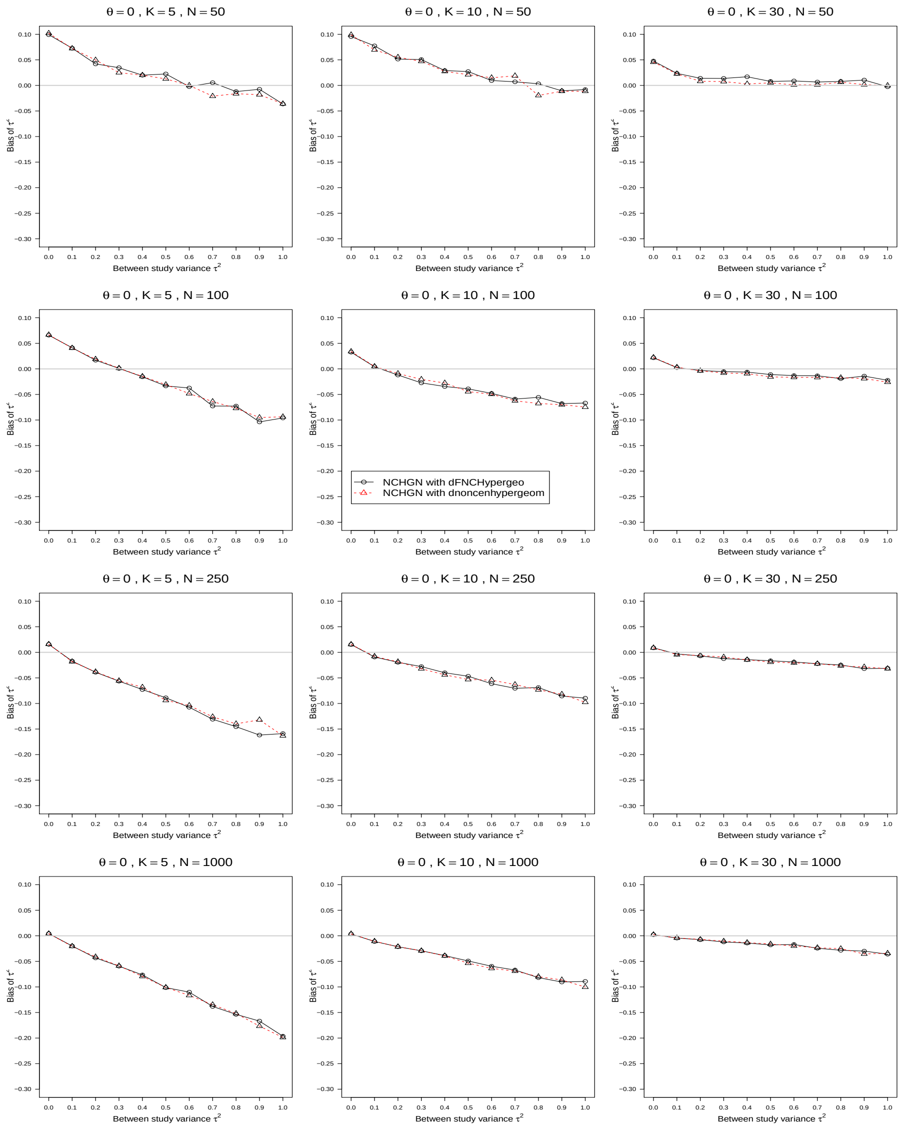


Figure A1: **Bias of $\tau^2$ in the REM when $p_{i2} = 0.1$, $\theta = 0$ , $0 \le \tau^2 \le 1$ and $n = 50, 100, 250, 100.$** Estimation methods are: circles - a conditional GLMM with exact likelihood NCHGN with dFNCHypergeo, and triangles - a conditional GLMM with exact likelihood NCHGN with dnoncenhypergeom. Light grey line at 0.

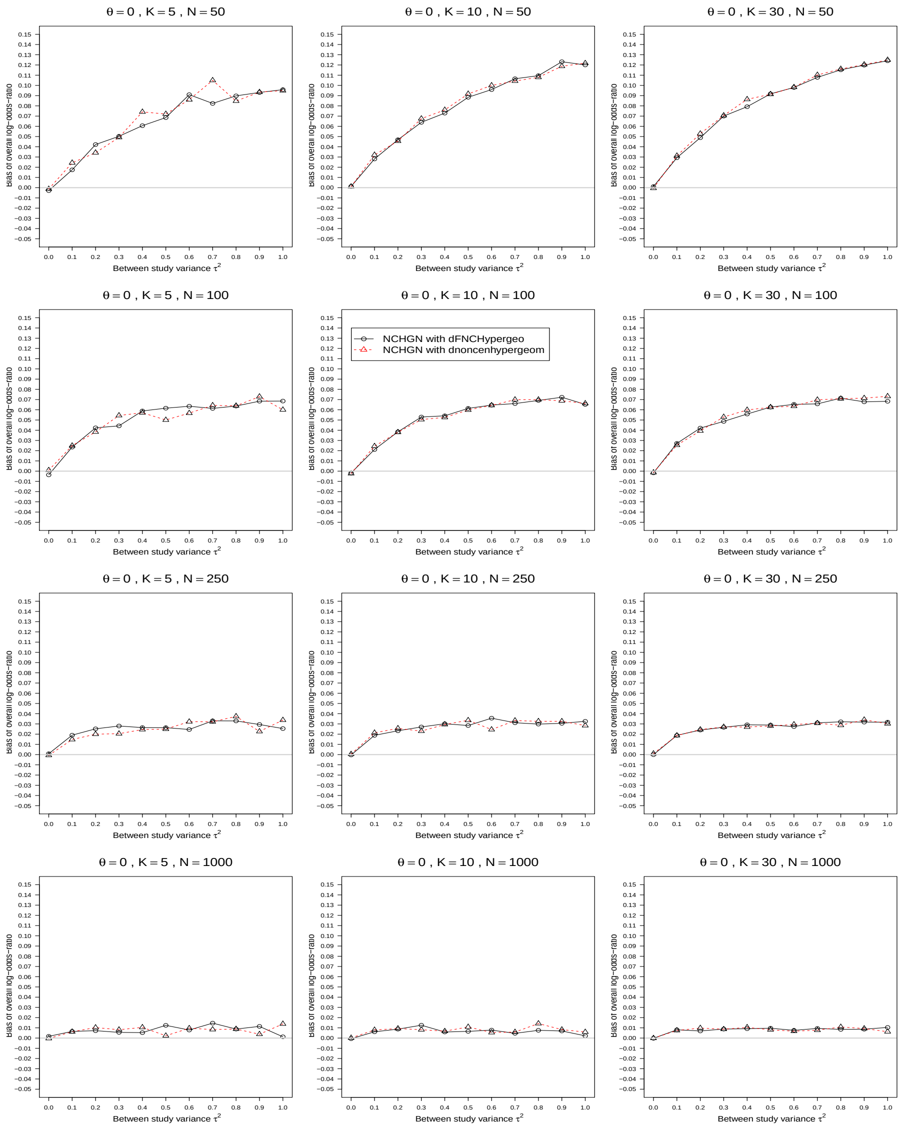


Figure A2: **Bias of $\theta$ in the REM when $p_{i2} = 0.1$, $\theta = 0$ , $0 \leq \tau^2 \leq 1$ and $n = 50, 100, 250, 100$.** Estimation methods are: circles - a conditional GLMM with exact likelihood NCHGN with dFNCHypergeo, and triangles - a conditional GLMM with exact likelihood NCHGN with dnoncenhypergeom. Light grey line at 0.

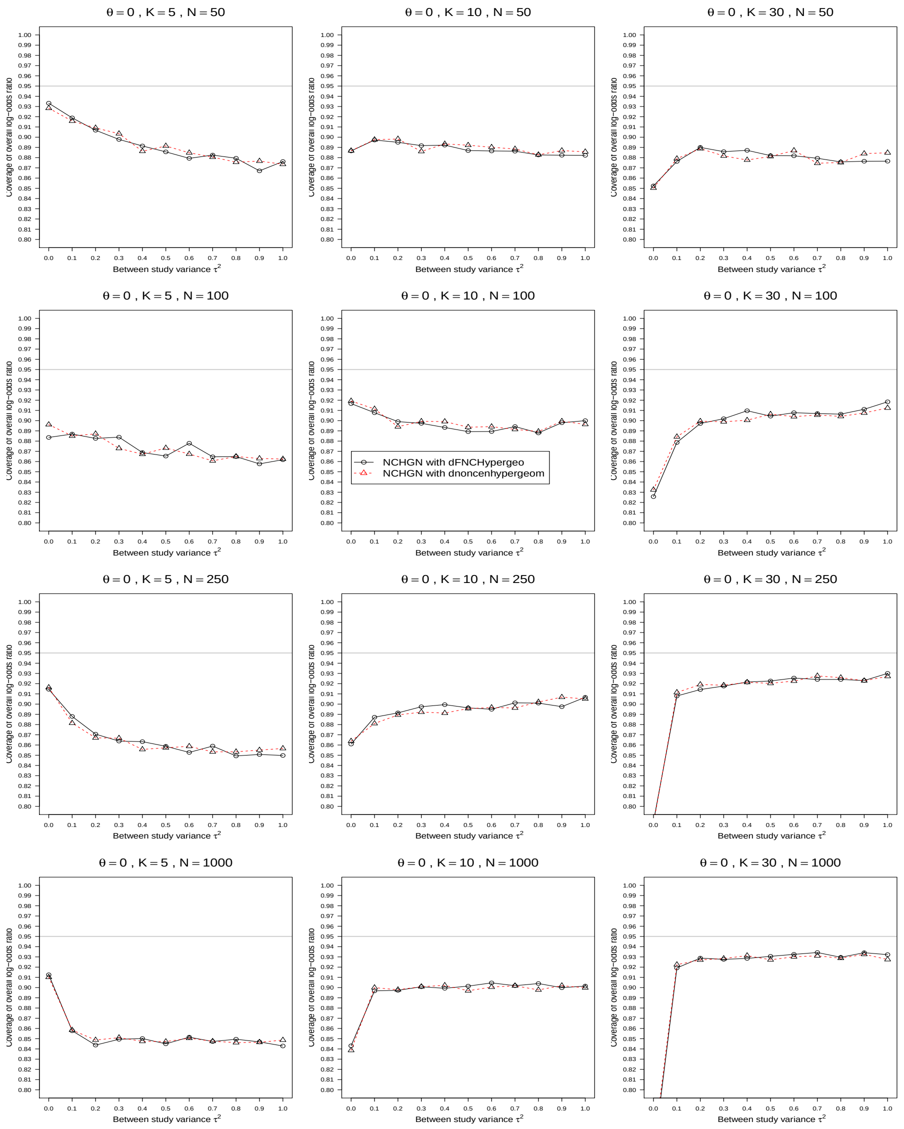


Figure A3: **Estimated coverage of $\theta$ in the REM when $p_{i2} = 0.1$, $\theta = 0$ , $0 \le \tau^2 \le 1$ and $n = 50, 100, 250, 100$.** The coverages are given at the nominal 95% level. Estimation methods are: circles - a conditional GLMM with exact likelihood NCHGN with dFNCHypergeo, and triangles - a conditional GLMM with exact likelihood NCHGN with dnoncenhypergeom. Light grey line at 0.95.
