# Supplementary material for: Meta-analysis of binary outcomes via generalized linear mixed models: a simulation study
Source: BMC Med Res Methodol. 2018 Jul 4;18:70. doi: 10.1186/s12874-018-0531-9 (PMC6032567; doi:10.1186/s12874-018-0531-9)
Supplement: Supplementary file 5 — - Computational issues. This file provides Figures for convergence and estimation quality of alternative optimizers. (PDF 232 kb) [file 12874_2018_531_MOESM5_ESM.pdf]

Additional file 5 - Computational issues

Figures A19-A28 show the results for convergence and estimation quality of alternative optimizers.

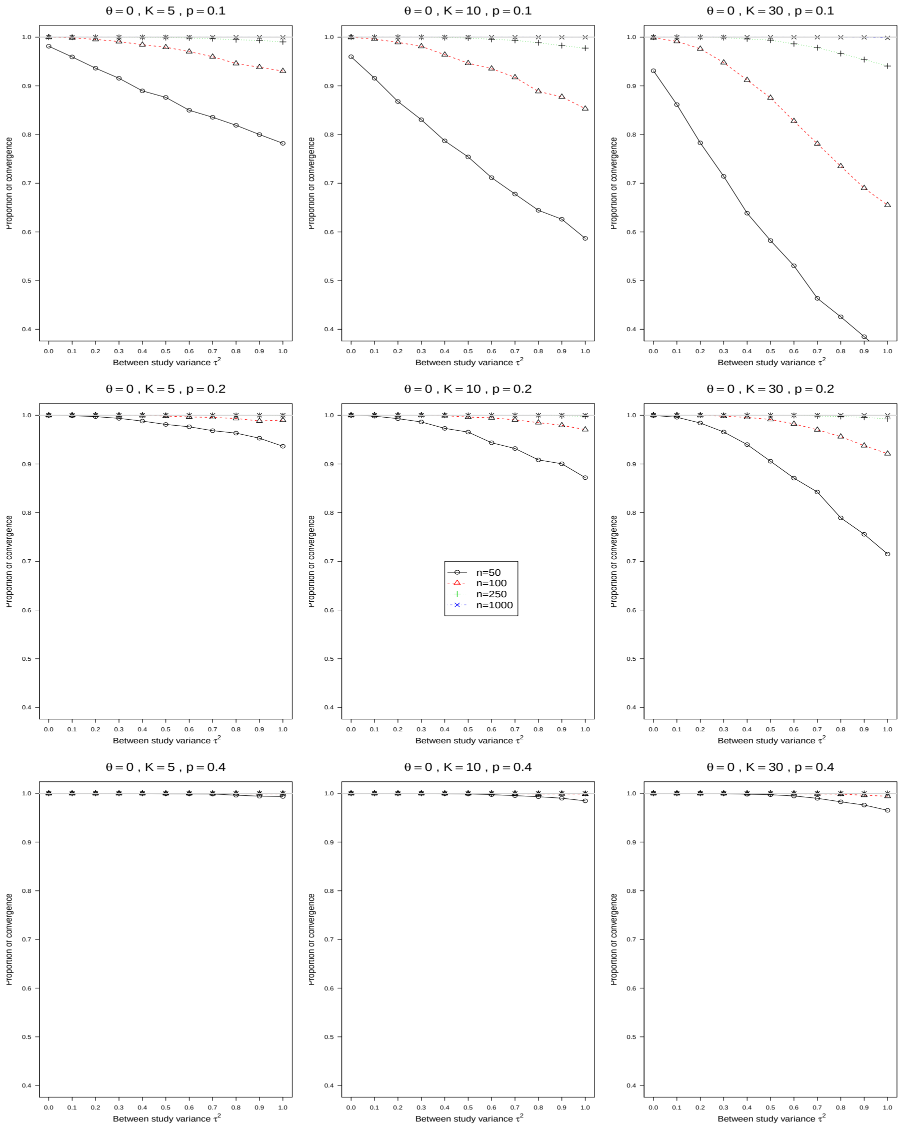

Figure A19: **Proportion of convergence in the random intercept generalized linear mixed-effects model.** The proportions of convergence are for $p_{i2} = 0.1$, $p_{i2} = 0.2$, $p_{i2} = 0.4$, $\theta = 0$, and $0 \leq \tau^2 \leq 1$ for sample sizes $n = 50, 100, 250, 100$ in each arm

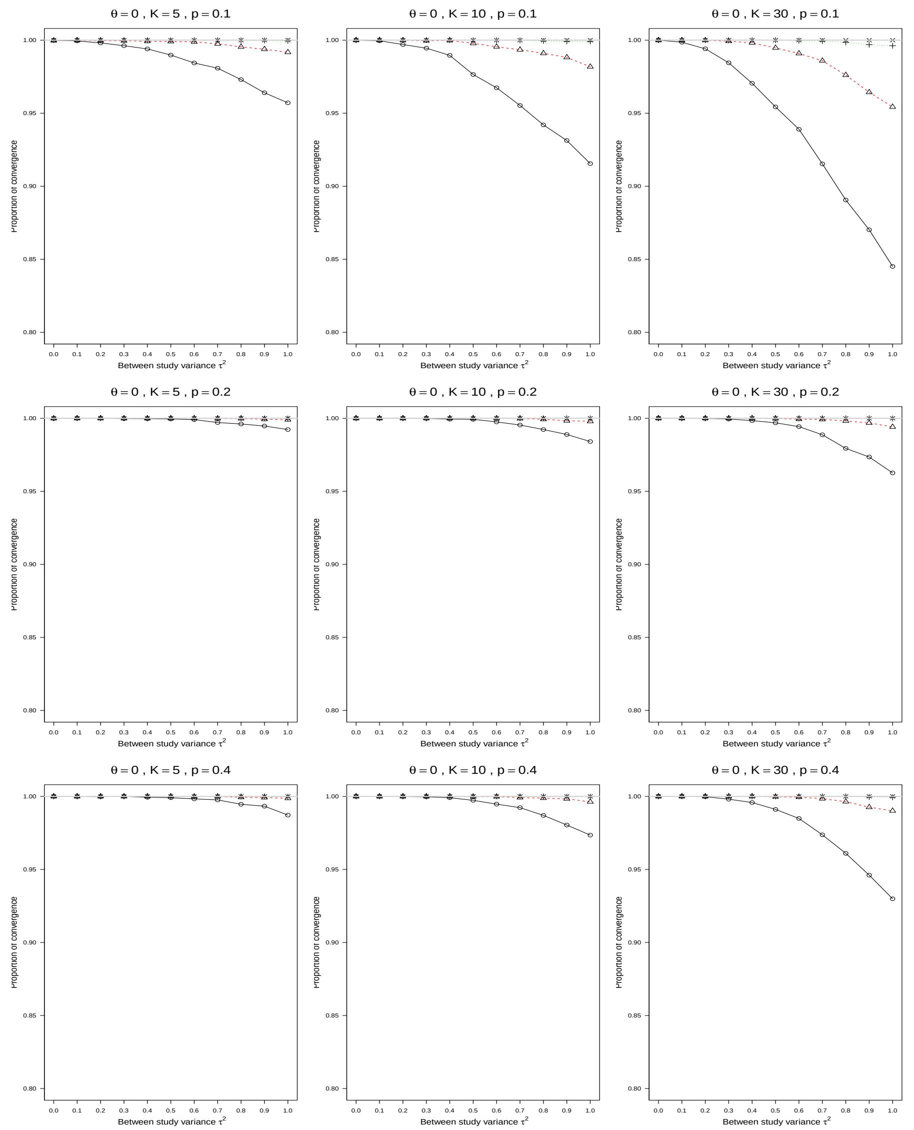

Figure A20: **Proportion of convergence in the random intercept generalized linear mixed-effects model.** The proportions of convergence are for $p_{i2} = 0.1$, $p_{i2} = 0.2$, $p_{i2} = 0.4$, $\theta = 1$, and $0 \leq \tau^2 \leq 1$ for sample sizes $n = 50, 100, 250, 100$ in each arm

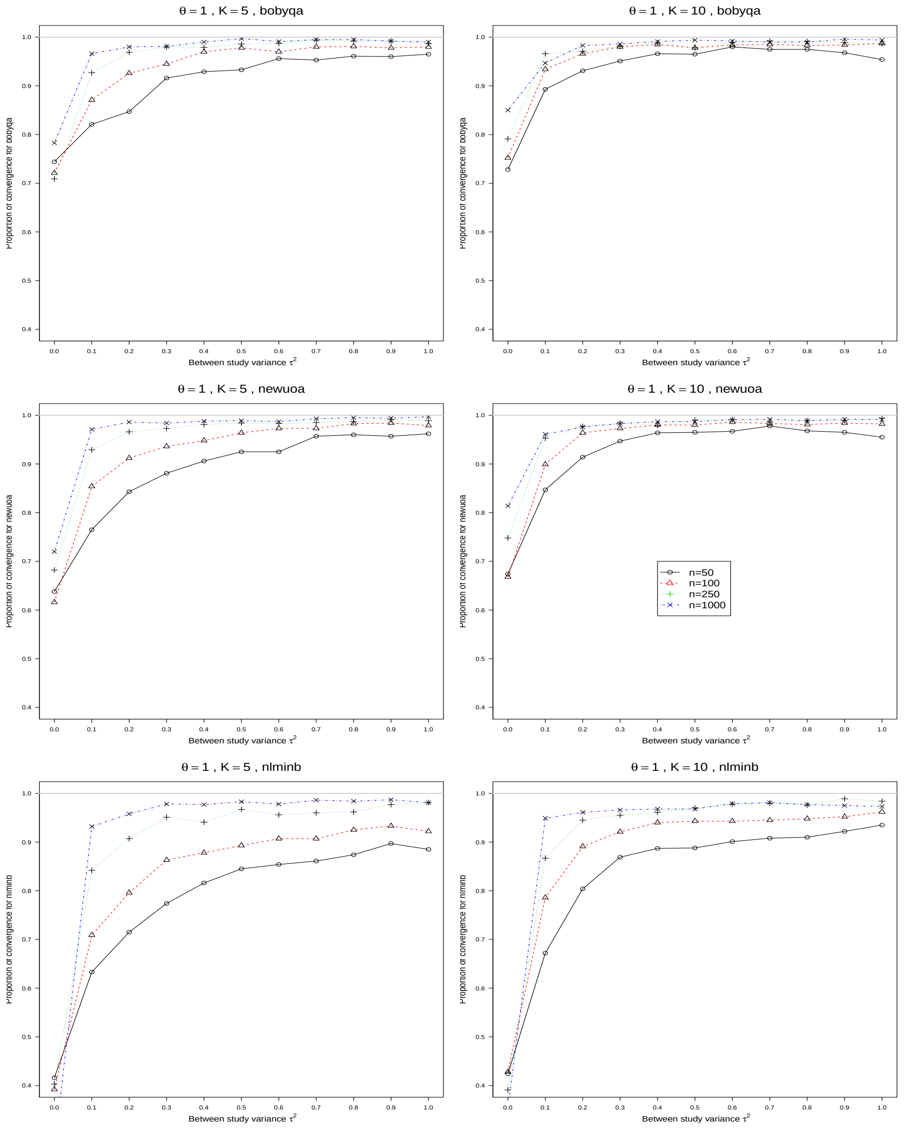

Figure A21: **Proportion of convergence in the conditional generalized linear mixed-effects model with exact likelihood NCHGN.** The proportions of convergence are for different optimizers and $p_{i2} = 0.4$, $\theta = 1$, $0 \leq \tau^2 \leq 1$ and sample sizes $n = 50, 100, 250, 1000$ in each arm

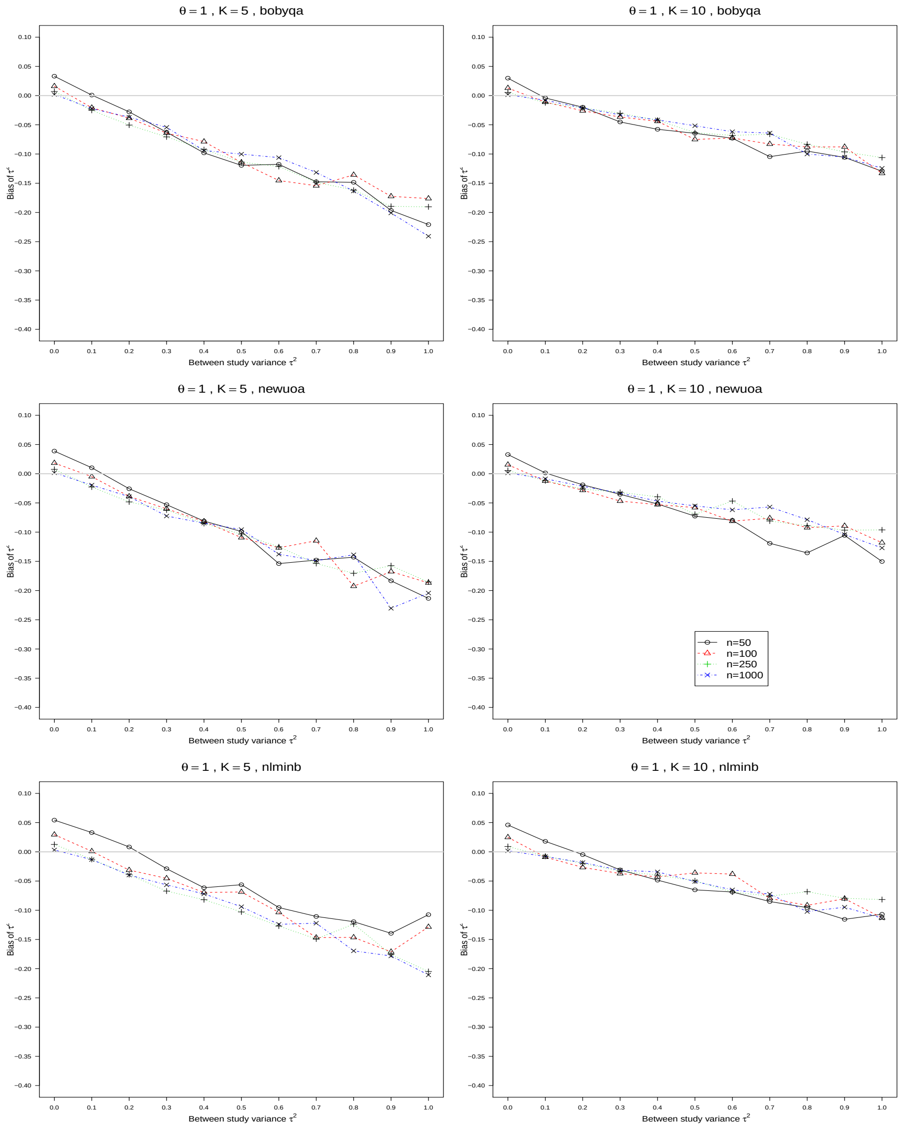

Figure A22: **Bias in estimation of τ^2 in the conditional generalized linear mixed-effects model with exact likelihood NCHGN.** The biases are for different optimizers and $p_{i2} = 0.4$, $\theta = 1$, $0 \leq \tau^2 \leq 1$ and sample sizes $n = 50, 100, 250, 1000$ in each arm

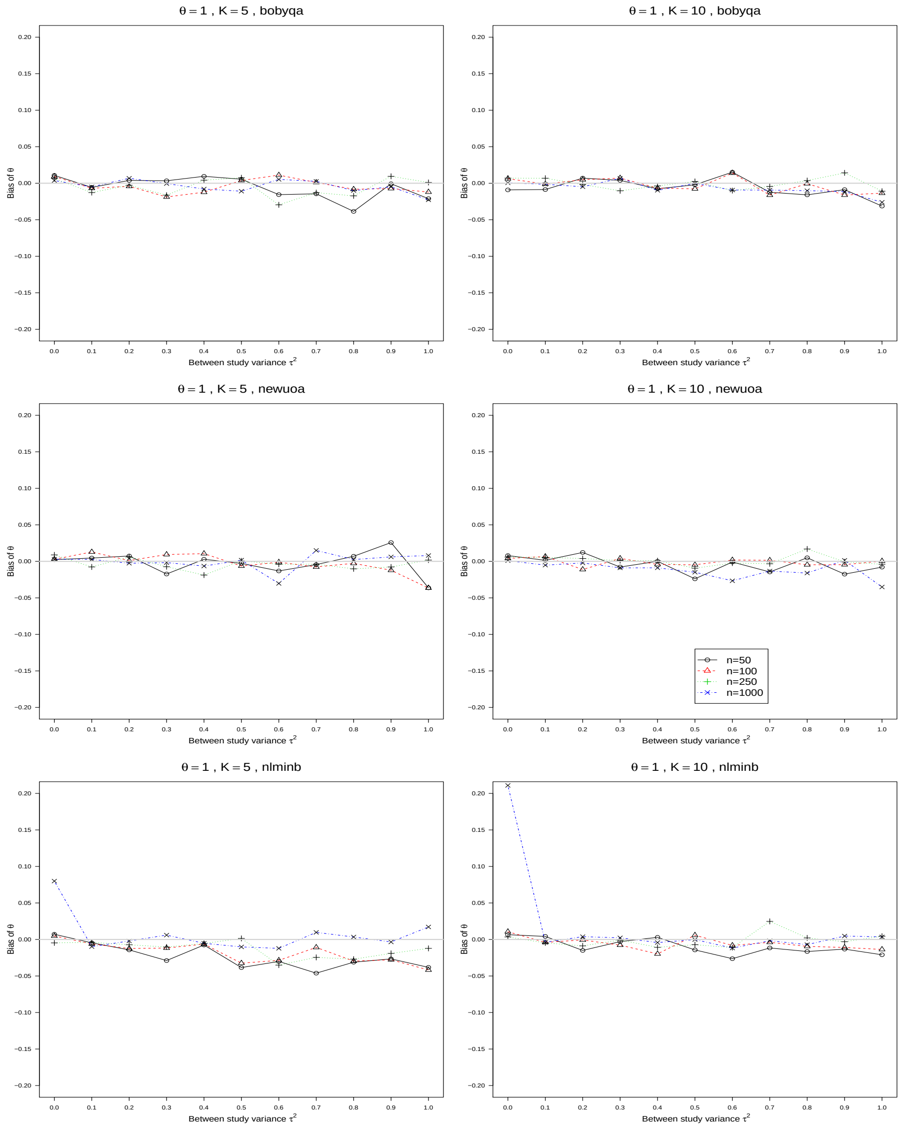

Figure A23: **Bias in estimation of θ in the conditional generalized linear mixed-effects model with exact likelihood NCHGN.** The biases are for different optimizers and $p_{i2} = 0.4$, $\theta = 1$, $0 \le \tau^2 \le 1$ and sample sizes $n = 50, 100, 250, 1000$ in each arm

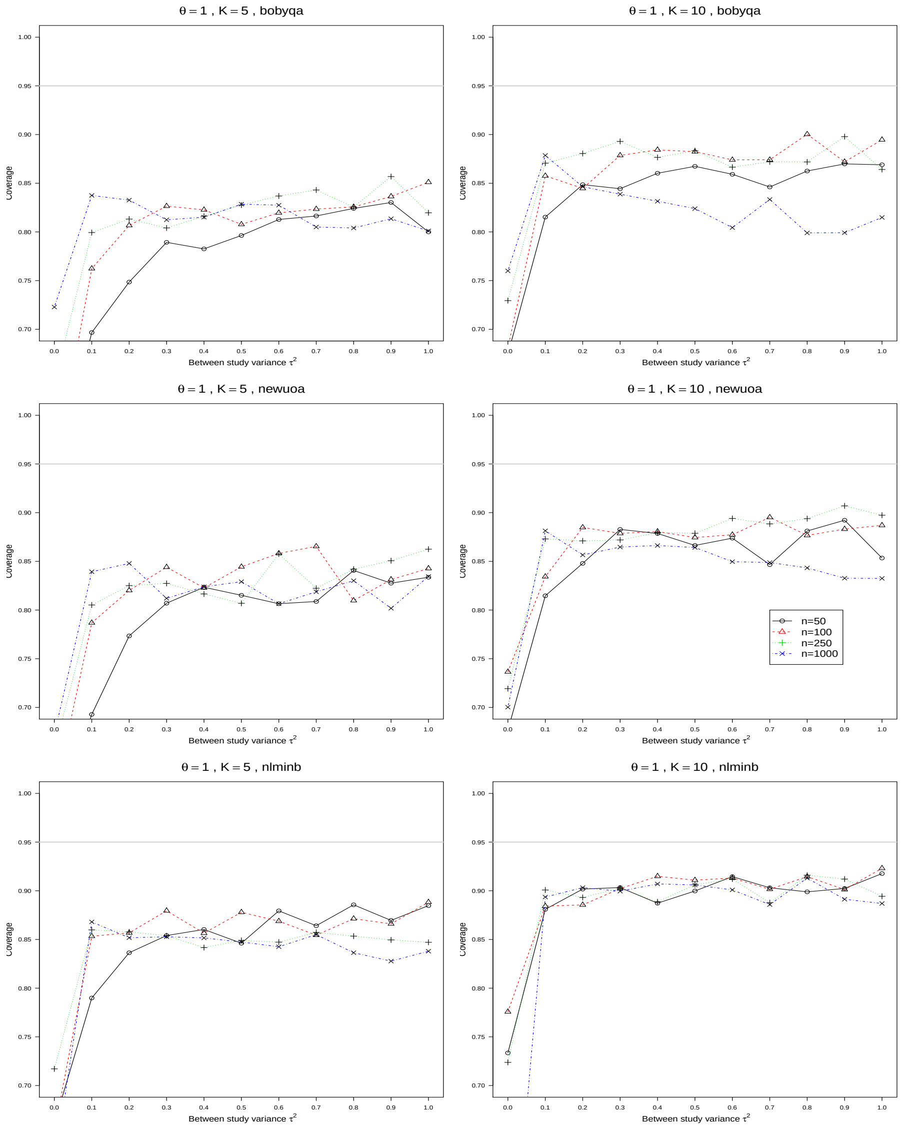

Figure A24: **Estimated coverage of θ in the conditional generalized linear mixed-effects model with exact likelihood**. The coverages are given at the nominal 95% level and for different optimizers with $p_{i2} = 0.4$, $\theta = 1$, $0 \leq \tau^2 \leq 1$ and sample sizes $n = 50, 100, 250, 1000$ in each arm

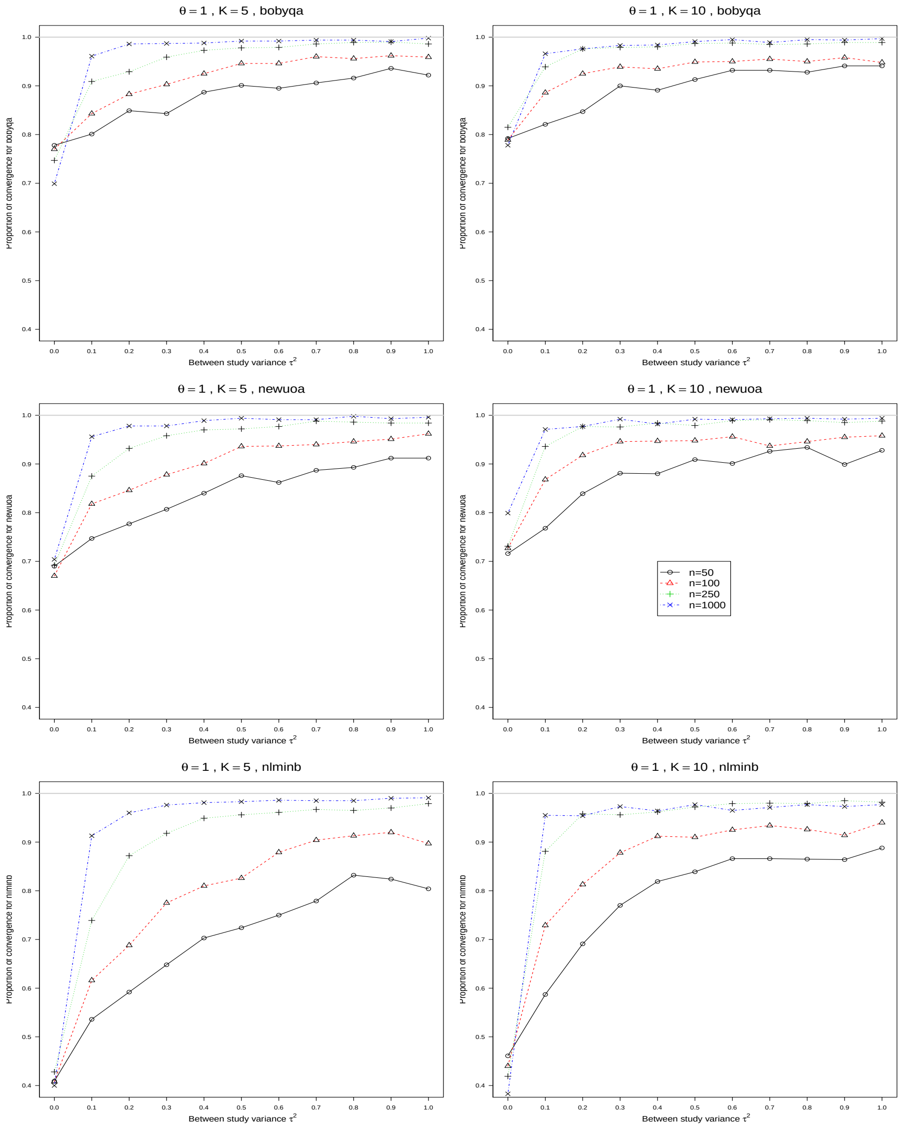

Figure A25: **Proportion of convergence in the conditional generalized linear mixed-effects model with exact likelihood NCHGN.** The proportions of convergence are for different optimizers for $p_{i2} = 0.1$, $\theta = 1$, $0 \leq \tau^2 \leq 1$ and sample sizes $n = 50, 100, 250, 1000$ in each arm

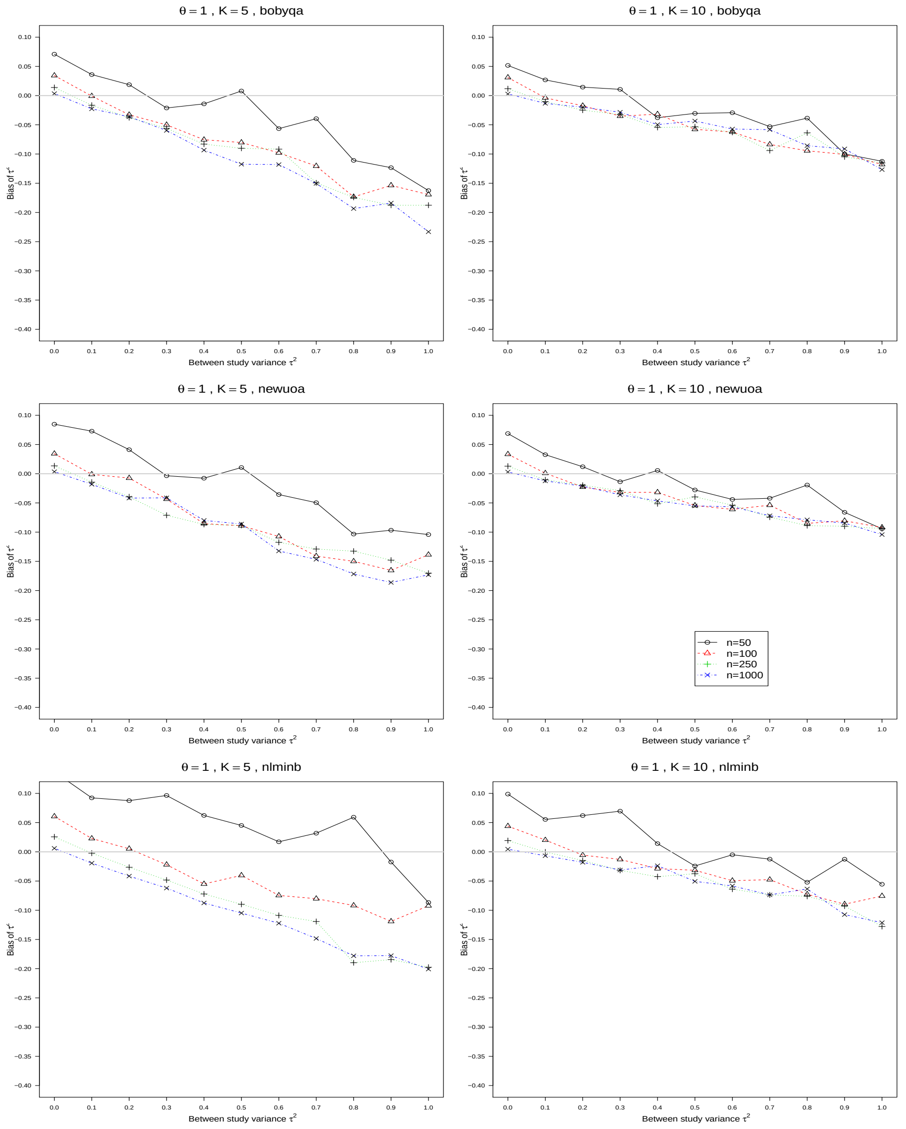

Figure A26: **Bias of τ^2 in the conditional generalized linear mixed-effects model with exact likelihood NCHGN.** The biases are for different optimizers and $p_{i2} = 0.1$, $\theta = 1$, $0 \leq \tau^2 \leq 1$ and sample sizes $n = 50, 100, 250, 1000$ in each arm

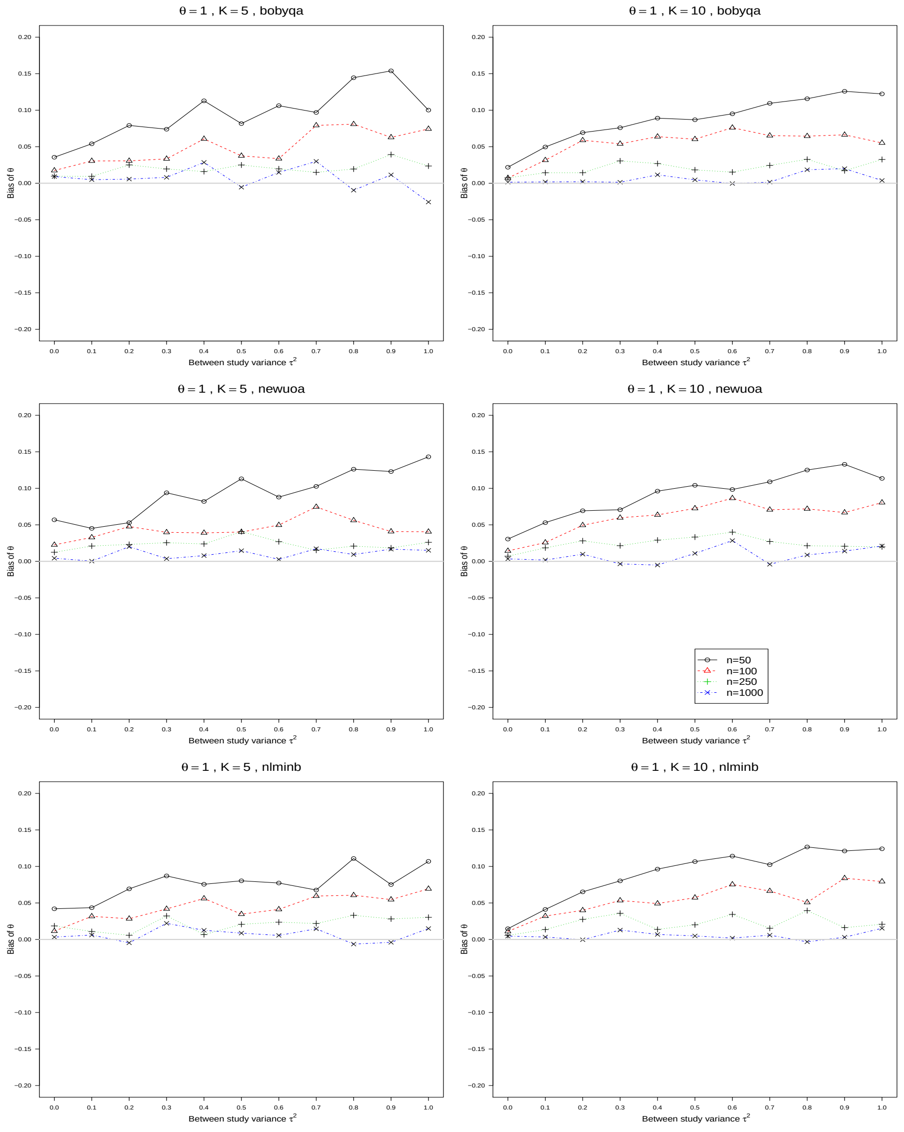

Figure A27: **Bias of θ in the conditional generalized linear mixed-effects model with exact likelihood NCHGN.** The biases are for different optimizers and $p_{i2} = 0.1$, $\theta = 1$, $0 \le \tau^2 \le 1$ and sample sizes $n = 50, 100, 250, 1000$ in each arm

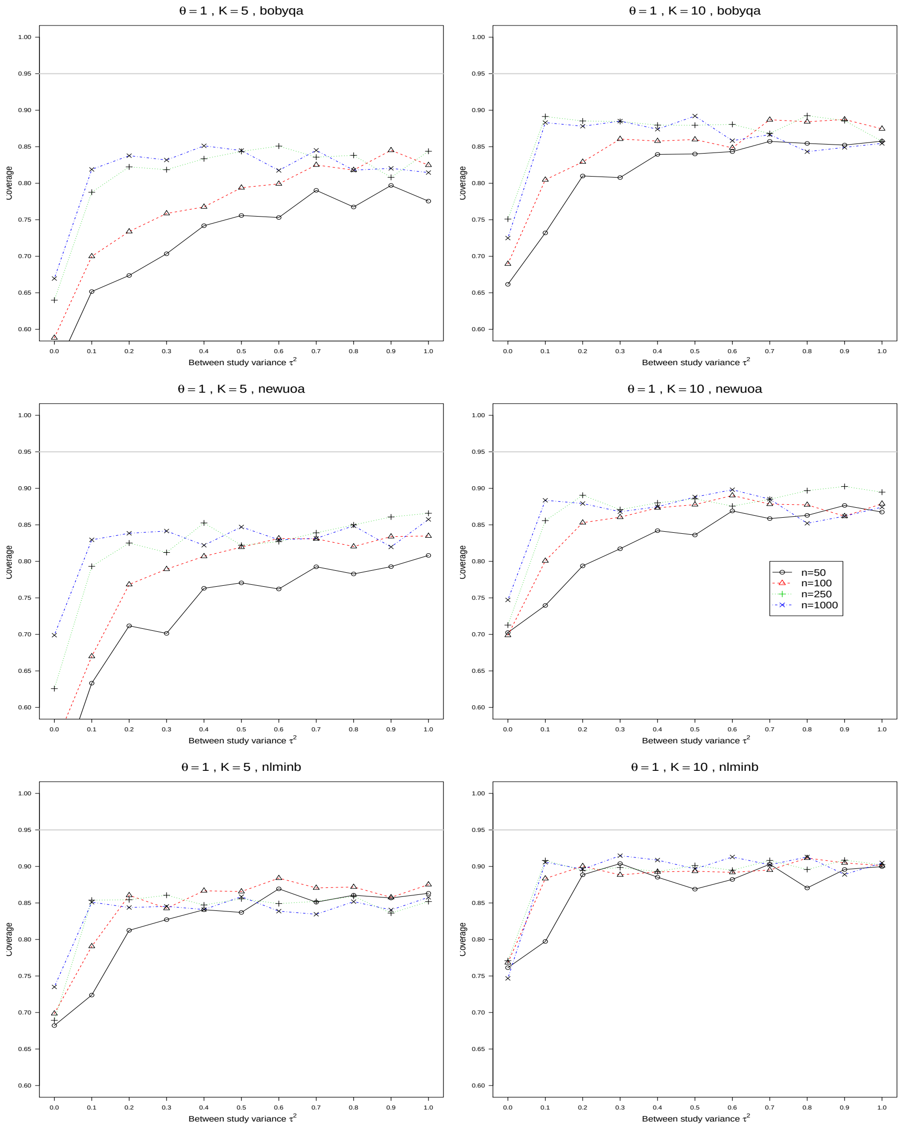

Figure A28: **Estimated coverage of θ in the conditional generalized linear mixed-effects model with exact likelihood**. The coverages are given at the nominal 95% level and for different optimizers with $p_{i2} = 0.1$, $\theta = 1$, $0 \leq \tau^2 \leq 1$ and sample sizes $n = 50, 100, 250, 1000$ in each arm
