# Supplementary material for: Meta-analysis of binary outcomes via generalized linear mixed models: a simulation study
Source: BMC Med Res Methodol. 2018 Jul 4;18:70. doi: 10.1186/s12874-018-0531-9 (PMC6032567; doi:10.1186/s12874-018-0531-9)
Supplement: Supplementary file 6 — - R code for GLMM analysis of simulated data in “Computational issues” section. This file provides R code for GLMM analysis of simulated data in “Computational issues” section. (PDF 122 kb) [file 12874_2018_531_MOESM6_ESM.pdf]

Additional file 6 - R code for GLMM analysis of simulated data in Section 3.2

```
# a conditional generalized linear mixed-effects model (exact likelihood)
#with default option for specification of non-central hypergeometric
#distribution-dFNCHypergeo from BiasUrn package
#and optimizer is set by default to "optim"
> ai<-c(726,741,892,630,745)
> bi<-c(274,259,108,370,255)
> ci<-c(401,406,378,415,404)
> di<-c(599,594,622,585,596)
> library(metafor)
Loading required package: Matrix
Loading 'metafor' package (version 1.9-9). For an overview
and introduction to the package please type: help(metafor).
> res1<-rma.glmm(measure="OR", ai=ai, bi=bi, ci=ci, di=di,
+model="CM.EL")
> res1

Random-Effects Model (k = 5; tau^2 estimator: ML)
Model Type: Conditional Model with Exact Likelihood

tau^2 (estimated amount of total heterogeneity): 1169.0647 (SE = 800.2414)
tau (square root of estimated tau^2 value):      34.1916
I^2 (total heterogeneity / total variability):   100.00%
H^2 (total variability / sampling variability):  118176.79

Tests for Heterogeneity:
Wld(df = 4) = 131.6573, p-val < .0001
LRT(df = 4) = 144.1083, p-val < .0001

Model Results:

estimate      se     zval     pval    ci.lb    ci.ub
35.6833   4.4481   8.0221   <.0001  26.9652  44.4014      ***
#####################################################################
# a conditional generalized linear mixed-effects model (exact likelihood)
#with default option for specification of non-central hypergeometric
#distribution-dnoncenhypergeom from from MCMCpack package
#and optimizer is set by default to "optim"
> res2<-rma.glmm(measure="OR", ai=ai, bi=bi, ci=ci, di=di,
+model="CM.EL",control=list(dnchgcalc="dnoncenhypergeom"))
> res2
```

```
Random-Effects Model (k = 5; tau^2 estimator: ML)
Model Type: Conditional Model with Exact Likelihood

tau^2 (estimated amount of total heterogeneity): 1169.0647 (SE = 800.2357)
tau (square root of estimated tau^2 value):      34.1916
I^2 (total heterogeneity / total variability):   100.00%
H^2 (total variability / sampling variability):  118176.79

Tests for Heterogeneity:
Wld(df = 4) = 131.6600, p-val < .0001
LRT(df = 4) = 144.1083, p-val < .0001

Model Results:

estimate      se     zval     pval    ci.lb    ci.ub
35.6833   4.4480   8.0222   <.0001  26.9653  44.4013     ***

# a conditional generalized linear mixed-effects model (exact likelihood)
#with default option for specification of non-central hypergeometric
#distribution-dFNCHypergeo from BiasUrn package
#and optimizer is set by default to "uobyqa"
> res3<-rma.glmm(measure="OR", ai=ai, bi=bi, ci=ci, di=di,
+model="CM.EL", control=list(optimizer="uobyqa"))
> res3

Random-Effects Model (k = 5; tau^2 estimator: ML)
Model Type: Conditional Model with Exact Likelihood

tau^2 (estimated amount of total heterogeneity): 0.3113 (SE = 0.2048)
tau (square root of estimated tau^2 value):      0.5580
I^2 (total heterogeneity / total variability):   96.92%
H^2 (total variability / sampling variability):  32.47

Tests for Heterogeneity:
Wld(df = 4) = 131.6627, p-val < .0001
LRT(df = 4) = 144.1083, p-val < .0001

Model Results:

estimate      se     zval     pval    ci.lb    ci.ub
1.5472    0.2536   6.1015   <.0001   1.0502   2.0442     ***


# a conditional generalized linear mixed-effects model (exact likelihood)
#with default option for specification of non-central hypergeometric
#distribution-dFNCHypergeo from BiasUrn package
```

```
#and optimizer is set by default to "bobyqa"
> res4<-rma.glmm(measure="OR", ai=ai, bi=bi, ci=ci, di=di,
+model="CM.EL", control=list(optimizer="bobyqa"))
> res4
Random-Effects Model (k = 5; tau^2 estimator: ML)
Model Type: Conditional Model with Exact Likelihood

tau^2 (estimated amount of total heterogeneity): 0.3113 (SE = 0.2049)
tau (square root of estimated tau^2 value):      0.5580
I^2 (total heterogeneity / total variability):   96.92%
H^2 (total variability / sampling variability):  32.47

Tests for Heterogeneity:
Wld(df = 4) = 131.6625, p-val < .0001
LRT(df = 4) = 144.1083, p-val < .0001

Model Results:

estimate      se      zval     pval     ci.lb     ci.ub
1.5472    0.2536    6.1015   <.0001   1.0502    2.0442      ***

# a conditional generalized linear mixed-effects model (exact likelihood)
#with default option for specification of non-central hypergeometric
#distribution-dFNCHypergeo from BiasUrn package
#and optimizer is set by default to "newuoa"
res5<-rma.glmm(measure="OR", ai=ai, bi=bi, ci=ci, di=di,
+model="CM.EL", control=list(optimizer="newuoa"))

> res5

Random-Effects Model (k = 5; tau^2 estimator: ML)
Model Type: Conditional Model with Exact Likelihood

tau^2 (estimated amount of total heterogeneity): 0.3113 (SE = 0.2049)
tau (square root of estimated tau^2 value):      0.5580
I^2 (total heterogeneity / total variability):   96.92%
H^2 (total variability / sampling variability):  32.47

Tests for Heterogeneity:
Wld(df = 4) = 131.6643, p-val < .0001
LRT(df = 4) = 144.1083, p-val < .0001

Model Results:

estimate      se      zval     pval     ci.lb     ci.ub
1.5472    0.2536    6.1015   <.0001   1.0502    2.0442      ***
```

```
########################################################################
########################################################################
########################################################################
#a conditional generalized linear mixed-effects model (approximate likelihood)
>  res6<-rma.glmm(measure="OR", ai=ai, bi=bi, ci=ci, di=di, model="CM.AL")
> res6

Random-Effects Model (k = 5; tau^2 estimator: ML)
Model Type: Conditional Model with Approximate Likelihood

tau^2 (estimated amount of total heterogeneity): 0.0160
tau (square root of estimated tau^2 value):      0.1264
I^2 (total heterogeneity / total variability):   61.76%
H^2 (total variability / sampling variability):  2.61

Tests for Heterogeneity:
Wld(df = 4) = 25.6608, p-val < .0001
LRT(df = 4) = 25.9627, p-val < .0001

Model Results:

estimate      se     zval     pval    ci.lb    ci.ub
0.6177    0.0630   9.8092   <.0001   0.4943   0.7411      ***

#an unconditional generalized linear mixed-effects model
#with random study effects
>  res7<-rma.glmm(measure="OR", ai=ai, bi=bi, ci=ci, di=di, model="UM.RS")
Warning message:
In rma.glmm(measure = "OR", ai = ai, bi = bi, ci = ci, di = di,
:
Currently not possible to fit RE/ME model='UM.RS' with nAGQ > 1. nAGQ automatically
> res7

Random-Effects Model (k = 5; tau^2 estimator: ML)
Model Type: Unconditional Model with Random Study Effects

tau^2 (estimated amount of total heterogeneity): 0.3021
tau (square root of estimated tau^2 value):      0.5497
I^2 (total heterogeneity / total variability):   96.83%
H^2 (total variability / sampling variability):  31.54

sigma^2 (estimated amount of study level variability): 0.0499
sigma (square root of estimated sigma^2 value):        0.2234

Tests for Heterogeneity:
```

```
Wld(df = 4) = 171.3118, p-val < .0001
LRT(df = 4) = 158.5442, p-val < .0001

Model Results:

estimate    se      zval     pval     ci.lb    ci.ub
1.5446    0.2499  6.1806   <.0001   1.0548   2.0344    ***

#an unconditional generalized linear mixed-effects model
#with fixed study effects
>  res8<-rma.glmm(measure="OR", ai=ai, bi=bi, ci=ci, di=di, model="UM.FS")
> res8

Random-Effects Model (k = 5; tau^2 estimator: ML)
Model Type: Unconditional Model with Fixed Study Effects

tau^2 (estimated amount of total heterogeneity): 0.3106
tau (square root of estimated tau^2 value):      0.5573
I^2 (total heterogeneity / total variability):   96.91%
H^2 (total variability / sampling variability):  32.40

Tests for Heterogeneity:
Wld(df = 4) = 131.7382, p-val < .0001
LRT(df = 4) = 144.1967, p-val < .0001

Model Results:

estimate    se      zval     pval     ci.lb    ci.ub
1.5477    0.2533  6.1104   <.0001   1.0513   2.0442    ***

##########################################################################
##################Standard REM with REML method#########################
##########################################################################
> res9<-rma(measure="OR", ai=ai, bi=bi, ci=ci, di=di, method="REML")
> res9

Random-Effects Model (k = 5; tau^2 estimator: REML)

tau^2 (estimated amount of total heterogeneity): 0.3921 (SE = 0.2845)
tau (square root of estimated tau^2 value):      0.6262
I^2 (total heterogeneity / total variability):   97.54%
H^2 (total variability / sampling variability):  40.64

Test for Heterogeneity:
Q(df = 4) = 131.7382, p-val < .0001
```

```
Model Results:

estimate      se     zval     pval    ci.lb    ci.ub
1.5476    0.2837   5.4557   <.0001   0.9916   2.1035     ***

##########################################################################
###################Standard REM with DL method############################
##########################################################################
> res10<-rma(measure="OR", ai=ai, bi=bi, ci=ci, di=di, method="DL")
> res10

Random-Effects Model (k = 5; tau^2 estimator: DL)

tau^2 (estimated amount of total heterogeneity): 0.3159 (SE = 0.2331)
tau (square root of estimated tau^2 value):      0.5621
I^2 (total heterogeneity / total variability):   96.96%
H^2 (total variability / sampling variability):  32.93

Test for Heterogeneity:
Q(df = 4) = 131.7382, p-val < .0001

Model Results:

estimate      se     zval     pval    ci.lb    ci.ub
1.5469    0.2554   6.0570   <.0001   1.0463   2.0474     ***


res11<-rma(measure="OR", ai=ai, bi=bi, ci=ci, di=di, method="FE")

Fixed-Effects Model (k = 5)

Test for Heterogeneity:
Q(df = 4) = 131.7382, p-val < .0001

Model Results:

estimate      se     zval     pval    ci.lb    ci.ub
1.4540    0.0443  32.8080   <.0001   1.3671   1.5409     ***
```
