# Supplementary material for: Meta-analysis of binary outcomes via generalized linear mixed models: a simulation study
Source: BMC Med Res Methodol. 2018 Jul 4;18:70. doi: 10.1186/s12874-018-0531-9 (PMC6032567; doi:10.1186/s12874-018-0531-9)
Supplement: Supplementary file 7 — - R code for an example where the optimizer “uobyqa” hangs. This file provides R code and output for an example where the optimizer “uobyqa” hangs. (PDF 107 kb) [file 12874_2018_531_MOESM7_ESM.pdf]

Additional file 7 - R code for an example where the “uobyqa” hangs

This is an example of a simulated dataset causing hang problems in simulations of a conditional generalized linear mixed-effects model with exact likelihood (NCHGN) with an optimizer “uobyqa” in rma.glmm function of metafor package. Due to hang problems, our simulations for an optimizer “uobyqa” could not continue. We have also tried to fit a conditional generalized linear mixed-effects model with exact likelihood (NCHGN) for the same dataset with different optimizers such as “optim”, “nlminb”,“bobyqa”,“newuoa” . However, all these optimizers have not converged and resulted in errors which are shown below. In addition to GLMM methods, we meta-analysed this simulated dataset with standard DerSimonian and Laird method and restricted maximum likelihood method.

```
> library(metafor)
Loading required package: Matrix
Loading 'metafor' package (version 1.9-9). For an overview
and introduction to the package please type: help(metafor).
ai<-c(35,33,31,35,35)
bi<-c(15,17,19,15,15)
ci<-c(17,17,21,21,22)
di<-c(33,33,29,29,28)
# a conditional generalized linear mixed-effects model (exact likelihood)
#with an optimizer "optim"
> res1<-rma.glmm(measure="OR", ai=ai, bi=bi, ci=ci, di=di, model="CM.EL",
+control=list(optimizer="optim"))
Error in rma.glmm(measure = "OR", ai = ai, bi = bi, ci = ci, di = di,
:
Cannot fit ML model.
# a conditional generalized linear mixed-effects model (exact likelihood)
#with an optimizer "nlminb"
> res2<-rma.glmm(measure="OR", ai=ai, bi=bi, ci=ci, di=di, model="CM.EL",
+control=list(optimizer="nlminb"))
Error in rma.glmm(measure = "OR", ai = ai, bi = bi, ci = ci, di = di,
:
Cannot fit ML model.
# a conditional generalized linear mixed-effects model (exact likelihood)
#with an optimizer "bobyqa"
> res3<-rma.glmm(measure="OR", ai=ai, bi=bi, ci=ci, di=di, model="CM.EL",
+control=list(optimizer="bobyqa"))
Error in rma.glmm(measure = "OR", ai = ai, bi = bi, ci = ci, di = di,
:
Cannot invert Hessian for ML model.
In addition: Warning message:
In rma.glmm(measure = "OR", ai = ai, bi = bi, ci = ci, di = di,
:
```

```
Choleski factorization of Hessian failed. Trying inversion via QR decomposition.
# a conditional generalized linear mixed-effects model (exact likelihood)
#with an optimizer "newuoa"
> res4<-rma.glmm(measure="OR", ai=ai, bi=bi, ci=ci, di=di, model="CM.EL",
+control=list(optimizer="newuoa"))
Error in rma.glmm(measure = "OR", ai = ai, bi = bi, ci = ci, di = di,
:
Cannot invert Hessian for ML model.
In addition: Warning message:
In rma.glmm(measure = "OR", ai = ai, bi = bi, ci = ci, di = di,
:
Choleski factorization of Hessian failed. Trying inversion via QR decomposition.
###########################################################################
####################standard REM with DL method#################################
###########################################################################
res5<-rma(measure="OR", ai=ai, bi=bi, ci=ci, di=di,method='DL')
> res5

Random-Effects Model (k = 5; tau^2 estimator: DL)

tau^2 (estimated amount of total heterogeneity): 0 (SE = 0.1248)
tau (square root of estimated tau^2 value):      0
I^2 (total heterogeneity / total variability):   0.00%
H^2 (total variability / sampling variability):  1.00

Test for Heterogeneity:
Q(df = 4) = 1.5700, p-val = 0.8142

Model Results:

estimate      se     zval     pval    ci.lb    ci.ub
1.1743    0.1879   6.2504   <.0001   0.8061   1.5425     ***

###########################################################################
####################standard REM with REML method###############################
###########################################################################
res6<-rma(measure="OR", ai=ai, bi=bi, ci=ci, di=di,method='REML')
> res6

Random-Effects Model (k = 5; tau^2 estimator: REML)

tau^2 (estimated amount of total heterogeneity): 0 (SE = 0.1248)
tau (square root of estimated tau^2 value):      0
I^2 (total heterogeneity / total variability):   0.00%
H^2 (total variability / sampling variability):  1.00
```

```
Test for Heterogeneity:
Q(df = 4) = 1.5700, p-val = 0.8142

Model Results:

estimate      se     zval    pval    ci.lb   ci.ub
1.1743    0.1879   6.2504  <.0001  0.8061  1.5425     ***
```
